# Host Inflammatory Response to Mosquito Bites Enhances the Severity of Arbovirus Infection

**DOI:** 10.1016/j.immuni.2016.06.002

**Published:** 2016-06-21

**Authors:** Marieke Pingen, Steven R. Bryden, Emilie Pondeville, Esther Schnettler, Alain Kohl, Andres Merits, John K. Fazakerley, Gerard J. Graham, Clive S. McKimmie

**Affiliations:** 1 Virus Host Interaction Team, Section of Infection and Immunity, Leeds Institute of Cancer and Pathology, University of Leeds, Leeds LS9 7TF, UK; 2 Institute of Infection, Immunology and Inflammation, University of Glasgow, Glasgow G12 8TA, UK; 3 MRC-University of Glasgow Centre for Virus Research, Glasgow G61 1QH, UK; 4 Institute of Technology, University of Tartu, 50411 Tartu, Estonia; 5 The Pirbright Institute, Ash Road, Pirbright, Surrey GU24 0NF, UK

## Abstract

*Aedes aegypti* mosquitoes are responsible for transmitting many medically important viruses such as those that cause Zika and dengue. The inoculation of viruses into mosquito bite sites is an important and common stage of all mosquito-borne virus infections. We show, using Semliki Forest virus and Bunyamwera virus, that these viruses use this inflammatory niche to aid their replication and dissemination in vivo. Mosquito bites were characterized by an edema that retained virus at the inoculation site and an inflammatory influx of neutrophils that coordinated a localized innate immune program that inadvertently facilitated virus infection by encouraging the entry and infection of virus-permissive myeloid cells. Neutrophil depletion and therapeutic blockade of inflammasome activity suppressed inflammation and abrogated the ability of the bite to promote infection. This study identifies facets of mosquito bite inflammation that are important determinants of the subsequent systemic course and clinical outcome of virus infection.

## Highlights

• Mosquito bites enhance virus replication and dissemination and increase host mortality
• Neutrophil-driven inflammation retains virus in skin to drive macrophage recruitment
• Recruited and resident myeloid cells become infected and replicate virus
• Blocking leukocyte recruitment to bite site inhibits viral infection

## Graphical Abstract

**Figure:**
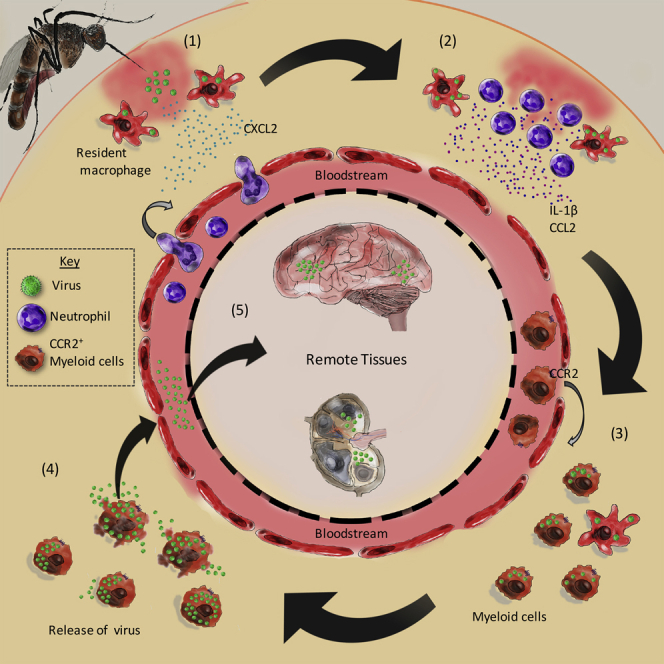

## Teaser

The inoculation of viruses into mosquito bite sites is an important and common stage of arbovirus infections. McKimmie and colleagues show that inflammation at bite sites aids viral replication and dissemination in vivo, resulting in more severe infection. These findings define additional targets for post-exposure prophylactic intervention.

## Introduction

The burden of mosquito-borne viral disease is profound. In recent years there has been a rapid increase in both the incidence and geographical range of such diseases, with spread to more temperate climates becoming more likely. Medically important viruses spread by arthropods (known as arboviruses) infect hundreds of millions of people each year (Bhatt et al., 2013, Weaver and Lecuit, 2015). This includes the chikungunya and Zika viruses that have recently triggered large-scale epidemics in the Americas (Burt et al., 2012, Gatherer and Kohl, 2016). The day-biting *Aedes* mosquitoes, and in particular *A. aegypti*, are the primary vectors. Arboviruses are an exceptionally large and diverse group of viruses (Elliott, 2014, Gould and Solomon, 2008, Powers et al., 2001). This heterogeneity, combined with the inability to accurately predict future arbovirus epidemics, makes developing and stockpiling specific drugs and vaccines very challenging.

All mosquito-borne viruses share a common attribute: their site of inoculation at mosquito bite sites. This aspect of their life cycle might provide a novel target for preventing diseases spread by this vector. In susceptible vertebrates, arbovirus replication in tissues results in a transient but very high level of infectious virus in the blood that is sufficient for a feeding arthropod to become infected. The high-level viraemia often induces a debilitating febrile illness and can result in the spread of virus to other tissues such as the brain, joints, and muscle. The early events of arbovirus infection are important for survival of the host, with a close relationship between early peripheral virus burden and mortality (Ryman and Klimstra, 2008). However, there remains a need to understand the determinants of early peripheral virus burden and what role cutaneous innate immune responses have in modulating viral replication and spread. It has previously been shown that mosquito bites enhance subsequent disease severity (Cox et al., 2012, Edwards et al., 1998, Limesand et al., 2000, Schneider et al., 2006). A similar observation has been made for ticks and biting flies (Dessens and Nuttall, 1998, Peters et al., 2008). When arboviruses are transmitted by mosquitoes, they replicate and disseminate more effectively to the blood, which may increase both their chance of onward transmission and their ability to cause more pronounced disease. The experimental deposition of uninfected mosquito saliva alone, in the absence of a bite, is sufficient to mediate this effect (Conway et al., 2014, Le Coupanec et al., 2013, Limesand et al., 2000, Moser et al., 2015, Styer et al., 2011). Although work has begun to define the factors within mosquito saliva that modulate arbovirus infection, the mechanistic basis that explains these observations is not known. Saliva from biting mosquitoes has been shown to have potent effects on various mammalian biological processes to support successful blood feeding (Fontaine et al., 2011), although evidence to support clearly defined immune-modifying functions in vivo is lacking. Here, we define the mechanistic basis by which mosquito bites enhance arbovirus infection in vivo.

Due to the importance of type I interferons (IFNs) and T cell responses in anti-viral immunity, their functional perturbation by mosquito saliva during infection has often been hypothesized (McCracken et al., 2014, Schneider et al., 2004). However, we showed that rather than perturb anti-viral immune responses, mosquito bites triggered a leukocyte influx that facilitated infection by providing new cellular targets for infection. We identified a two-step process in which mosquito bites caused an influx of inflammatory neutrophils that helped coordinate innate immune responses and pave the way for the chemokine receptor CCR2-dependent entry of myeloid cells that are permissive to viral infection. Furthermore, mosquito bites elicited a pronounced edema that retained more of the virus inoculum in the skin and facilitated infection of these cutaneous cells. Inhibition of key components of the inflammatory response to the bite reduced leukocyte influx, suppressed viral replication, and increased host survival. These findings identify an important aspect of the host immune response to mosquito bites that inadvertently promotes concurrent arbovirus infection.

## Results

### Mosquito Bites Enhance the Severity of Virus Infection

We developed an in vivo model system that makes use Semliki Forest virus (SFV), a mosquito-borne alphavirus that is a close relative of the chikungunya virus (Powers et al., 2001). We used *Aedes* mosquito cell-produced virus for inoculations to ensure that the first round of infection resembled the mosquito-transmitted virus as closely as possible. Unlike many human pathogens such as dengue and chikungunya viruses, SFV replicates and disseminates efficiently within both immune-competent mice and aedine mosquitoes (Ferguson et al., 2015, Rodriguez-Andres et al., 2012). SFV4 is an avirulent strain that rarely triggers clinical disease, whereas SFV6 is highly virulent and causes a lethal encephalitis (Ferguson et al., 2015, Michlmayr et al., 2014). After subcutaneous injection of SFV4 in the absence of mosquito bites, the virus rapidly disseminated to the draining popliteal lymph node (dLN) within 3 to 6 hr postinfection (hpi) (Figures S1A–S1D). A low-level viraemia peaked at 24 hpi with systemic spread to distal tissues apparent from 48 hpi onward, before occasional dissemination to the brain at 96 hpi (Figures S1A–S1C). Tissues identified by intravital imaging as positive for virus replication were dissected and further analyzed by quantitative (q)PCR to measure the level of viral RNAs (Figures S1B–S1D). The absence of detectable viral replication at remote sites before 48 hpi suggested that the majority of virus in the blood at 24 hpi was derived from cells infected at the inoculation site and the lymphoid tissue that drains it.

**Figure 1:**
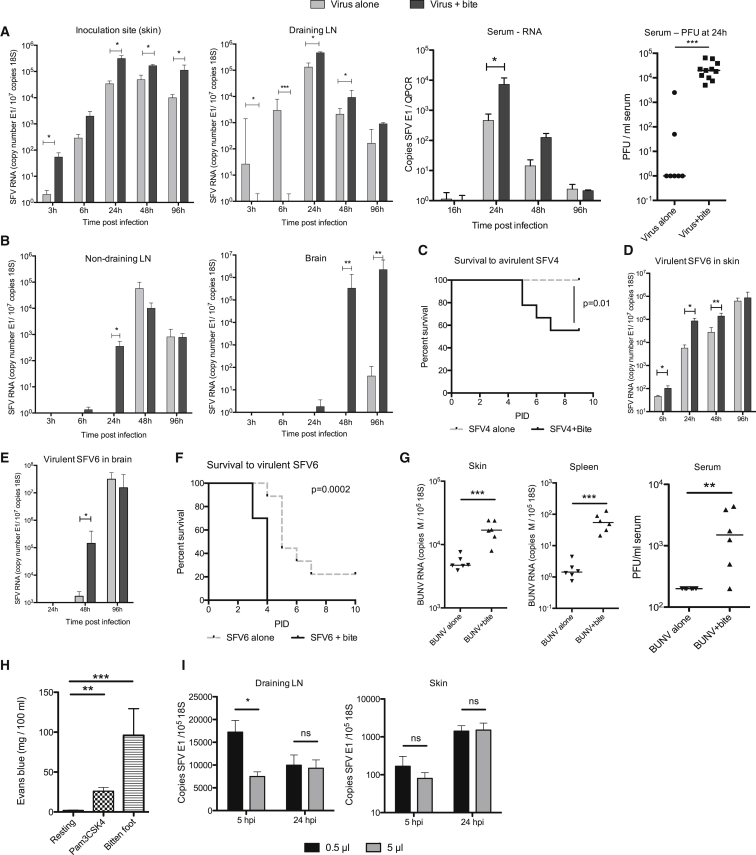
Mosquito Bites Facilitate Virus Retention and Replication at Cutaneous Inoculation Sites, Enhance Viraemia and Systemic Dissemination, and Increase Mortality to SFV Infection (A and B) Mice were infected with 10^3^ PFU of SFV4 either in presence (dark gray bars) or absence (light gray bars) of a mosquito bite. Copy number of SFV RNA (E1 gene) and host 18S was determined by qPCR. Virus titers in the serum were also quantified by plaque assay (n ≥ 7). (C) Mice were infected either in the presence (black line) or absence (grey dotted line) of a mosquito bite with SFV4 (n = 10). (D and E) Mice were infected with 250 PFU SFV6 in presence (dark gray bars) or absence (light gray bars, n = 5) of a mosquito bite. (F) Mice were infected either in the presence (black line) or absence (grey dotted line) of a mosquito bite with SFV6 (n = 10). (G) Mice were infected with 10^4^ PFU BUNV in presence or absence of a mosquito bite (n = 6) and the level of viral RNA and infectious titer quantified at 24 hpi by qPCR and plaque assay. Gene expression of the virally encoded M-segment was used to assay BUNV RNA. (H) Mice were injected with either saline or TLR2 ligand Pam3CSK4 or exposed to mosquito bites and edema determined at 3 hpi (n = 8). (I) Mice were infected with 10^3^ PFU SFV4 constituted in differing volumes and SFV E1 RNA copy number determined by qPCR (n = 8). All column plots show the median value ± interquartile range. Results shown are representative of either two or three experiments. ^∗^p < 0.05, ^∗∗^p < 0.01, ^∗∗∗^p < 0.001. See also Figure S1.

To determine whether mosquito bites had the ability to affect virus infection in this model, mice were bitten with *A. aegypti* mosquitoes and then immediately infected with a defined dose of SFV4 at the bite site in a 1 μL volume (Figures 1A and 1B). By clearly defining the number of mosquito bites to a restricted area of skin and by injecting a known titer of virus inoculum, it was possible to reproducibly infect either mosquito-bitten skin or resting unaffected skin. The presence of mosquito bites resulted in an order of magnitude higher virus RNA copy number in the skin at the inoculation site at most time points compared to unbitten mice and in the dLN and blood from 24 hpi onward. Virus infection with mosquito bites mediated earlier and greater dissemination of virus to remote lymphoid tissue and to the brain (Figure 1B) and in a significant proportion of the mice converted an avirulent infection into a lethal one (Figure 1C). In comparison, infection with the SFV6 strain of virus was fatal irrespective of the presence of mosquito bites at the inoculation site. As with SFV4, bites enhanced SFV6 infection at early time points, facilitating a more rapid dissemination to the brain, and mice succumbed earlier to infection (Figures 1D–1F and S1E). Mosquito bites also significantly enhanced infection with Bunyamwera virus (BUNV) (Figure 1G), a genetically unrelated RNA negative-sense virus that is also transmitted by *Aedes* mosquitoes (Elliott, 2014). BUNV, which otherwise struggles to replicate in wild-type mice after extraneural inoculation (Bridgen et al., 2001), demonstrated significantly higher replication and systemic spread to distal tissues when inoculated into mosquito bites. These results indicate that mosquito bites enhanced BUNV infection in vivo. Together these data suggest that early skin-centric processes triggered by mosquito bites have a substantial and defining impact on virus replication in vivo and that this impacts end-stage disease.

### Mosquito Bite Sites Are Characterized by Extensive Edema that Is Associated with Retention of Viral Inoculum at the Bite Site

In the absence of bites, SFV disseminated rapidly to the dLN where copy number of virus RNA correlated with type I IFN expression in a time-dependent manner (Figure S1F). The presence of bites inhibited initial transfer of virus to the dLN (Figure 1A), which correlated with a delayed induction of gene transcripts for the prototypic IFN-stimulated gene (ISG) CXCL10 and also IFN-γ (Figure S1G). However, by 24 hpi, type I IFN and ISGs in the dLN were elevated compared to unbitten mice, correlating with higher virus RNA copy number at this time point (Figures 1A and S1H). To determine whether altered fluid flow within bitten skin could account for the suppression of early viral dissemination to the dLN at 6 hr, we quantified the level of vascular leakage at bite sites by injecting mice with Evans blue dye and measuring the amount dye that leaked from the circulation into the bite site (Figure 1H). At 3 hr after mosquito bite, the concentration of Evans blue detected in bitten skin was significantly higher compared to both resting skin and skin inflamed with the Toll-like receptor 2 (TLR2) agonist Pam3CSK4, a known inducer of edema (Supajatura et al., 2002). This suggested that a substantial bite-associated edema occurred that was associated with retention of virus in the skin. We hypothesized that the increased volume of tissue fluid delayed drainage of free virus, and that its retention was likely to facilitate infection of cutaneous cells and could also explain the delayed innate immune activation in the dLN. To determine whether increased interstitial fluid alone could account for the delayed dissemination of virus to dLN, unbitten mice were inoculated with the same dose of virus in either a small volume (0.5 μL) or large volume (5 μL) inoculum of virus in albumin-containing saline. Those mice that received the virus in the larger volume demonstrated impaired spread of virus to the dLN at 5 hpi, although viral RNA copy number were similar at later time points (Figure 1I). In summary, mosquito bites induced an edema that retained virus in the skin, facilitating infection of cutaneous cells, but increased interstitial fluid alone is unlikely to account for enhancement of infection by mosquito bites at later time points.

### Mosquito Bites and Virus Infection Combine to Induce a Substantial but Transient Increase in Cutaneous Neutrophils

**Figure 2:**
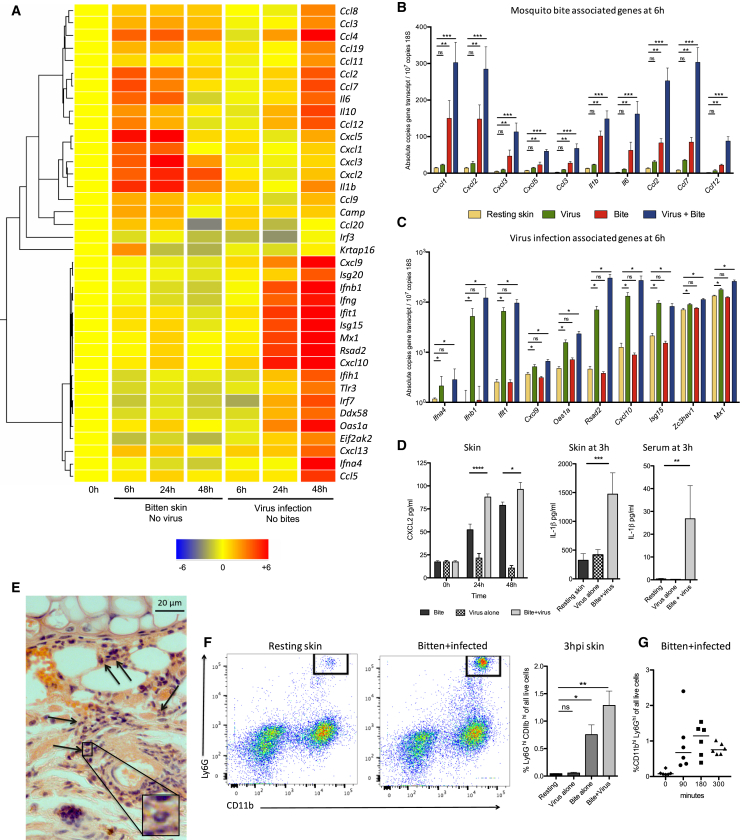
Mosquito Bites Induce the Rapid Recruitment of Neutrophils (A) Cutaneous immune responses to bites alone, without concurrent virus infection, were compared to mice infected with SFV4 alone, in the absence of bites. Gene expression was determined using Taqman low-density arrays and hierarchical clustering undertaken to group genes with similar patterns of expression. (B and C) Bite-associated gene expression (B) and SFV4 infection-associated genes (C) at 6 hr were validated by absolute qPCR (n ≥ 6). (D) CXCL2 (n = 6) and IL-1β protein levels (n = 10) were determined by ELISA in skin samples from uninfected and SFV4-infected mosquito bite sites. (E and F) Neutrophil infiltration was determined by histology (E) and flow cytometry (F) at 3 hr after bite or infection with SFV4. (E) Tissue sections stained by H&E. Black arrows indicate typical multi-lobed nuclei of neutrophils. (F) Numbers represent percent of CD11b^hi^Ly6G^hi^ cells of all live cells (n = 4). (G) Neutrophils were present in high numbers by 90 min after bite/infection, peaking at 180 min. All column plots show the median value ± interquartile range. Results shown are representative of either two or three experiments. ^∗^p < 0.05, ^∗∗^p < 0.01, ^∗∗∗^p < 0.001. See also Figures S2–S4.

To determine the mechanism by which mosquito bites enhanced virus infection, we next defined the effect that bites alone had on cutaneous innate immune responses. The expression of 64 key innate immune genes were assayed in the skin of mice that were either bitten with mosquitoes or infected with virus in the absence of mosquito bites. Hierarchical clustering identified a distinctive innate immune signature in bite sites that was dominated by neutrophil-attracting chemokines (CXCL1, CXCL2, CXCL3, and CXCL5) and the cytokines IL-1β and IL-6, which were not significantly induced by virus infection alone (Figures 2A–2C and S2). Genes also upregulated by bites included the monocytic chemoattractive chemokines CCL2, CCL4, CCL5, CCL7, and CCL12. This expression pattern was similar in bitten mice that had previously been exposed to multiple mosquito bites (Figure S3). In comparison to mosquito-bitten skin, the cutaneous gene expression signature of virus infection alone was both markedly delayed and dominated by type I IFN and ISGs (Figures 2A–2C and S2).

Induction of CXCL2 and IL-1β by bites was compared to other known inducers of neutrophil influx and to virus infection alone (Figures S4A and S4B). Despite the relatively discrete area of skin affected by biting, compared to that affected by injection of other innate immune stimulants, bites resulted in a substantial increase in gene transcripts, which was further validated at the protein level by ELISA (Figure 2D). Accordingly, mosquito bites elicited a substantial infiltrate of neutrophils from as early as 90 min onward, as demonstrated by histology (Figure 2E), flow cytometry analysis of Ly6G^hi^CD11b^hi^CD45^+^ neutrophils (Figures 2F and 2G), and qPCR analysis of neutrophil-specific markers (Figure S4E). Despite the inability of virus infection alone to induce substantial expression of CXCL2 or IL-1β, or Ly6G^hi^CD11b^hi^CD45^+^ neutrophil influx, infection combined with bites induced significantly higher expression and an enhanced neutrophil influx compared to bite alone (Figures 2D and 2F). Thus, although virus infection alone was not sufficient to induce the expression of most bite-associated genes, infection did nonetheless combine with mosquito bites in the induction of bite-associated genes and triggered a more pronounced neutrophil influx. In summary, neutrophil influx is a key aspect of virus-infected bite sites and that this is associated with enhanced viral replication. One possible explanation is that newly recruited neutrophils support viral replication by providing additional cellular target for virus infection, as is the case for *Leishmania* transmission by sandflies (Peters et al., 2008). However, we found that neutrophils at bite sites were refractory to infection (Figure S4F).

### Mosquito Bites Do Not Subvert the Induction of Antiviral Immune Responses

**Figure 3:**
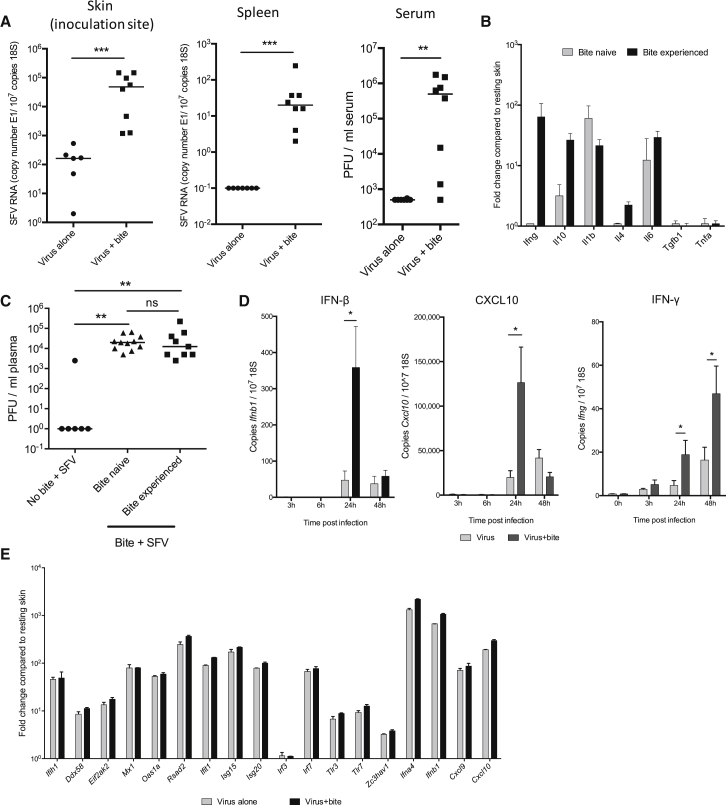
Mosquito Bites Do Not Subvert the Induction of Antiviral Immune Responses (A) SCID mice were infected with 10^4^ PFU SFV4, either in presence or absence of mosquito bites and the levels of viral RNAs determined (n = 8). (B and C) Wild-type mice were either subjected to four sessions of mosquito biting at 1-week intervals or left unexposed to mosquitoes (bite-naive). Mice were then subjected to mosquito biting and gene transcripts assayed in skin at 6 hr after bite (B) or infected with 10^4^ PFU SFV4 into the bite site and serum viraemia determined at 24 hpi (C) (n ≥ 6). (D and E) Cutaneous type I IFN and ISG gene expression was enhanced by the presence of mosquito bites. Mice (n ≥ 5) were infected with SFV4 either in presence or absence of mosquito bites and the cutaneous expression of transcripts determined by absolute qPCR (D), or fold change determined by TLDA (E). Fold change was calculated by comparison to resting skin and was normalized to the level of SFV E1 copy number. All column plots show the median value ± interquartile range. ^∗^p < 0.05, ^∗∗^p < 0.01, ns = not significant.

Mosquito saliva contains a myriad of biologically active components such as vasodilators (Fontaine et al., 2011) and it has been hypothesized that it also has immune-modulating factors that facilitate host infection by a variety of pathogens (Schneider et al., 2004). Indeed, much effort is being made to identify these factors from a variety of blood-feeding arthropods (Déruaz et al., 2008, King et al., 2011). It has been suggested that mosquito bites promote a T helper 2 (Th2)-cell-dominated immune response that enhances virus replication (Schaeffer et al., 2015, Schneider et al., 2004). Although our kinetic data (Figure 1) and those of others (Styer et al., 2011) suggest that enhancement of infection by mosquito bites occurs too early for adaptive immunity in naive mice to impact on this process, we nevertheless looked at this possibility. Bite-associated enhancement of infection was apparent in severe combined immunodeficiency (SCID) mice, which lack T and B cells (Figure 3A), whereas classic Th1 or Th2 cytokines (e.g., IFN-γ, IL-4) could not be detected after mosquito biting of naive wild-type mice in the absence of virus infection (Figure S2). Prior exposure of mice to mosquito bites primed them to rapidly express cutaneous IFN-γ and IL-10 upon further mosquito biting (Figure 3B). However, we found that these bite-experienced mice did not demonstrate any increased susceptibility to, or protection from, bite enhancement of SFV4 infection compared to bite-naive mice (Figure 3C). Thus, mosquito bite enhancement of virus infection is independent of host cutaneous IFN-γ and does not require adaptive immunity.

We next determined whether mosquito bites suppressed cutaneous anti-viral innate immune responses. We assayed the transcriptional induction of IFNs and ISGs known to have important anti-viral functions (Figures 3D and 3E; Schoggins et al., 2011). However, rather than suppress cutaneous IFN-β induction to virus, mosquito bites resulted in enhanced cutaneous IFN-β transcript induction at 24 hpi (Figure 3D), probably as a direct result of higher virus replication as indicated by increased virus RNA copy numbers (Figure 1). Indeed, once the higher copy numbers of virus RNA present in the skin were accounted for, ISG induction by virus was mostly unaffected by mosquito bites (Figure 3E), suggesting that bites do not facilitate virus infection by suppressing the cutaneous induction of IFNs. All together these results indicate that mosquito bite enhancement of virus infection is not due to suppression or subversion of skin anti-viral immune responses by bites.

### Neutrophils Drive a Cutaneous Pro-inflammatory Program that Inadvertently Facilitates Virus Infection

**Figure 4:**
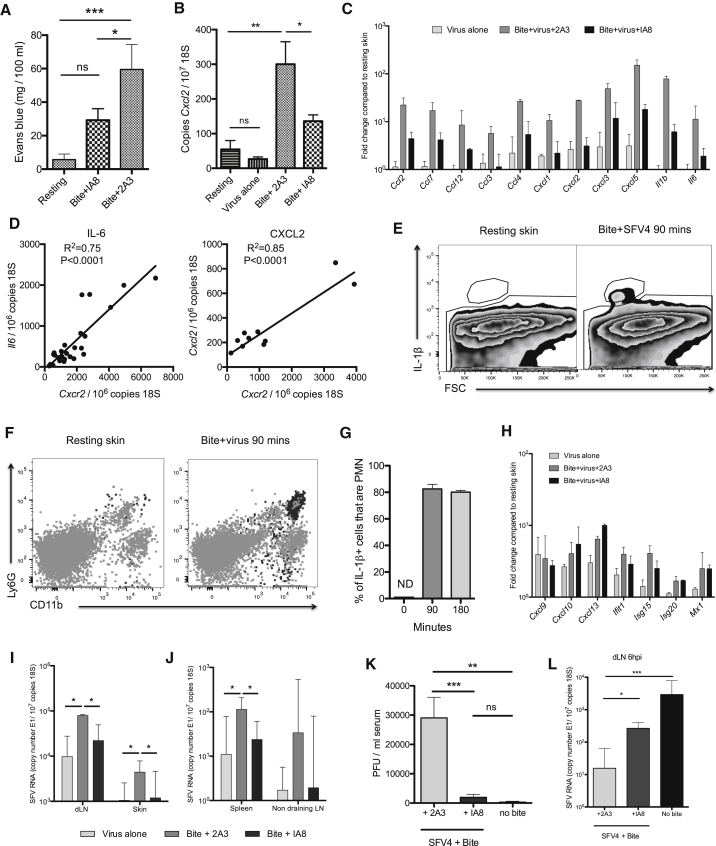
Mosquito Bite-Infiltrating Neutrophils Express IL-1β, Co-ordinate Innate Immune Gene Expression, and Are Required for the Effective Replication and Dissemination of Virus (A–C) Mice were either depleted of neutrophils by i.p injection of the IA8 antibody or control treated with non-depleting 2A3 control antibody, bitten with mosquitoes, and infected with 10^4^ PFU SFV4 (n ≥ 6). (A) Edema in skin was quantified after i.p. injection of evans blue at 4 hpi. (B) CXCL2 transcripts were determined by qPCR in the skin of mice at 6 hr, n = 12. (C) Skin-bite-associated genes were assayed by qPCR at 6 hpi. Untreated, SFV4-infected mice were included for comparison. (D) IL-6 (n = 26) and CXCL2 (n = 12) transcripts correlated with the number of CXCR2 transcripts in mosquito-bitten skin. (E–G) Skin biopsies at 90 min after bite or SFV4 infection were digested to release cells and stained for flow cytometry. Cells were gated based on their IL-1β staining (E) and back-gated onto a Ly6G/CD11b plot as dark gray dots (F). The percent of IL-1β cells that were Ly6G^hi^CD11^hi^ at 90 min after bite/infection (n = 6, ND = not detected) (G). (H) Skin virus-associated genes were assayed by qPCR at 6 hpi. Untreated, SFV4-infected mice were included for comparison. (I–K) At 24 hpi, SFV RNA copy number was determined in dLN and inoculation site (skin) (I) and in lymphoid tissues distal to inoculation site (J) by qPCR, n = 7, and infectious virus in the serum determined (K) n = 10. (L) SFV RNA was quantified at 6 hr in the draining popliteal LN to determine whether neutrophil depletion could overcome the initial block imposed by bites on early virus dissemination (n = 7). All column plots show the median value ± interquartile range. Results shown are representative of either two or three experiments. See also Figure S5.

We hypothesized that if recruited neutrophils are not the target of virus infection themselves, they might nonetheless be instrumental in coordinating the tissue response to mosquito bite trauma that supports enhanced virus infection. Neutrophils have long been established as pivotal regulators of vascular permeability, edema, and the influx of myeloid cells (Charmoy et al., 2010, Nakamura et al., 2009, Wedmore and Williams, 1981). Although much of the vascular leakage at bites is probably due to vessel rupture by probing mosquitoes, neutrophils enhance this leakage, as shown by the fact that depletion of neutrophils (using the IA8 antibody that binds neutrophil-expressed Ly6G) significantly reduced edema (Figures 4A and S5A). Depletion of neutrophils also greatly impaired the induction of many bite-associated genes at 6 hpi (Figures 4B and 4C), whereas in non-depleted mice, neutrophil-expressed CXCR2 copy number correlated with many of these genes (Figure 4D), suggesting that neutrophil influx into the bite site was required for their induction. Bite-associated genes that required neutrophil influx included IL-1β and the monocyte-attracting chemokines CCL2, CCL7, and CCL12 (Figure 4C). Almost all IL-1β-expressing cells at bite sites were positive for Ly6G and CD11b by flow cytometry, suggesting that infiltrating neutrophils themselves were the main cellular source of this cytokine (Figures 4E–4G). In comparison, those genes identified as specifically upregulated by virus infection alone in Figure 2, were unaffected by neutrophil depletion at 6 hpi (Figure 4H). In summary, inflammatory neutrophils that express IL-1β are required for the induction of cutaneous inflammatory responses to mosquito bites.

Next, we determined whether neutrophil-dependent inflammation is required for bite enhancement of infection. Depletion of neutrophils reduced SFV4 E1 RNAs by 5-fold at both the skin inoculation site, dLN, and distal lymphoid tissue at 24 hpi (Figures 4I and 4J), while viraemia was reduced 10-fold, such that it was not significantly different to unbitten controls (Figure 4K). Furthermore, the early dissemination of virus to dLN observed in unbitten mice was also partially restored in bitten mice in the absence of neutrophils, suggesting that lymph flow had transported more of the initial inoculum to the draining LN (Figure 4L). Thus, mosquito bites triggered a seemingly counter-productive neutrophil-dependent response that enhanced SFV infection at cutaneous inoculation sites.

**Figure 5:**
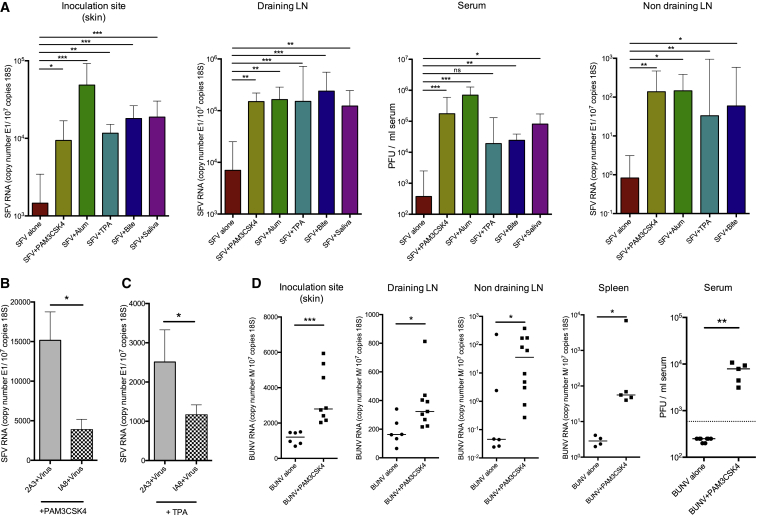
Structurally Unrelated Pro-inflammatory Agents and Mosquito Saliva Promote Virus Infection (A) After s.c. administration of TLR2 ligand Pam3CSK4, alum, or mosquito saliva or after mosquito biting or topical application of the phorbol ester TPA, mice were infected with 10^4^ PFU SFV4 at the same site (n ≥ 5), and the level of viral RNA determined by qPCR and infectious titer determined by plaque assay at 24 hpi. (B and C) Mice were depleted of neutrophils using the IA8 antibody or given 2A3 control antibody, treated with a s.c. injection of Pam3CSK4 (B) or topical application of TPA (C), and then infected with 10^4^ SFV4 at the same cutaneous site. At 24 hpi, SFV RNA copy numbers were determined in the skin (n = 5). (D) After s.c. administration of the TLR2 ligand Pam3CSK4, mice were infected with 10^4^ BUNV, and the level of viral RNA (M-segment) and infectious titer quantified at 24 hpi. All column plots show the median value ± interquartile range. ^∗^p < 0.05, ^∗∗^p < 0.01, ^∗∗∗^p < 0.001, ns = not significant. See also Figure S6.

To ascertain whether this effect is specific to mosquito bites, we determined whether innate immune agonists that also trigger neutrophil influx could similarly enhance infection. These included a phorbol ester (TPA), alum, and the TLR2 ligand Pam3CSK4, all of which induced cutaneous CXCL2 and IL-1β expression in our mice (Figures S4A and S4B). We compared the ability of these agents to enhance SFV4 infection to that of mosquito bites and separately to that of injected mosquito saliva, which had been previously obtained from female *A. aegypti* mosquitoes. All these agents substantially enhanced SFV4 replication in the skin and dissemination to the blood despite them having no known structural similarity to each other (Figure 5A). Furthermore, as with mosquito bites, enhancement of SFV4 infection by Pam3CSK4 and phorbol ester occurred in a neutrophil-dependent manner (Figures 5B and 5C) and was not due to a suppression of type I IFN induction (Figure S6). Pam3CSK4 was also able to significantly enhance infection with the unrelated BUNV (Figure 5D). Together this suggests that mosquito-sourced factors at bite sites do not enhance infection via any specific evolved function, but instead by their inadvertent ability to promote a neutrophil-dependent inflammation after a bite.

### Therapeutic Inhibition of the Inflammasome Inhibits Neutrophil Influx and Prevents Bite Enhancement of Virus Infection

We next determined whether it was possible to alter the outcome of infection by modulating host immune responses to bites. Because neutrophil depletion was so effective at suppressing bite enhancement of infection (Figure 4), we first determined whether neutrophil depletion could also decrease mortality with SFV6 infection. However, we found that neutrophils were required for protection from SFV6 infection irrespective of the presence of mosquito bites, with roughly 50% of neutrophil-sufficient mice surviving infection, compared to 10% of mice surviving in the absence of neutrophils (Figure S5C). Together these data suggest that although neutrophils initiate counterproductive responses at mosquito bites for the host, they are nonetheless required at later stages of disease to prevent mice from succumbing to infection.

**Figure 6:**
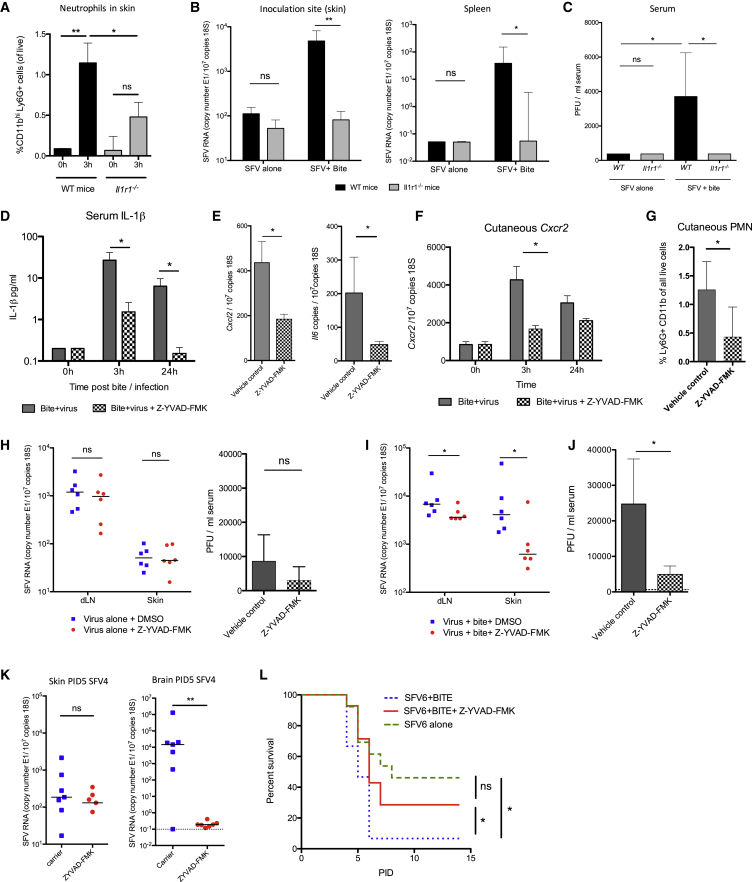
Mosquito Bite Enhancement of Virus Infection Is Dependent on IL-1β (A) Numbers of neutrophils infiltrating SFV4-infected bite sites were quantified in WT and *Il1r1*^−/−^ mice (n = 6). (B and C) WT and IL-1R-null mice (n ≥ 6) were infected with 10^4^ SFV4 at mosquito bite sites or resting skin. SFV RNA copy numbers (B) and viraemia were determined (C) at 24 hpi. (D–G) Mice were treated with the caspase-1 inhibitor Z-YVAD-FMK i.p. (1.5 mg/kg) or vehicle control, bitten by mosquitoes, and then infected with 10^4^ SFV4 at the bite site. (D) Serum IL-1β expression was determined by ELISA (n = 10). (E) Cutaneous bite-associated innate immune transcripts CXCL2 and IL-6 were determined by qPCR at 3 hpi (n = 5). (F and G) Neutrophil influx into bite sites (n ≥ 6) was ascertained by assaying cutaneous CXCR2 expression (F) and the frequency of Ly6G^hi^CD11b^+^SSC^hi^ cells by flow cytometry at 3 hpi (G). (H–K) Mice were treated with Z-YVAD-FMK i.p. and infected with 10^4^ SFV4 in the absence (H) or presence (I–K) of mosquito bites. SFV RNA was determined by qPCR at 24 hpi (I) and at PID5 (K). Viraemia at 24 hpi was determined by plaque assay (J) (n = 6). (L) Survival curve of mice infected with SFV6 at bite sites with or without treatment with caspase-1 inhibitor Z-YVAD-FMK i.p. at time of infection (n = 15). Results shown are representative of either two or three experiments. All column plots show the median value ± interquartile range. ^∗^p < 0.05, ^∗∗^p < 0.01.

Although the wholesale depletion of neutrophils is clearly inappropriate for preventing bite-enhanced SFV infection, a more refined interventionist approach would be to reduce neutrophil recruitment to bitten skin while leaving systemic neutrophil numbers untouched. Because neutrophil-derived IL-1β expression was a key feature of mosquito bites and because inflammasome activation has been implicated in enhancing neutrophil influx (Nakamura et al., 2009), we wanted to determine whether it was possible to target this pathway. We first wanted to confirm that mosquito bite enhancement of virus infection was dependent specifically on IL-1β. Accordingly, *Il1r1*^−/−^ mice demonstrated both a deficiency in neutrophil recruitment to infected bite sites and were not susceptible to bite-mediated enhancement of virus infection (Figures 6A–6C), identifying this pathway as a putative therapeutic target.

We next determined the efficacy of the well-characterized caspase-1-specific antagonist Z-YVAD-FMK (Jabir et al., 2014) in preventing bite enhancement of virus infection. After Z-YVAD-FMK treatment, mosquito-bitten virus-infected mice demonstrated significantly less serum IL-1β (Figure 6D), a reduction in the cutaneous expression of bite-associated CXCL2 and IL-6 (Figure 6E), and fewer cutaneous neutrophils (Figures 6F and 6G). We next determined whether the innate immune suppression of bite-associated inflammation provided by inflammasome inhibition affected the replication and dissemination of SFV. Importantly, Z-YVAD-FMK administration had no effect on viral titers by 24 hpi in the absence of mosquito bites (Figure 6H), suggesting that this agent has no intrinsic anti-viral effect. However, inflammasome inhibition was highly efficacious at lowering viral titers by 24 hpi in mice infected at mosquito bite sites (Figures 6I and 6J). Copies of SFV4 RNA in the brain at day 5 were low or undetectable in treated mice, whereas treated SFV6-infected mice had an increased survival rate (Figures 6K and 6L). Together, these observations show that the IL-1β pathway promoted mosquito bite inflammation, that this was necessary for the effective replication and dissemination of virus from the bite site, and that these in turn determine the subsequent systemic course of infection and clinical outcome in the mouse.

### CCR2-Dependent Migration and Infection of Myeloid Cells Is Required for Mosquito Bite Enhancement of Infection

**Figure 7:**
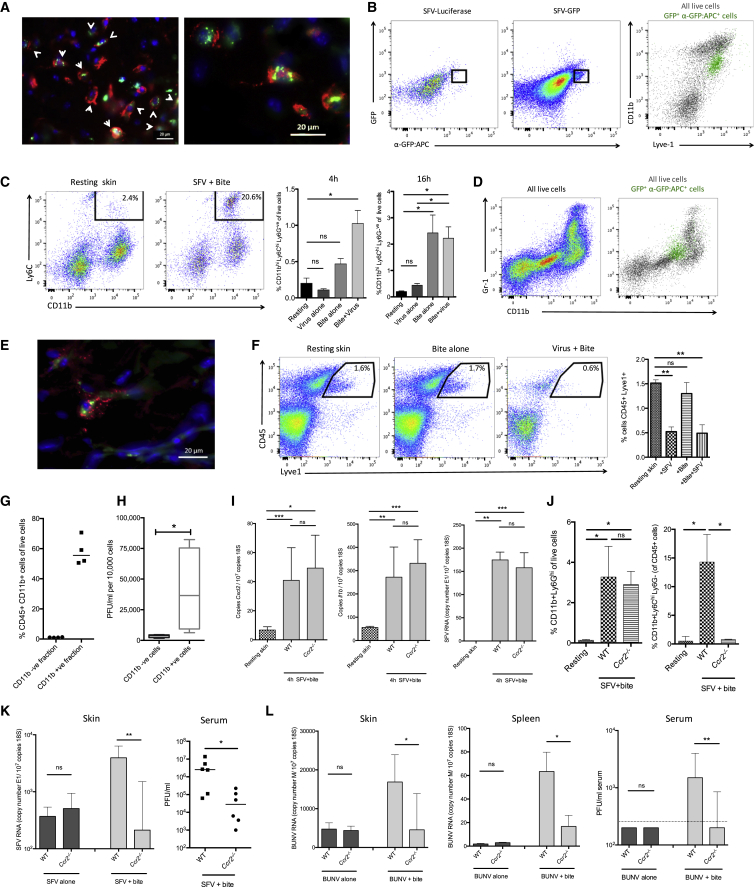
CCR2-Dependent Migration and Infection of Myeloid Cells Is Required for Mosquito Bite Enhancement of Virus Infection (A) Mice were infected intradermally with SFV4(Xho)-EGFP (green) in back dorsal skin and at 6 hpi sections stained for lyve1 (red) and DAPI (blue). Arrows indicate double-positive cells in lower magnification image. (B) Skin SFV4(Xho)-EGFP-infected cells were identified by both their expression of EGFP and staining against EGFP and back-gated onto a CD11b/Lyve1 plot, represented as green dots. Luciferase-expressing SFV4 was used as a control. (C) Quantification of CD11b^+^Ly6C^hi^ myeloid cell numbers in bite sites at 4 and 16 hr. Live cells were first gated for CD45^hi^ cells and to remove Ly6G^hi^ neutrophils (n = 4). (D) CD11b and Gr-1 staining for all cells (left) and back gating of virus-infected cells at 16 hpi (green dots, right plot). (E) Skin sections were stained for the bone-marrow-derived myeloid marker ER-HR3 (red) in SFV4(Xho)-EGFP-infected skin at 16 hpi. (F) Percentage CD45^+^Lyve1^+^ macrophages numbers of all live cells at 24 hpi in the skin (n = 4). (G and H) SFV4-infected bite sites (n = 4) were digested at 16 hpi to release cells and CD11b^+^ cells sorted on columns to generate a CD11b^−^ fraction and a CD11b^+^ enriched fraction (G). Infectious virus released by each cell fraction after 6 hr in culture was quantified by plaque assay (H). (I) Gene transcripts for bite-associated innate immune genes and viral RNA at 4 hpi (n ≥ 7). (J) Level of neutrophils at 4 hpi and monocytes at 18 hpi in the skin were determined by flow cytometry (n ≥ 4). (K and L) *Ccr2*^*−/−*^ mice are protected from bite-enhanced arbovirus infection at 24 hpi. Wild-type and *Ccr2*^*−/−*^ mice were infected with either 10^4^ SFV4 (K) or 10^4^ PFU of the genetically unrelated arbovirus BUNV (L) in the presence or absence of a mosquito bite. (K) qPCR analysis of SFV RNA at 24 hr in the skin and viraemia at 24 hpi (n = 6). (L) qPCR analysis of BUNV M-segment RNA at 24 hr in the inoculation site (skin), remote tissue (spleen), and viraemia at 24 hpi (n = 6). All column plots show the median value ± interquartile range. ^∗^p < 0.05, ^∗∗^p < 0.01, ^∗∗∗^p < 0.001, ns = not significant. Results shown are representative of either two or three experiments. See also Figure S7.

Finally, we wanted to determine the mechanism by which inflammation augmented virus infection. We first defined which cell types were responsible for supporting enhanced viral replication at bite sites. Lyve1 is a protein expressed by both lymphatic endothelial cells and CD45^+^F4/80^+^CD11b^+^ macrophages in the skin, which can be differentiated from each other based on their morphology (Schledzewski et al., 2006) and by flow cytometry (Figure S7). After infection with enhanced green fluorescent protein (EGFP)-expressing SFV, we found large numbers of EGFP^+^Lyve1^+^ cells in the dermis (Figure 7A). Lymphatic endothelial cells did not express EGFP (data not shown), although Lyve1^+^ macrophages were positive for SFV-EGFP at 6 hpi (Figures 7A and 7B). Between 4 and 16 hpi, large numbers of CD11b^+^Ly6C^+^Ly6G^−^ myeloid cells infiltrated bite sites and numbers of these cells were increased in virus-infected bite sites, whereas in the absence of bites, virus infection alone elicited little influx of these cells (Figure 7C). A subset of these cells were positive for virally encoded EGFP (Figures 7D and 7E). We found that by 24 hpi in virus-infected bite sites, there was a selective loss of Lyve1^+^F4/80^+^CD11b^+^ cells, whereas numbers did not decrease in bite sites in the absence of virus, suggesting that they had been depleted after infection with this often cytolytic virus (Figures 7F and S7B).

Infection of myeloid cells and dendritic cells in skin has also been reported during infection with dengue virus in the absence of mosquito bites (Pham et al., 2012, Schaeffer et al., 2015, Schmid and Harris, 2014), suggesting that several arboviruses might have evolved to take advantage of the myeloid cell infiltrate at bite sites to enhance replication. To determine whether infection of myeloid cells at bite sites results in the release of new infectious virus, we isolated cutaneous cells at 16 hpi, magnetically purified CD11b^+^ cells on columns (Figures 7G and S7C), and quantified the amount of virus released after 6 hr in culture by plaque assay (Figure 7H). On a per-cell basis, the CD11b^−^ fraction released little new infectious virus and the CD11b^+^ enriched fraction released substantially more, demonstrating that CD11b^+^ cells are capable of releasing high amounts of infectious virus.

To determine the contribution that myeloid cell-derived virus makes toward viral replication and dissemination in vivo, we made use of mice that lack the chemokine receptor CCR2. These mice are monocytopenic and are deficient in all dermal bone marrow-derived macrophages (Serbina and Pamer, 2006, Tamoutounour et al., 2013). Compared to WT mice, infection of *Ccr2*^−/−^ mice at bite sites resulted in similar increases in innate immune gene expression and neutrophil influx and demonstrated similar virus titers at 4 hpi but failed to elicit an influx of myeloid CD11b^+^Ly6C^hi^ cells (Figures 7I, 7J, S7D, and S7E). *Ccr2*^−/−^ mice were protected from bite enhancement of SFV replication (Figure 7K). Mosquito bites also significantly enhanced infection with BUNV in a CCR2-dependent manner (Figure 7L). *Ccr2*^−/−^ mice were not intrinsically less susceptible to infection because in the absence of bites, SFV and BUNV titers were similar to WT mice at 24 hpi (Figures 7K and 7L). Together this demonstrates that the inability of *Ccr2*^−/−^ mice to elicit a myeloid cell influx into bite sites prevented enhancement of infection. In summary, we show that mosquito bites enhance mosquito-borne virus infection by promoting the recruitment and infection of bone marrow-derived myeloid cells that release new infectious virus and in doing so enhance the replication and dissemination of virus to the blood and remote tissues.

## Discussion

The interface between mammals and mosquitoes at bite sites is an important and common stage of all arbovirus infections. This crucial aspect of arbovirus transmission is a bottleneck that can limit dissemination of virus to the bloodstream and the development of clinically apparent disease. Although mosquito saliva is well described as a potent enhancer of infection for several evolutionary distinct and medically important arboviruses, the cellular and molecular events that are responsible were not well defined. Previously, it had been suggested that mosquito saliva might modulate host immunity to create an immunosuppressed niche, as has been shown for biting ticks that suppress chemokine function (Déruaz et al., 2008). Ticks have evolved a unique repertoire of immunosuppressive factors, which most likely reflects their necessity to remain embedded in mammalian skin for many days. In comparison, mosquitoes feed only transiently and host immune responses are unlikely to exert sufficient effect on feeding efficiency to drive the evolution of vector-derived immunomodulatory factors. Instead we show that mosquito bite enhancement of virus infection results from virus replication in myeloid cells recruited to mosquito bites by a neutrophil-driven inflammasome-dependent, edematous inflammation. Whether the recruitment to bite sites of these cells is serendipity for the virus or whether the virus has evolved to replicate in these cells is not clear. However, the consequences are profound and affect the subsequent systemic course and clinical outcome of the infection. That this phenomenon occurs with two genetically distinct arboviruses from separate families suggests that it might also occur with many other mosquito-transmitted viruses.

We found that the neutrophil influx to mosquito bites was essential for coordinating the tissue response to this insult, without which innate immune gene expression was severely curtailed. This is somewhat similar to host responses to *Leishmania* infection, in which neutrophil recruitment is necessary for driving chemokine CCL3-dependent influx of dendritic cells to the skin (Charmoy et al., 2010). Separately, although we cannot discount the possibility that putative Th2-cell-driven allergic responses to bites in mosquito-experienced BALB/c mice (Schneider et al., 2007) might also a have role in potentiating virus infection, the ability of bites to fully potentiate virus infection in SCID mice suggests that they are dispensable in our model system.

Therapeutic blockade of caspase-1 and neutrophil depletion successfully suppressed cutaneous innate immune responses to bites and thus prevented virus from gaining a replicative advantage. Speculatively, suppressing specific aspects of the host response to mosquito bites might prove efficacious in preventing the onset of disease. We suggest a strategy that minimizes mosquito biting (e.g., use of repellents) combined with post-exposure prophylaxis at bite sites as an effective strategy for limiting mosquito-borne virus infection, especially for infections transmitted by *Aedes* mosquitoes that preferentially bite during the day. As a corollary, our data also suggest that approaches that aim to prevent natural arbovirus infection through vaccination to mosquito salivary components, while having much potential, should be carefully designed not to enhance cutaneous inflammation, and might explain why some vaccines to saliva can worsen outcome (Reagan et al., 2012). Our findings also have implications for the development of new “naturalized” models of virus infection that make use of mosquito saliva enhancement. Many viruses, including important human pathogens such as chikungunya virus (Teo et al., 2012), dengue virus (Zompi and Harris, 2012), and bunyaviruses (Bridgen et al., 2001), struggle to replicate and disseminate within wild-type mice after infection by needle into resting skin. Accordingly, immunosuppressed mice are often used as an alternative, which precludes the experimental study of many aspects of host immune responses to these infections. Building on the work of other important studies (Conway et al., 2014, Cox et al., 2012, McCracken et al., 2014, Moser et al., 2015, Schneider et al., 2006, Styer et al., 2011), we suggest that the inclusion of mosquito bites, or their saliva, might sufficiently enhance viral replication to enable the study of these infections in wild-type mice.

In conclusion, we highlight an important aspect of host innate immunity at mosquito bite sites. These findings not only define the mosquito bite site as a putative target for post-exposure prophylactic intervention, but also pave the way for the development of in vivo models that better recapitulate an important aspect of mosquito-borne virus infection.

## Experimental Procedures

Detailed methodology is described in the Supplemental Information.

### Cell Culture, Viruses, and Mice

*Aedes* mosquito cells and BHK cells were cultured using established protocols (Rodriguez-Andres et al., 2012). Details of reporter viruses can be obtained from the authors. The pCMV-SFV4 backbone for production of SFV4 has been previously described (Rodriguez-Andres et al., 2012, Ulper et al., 2008). Plasmids containing cDNAs of SFV were electroporated into BHK cells to generate infectious virus and then passaged once in mosquito cells. Titration of virus stocks and quantification of viraemia in vivo were performed via plaque assays (Rodriguez-Andres et al., 2012). Wild-type BUNV was generated as previously described (Bridgen et al., 2001). All mice were maintained under specific-pathogen-free conditions at the Central Research Facility, University of Glasgow. All mice were housed in accordance with local and Home Office regulations.

### Mosquito Biting of Mice and Virus Infection

Anesthetized mice were positioned to expose a defined area of the left foot to a cage of *A. aegypti* mosquitoes. The remainder of the mouse body surface was protected. Mice were monitored during mosquito biting and a maximum of five mosquitos were allowed to engorge from the area exposed. Allowing more than one mosquito to probe/bite the available skin surface ensured that most of the exposed skin surface was subjected to probing/bites. Immediately after completion of mosquito biting, the bitten skin was injected with 1 μL of virus using a Hamilton Syringe (Hamilton). This approach enabled the effect of bites on concurrent virus infection to be quantifiably compared to virus infection alone in the absence of a bite.

### RNA Extraction, Gene Expression Analysis, Flow Cytometery, and Immunohistochemistry

RNA was extracted using PureLink Plus columns and converted to cDNA using the High Capacity RNA-to-cDNA kit (Life Technologies). qPCR analysis was undertaken using SYBR-green PerfeCTa (Quanta) and TLDAs were used as per manufacturer’s instructions on a 7900HT Real time machine (Applied Biosystems). ELISAs were undertaken using Duoset kits (R&D Systems). For flow cytometry, skin was enzymatically digested to release cells and stained using a subset of antibodies. Cells were stained with Fixable Viability Dye eFluor780 (eBioscience), fixed in 4% methanol-free paraformaldehyde (Thermo Scientific) or Cytofix/Cytoperm (BD), and analyzed on a MACSQuant Analyzer 10 (Miltenyi). For cell sorting, cells were labeled with CD11b beads and sorted on magnetized columns (Miltenyi). For immunohistochemistry, skin was fixed before freezing in embedding medium and sectioned. Sections were blocked in Tris-Saline-Tween (TBS)/5% fish gelatin (Sigma-Aldrich) incubated with a primary antibody against Lyve-1 and the secondary chicken anti-goat IgG Alexa Fluor 647-conjugated antibody (Life Technologies).

### Statistical Analysis

Data were analyzed using the non-parametric-based tests Mann-Whitney or Kruskal-Wallis test with Dunn’s multiple comparison. All column plots show the median value ± interquartile range with ^∗^p < 0.05, ^∗∗^p < 0.01, ^∗∗∗^p < 0.001, ^∗∗∗∗^p < 0.0001, ns = not significant. Wherever possible, preliminary experiments were performed to determine requirements for sample size, taking into account the available resources and ethical use of animals. Animals (gender and age matched) were assigned randomly to experimental groups. For plaque assays, samples were coded and analyzed blind by a separate investigator. All results shown are representative of either two or three experiments and where possible incorporate a variety of techniques and approaches. Importantly, biological replicates were excluded from analysis if s.c. or i.d. injection of virus inadvertently punctured a blood vessel.

## Author Contributions

Conceptualization, C.S.M., J.K.F., and G.J.G.; Methodology, C.S.M., E.S., A.K., J.K.F., and G.J.G.; Investigation, M.P., S.R.B., E.P., E.S., and C.S.M.; Resources, E.S., A.K., E.P., A.M., and G.J.G.; Original draft, M.P. and C.S.M.; Review & Editing, all authors; Funding acquisition, C.S.M.

## Supplemental Information

Document S1. Figures S1–S7 and Supplemental Experimental Procedures

Document S2. Article plus Supplemental Information
