# Supplementary material for: Host Inflammatory Response to Mosquito Bites Enhances the Severity of Arbovirus Infection
Source: Immunity. 2016 Jun 21;44(6):1455–69. doi: 10.1016/j.immuni.2016.06.002 (PMC4920956; doi:10.1016/j.immuni.2016.06.002)
Supplement: Document S1. Figures S1–S7 and Supplemental Experimental Procedures [file mmc1.pdf]

Immunity, Volume *44*

Supplemental Information

Host Inflammatory Response to Mosquito Bites

Enhances the Severity of Arbovirus Infection

Marieke Pingen, Steven R. Bryden, Emilie Pondeville, Esther Schnettler, Alain Kohl, Andres Merits, John K. Fazakerley, Gerard J. Graham, and Clive S. McKimmie

Figure S1. In the absence of mosquito bites, SFV4 rapidly disseminates from skin inoculation sites to establish a peak viremia by 24 hours and activates the induction of type I IFNs in the draining popliteal lymph node (refers to Figure 1).

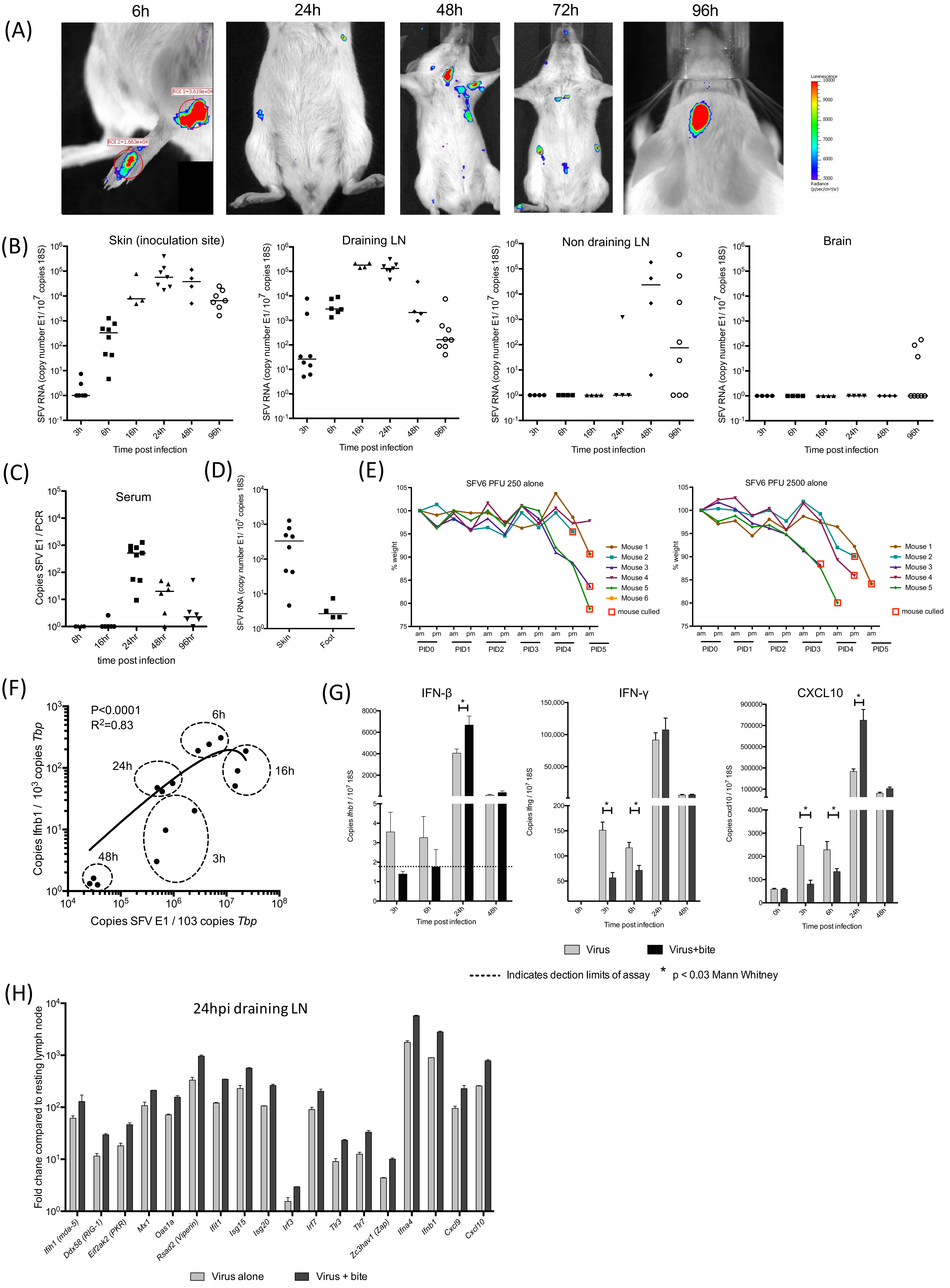

Figure S2. Fold change of innate immune transcripts in skin following mosquito bite or SFV4 infection alone (refers to Figure 2).

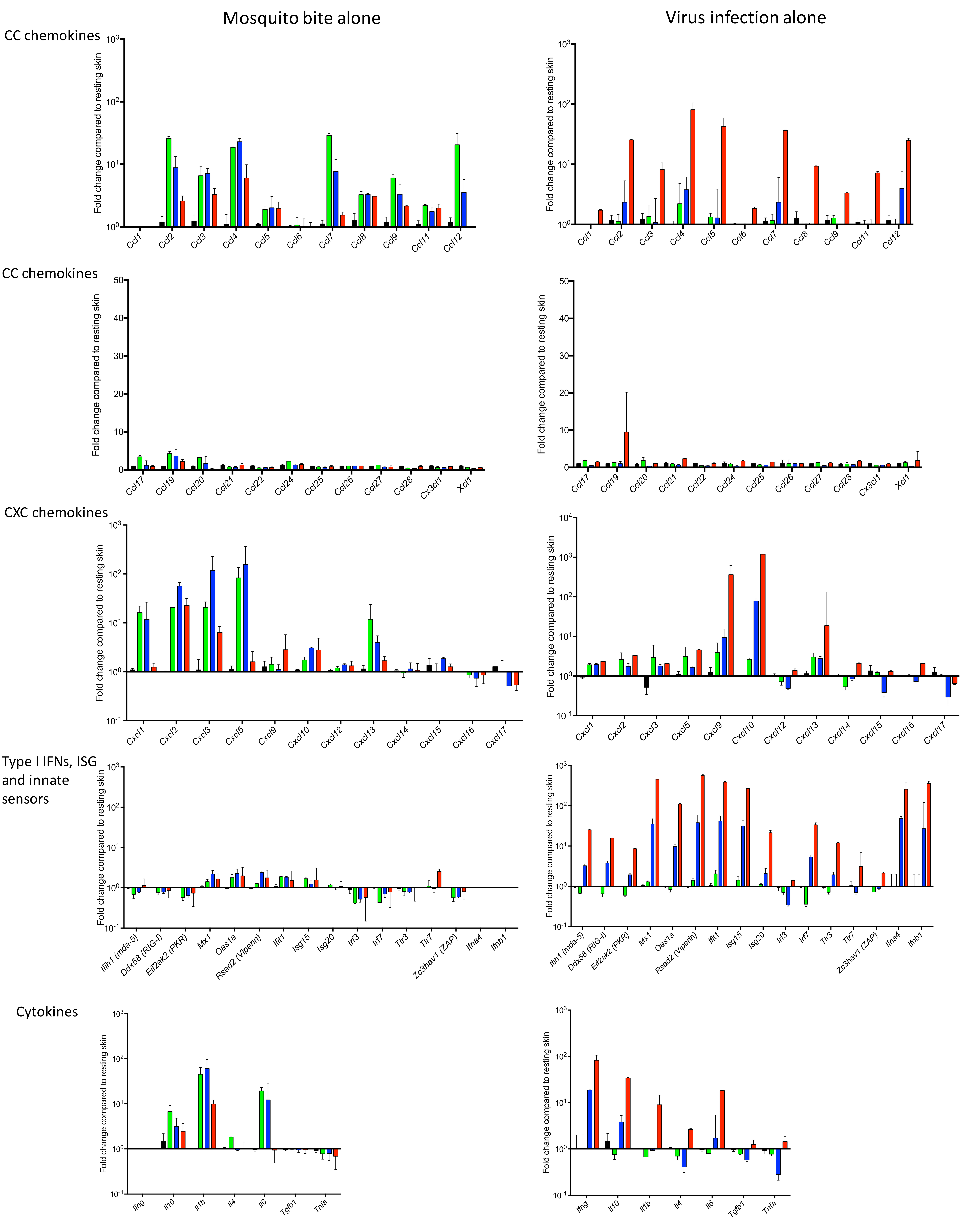

Figure S3. Mosquito bite-experienced mice exhibit similar gene expression changes at 6h following a new mosquito bite, compared to bite-naïve mice bitten for the first time (refers to figure 2).

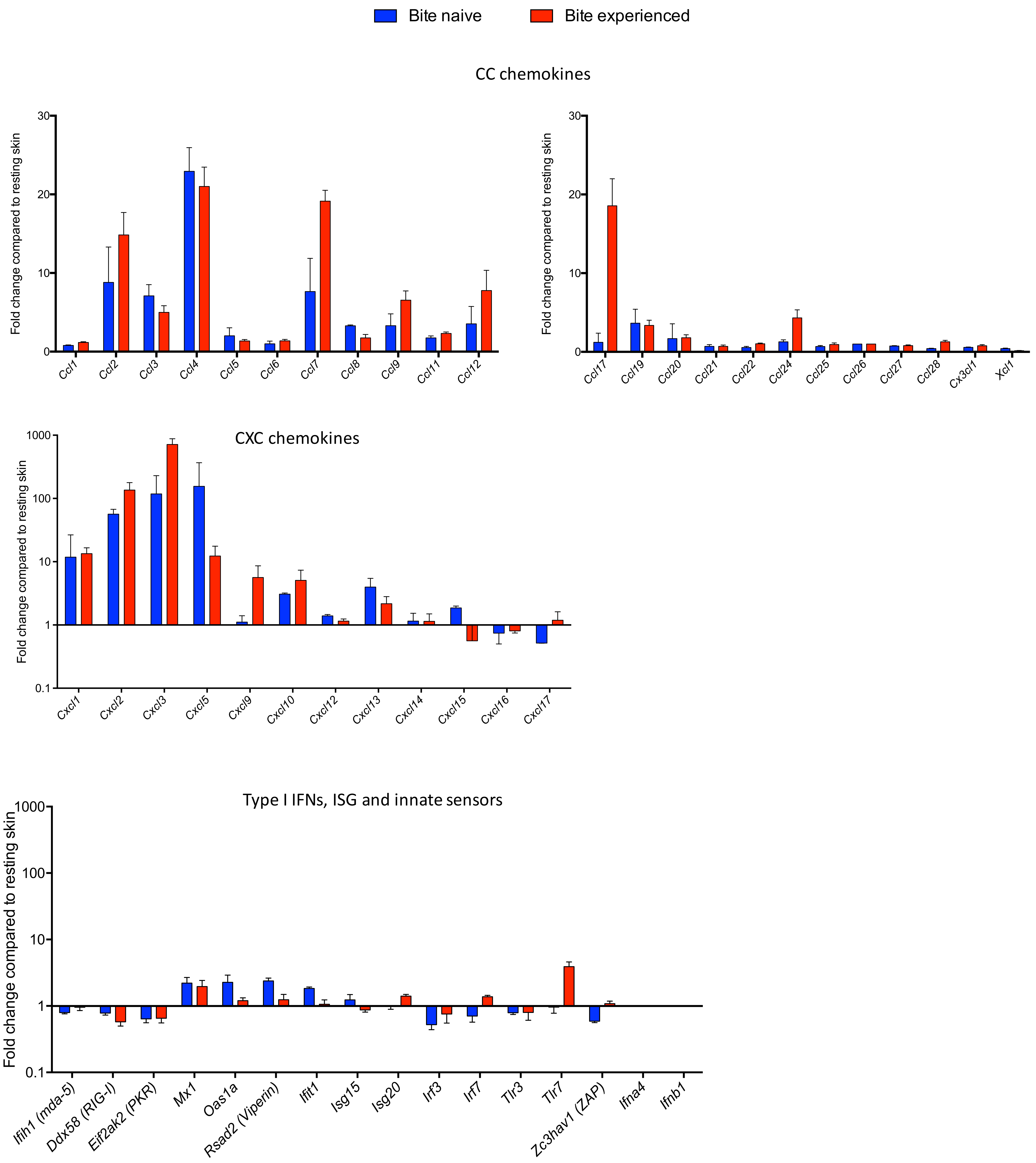

Figure S4. Cutaneous innate immune responses to mosquito bites and virus infection (refers to Figure 2)

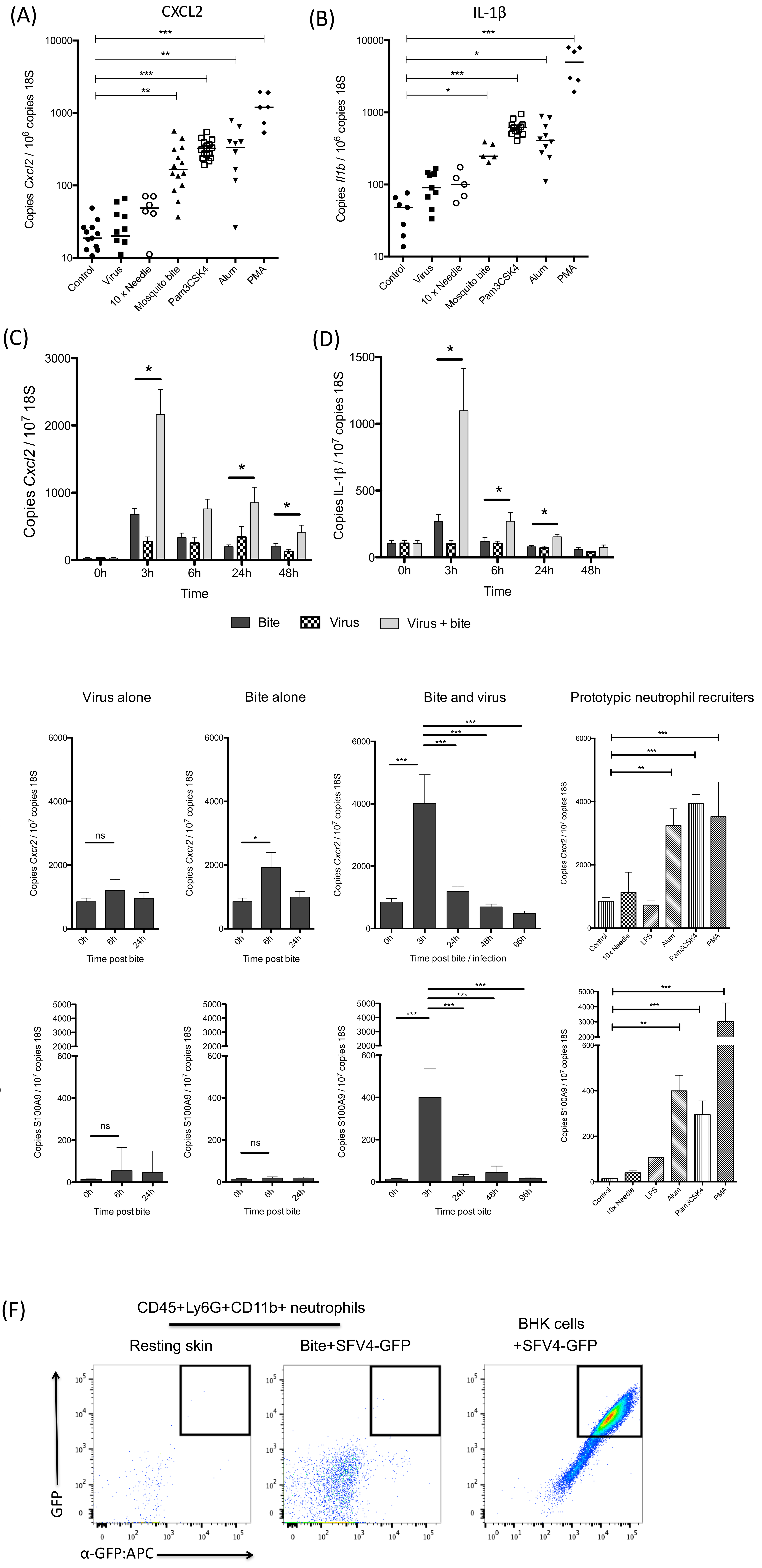

Figure S5. Neutrophils were depleted *in vivo* using the Ly6G antibody IA8 (refers to figure 4).

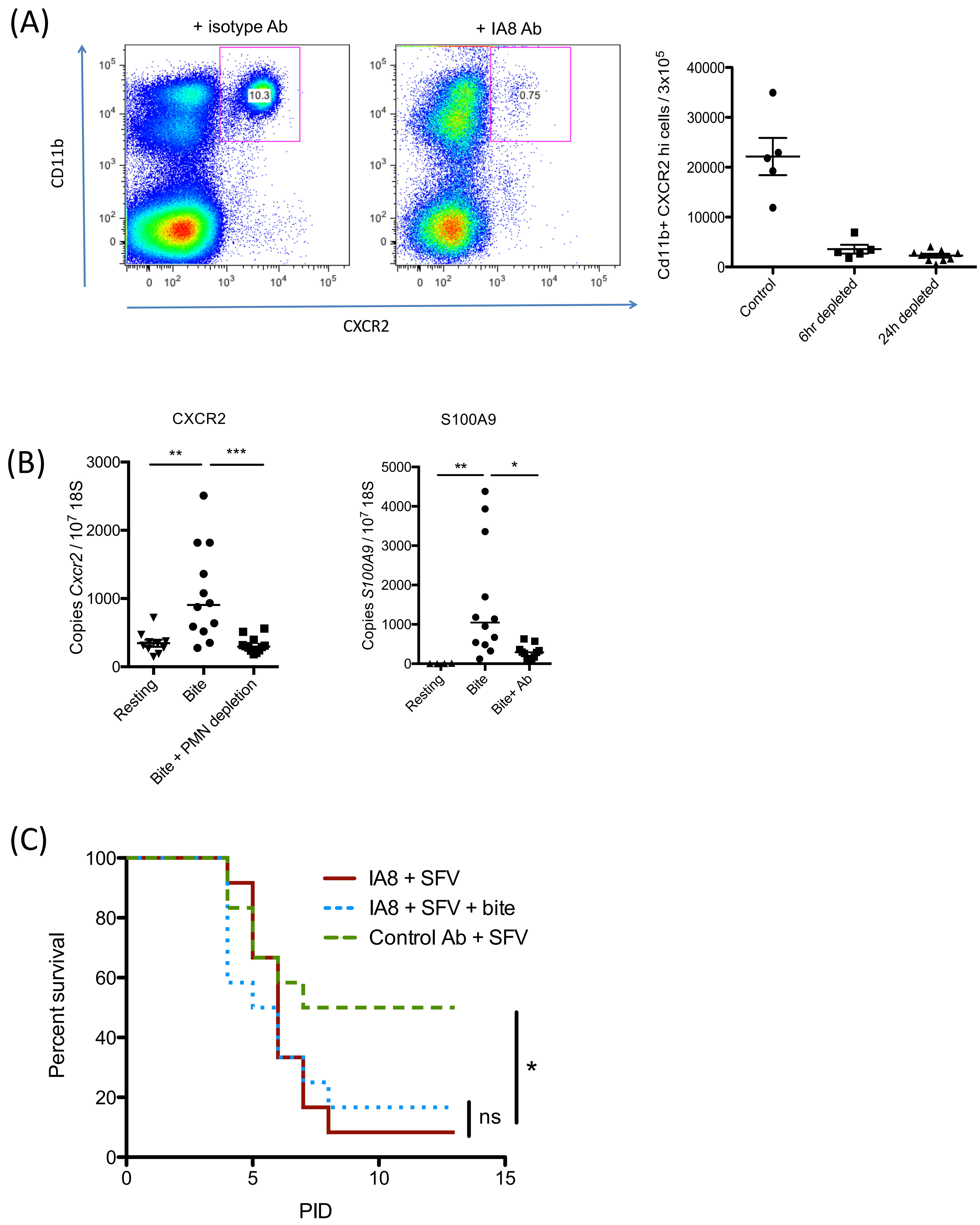

Figure S6. Pro-inflammatory agents enhance infection despite a pronounced type I IFN response (refers to figure 5).

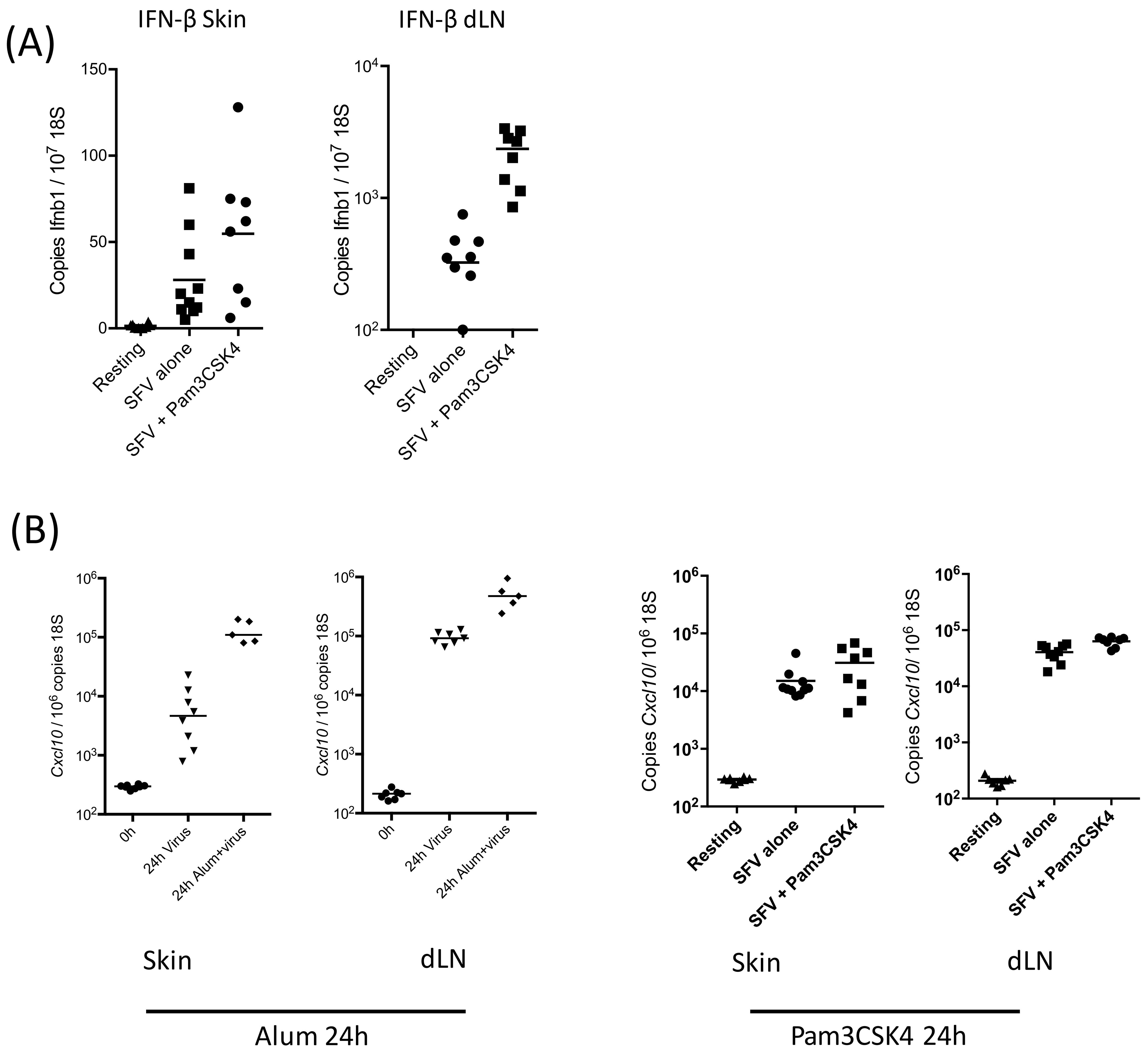

Figure S7. Leukocyte populations in the skin of mosquito bitten SFV-infected mice (refers to figure 6)

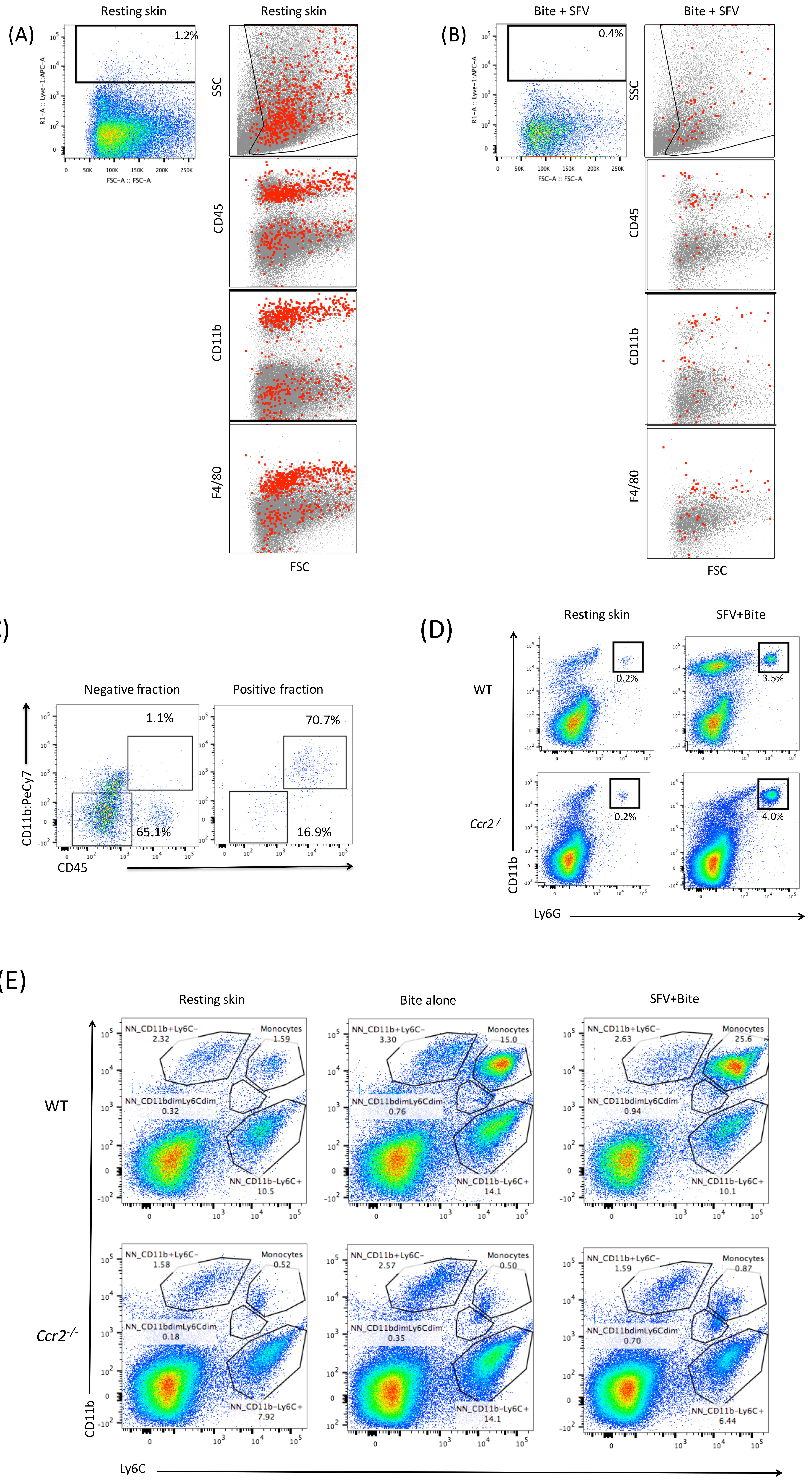

Supplementary information.

Supplementary figure legends.

Figure S1

In the absence of mosquito bites SFV4 rapidly disseminates from skin inoculation sites to establish a peak viremia by 24 hours and activates the induction of type I IFNs in the draining popliteal lymph node (refers to Figure 1).

(A) Mice were infected with SFV4(3H)-*RLuc* into the upper skin of the left foot. Following infection, RLuc activity was determined by intravital imaging (IVIS) (n=4). Injection of luciferase reagent in the tail vein resulted in a localized background signal and has been cropped from the image or masked using a black box.

(B-D) SFV4 replicates at local cutaneous inoculation sites and quickly disseminates to infect draining lymph nodes, followed by occasional spread to non-draining lymph nodes and the brain. Mice were infected with 10^3 PFU of SFV4 into the upper skin of the left foot and viral RNAs levels determined by qPCR. (B) The skin inoculation site, draining popliteal LN, non-draining contralateral popliteal LN and brain were dissected using fine biopsy tools and SFV E1 RNA and 18S quantified by qPCR. Those tissues positive for Renilla luciferase activity (Figure 1A) were also positive for SFV E1 RNA. (C) Serum was collected, cell-free RNA extracted and copies of the SFV RNA determined by qPCR. (D) SFV E1 RNAs were quantified for skin inoculation site and underlying tissue of the foot at 6h post infection, demonstrating that the majority of the virus inoculum had infected cutaneous tissue.

(E) Mice were infected with either 250 or 2500 PFU of virulent SFV6 s.c into skin of the foot. Weight of infected mice were measured twice a day until either the end of the experiment or until they reached clinically defined end points (denoted by a red square).

(F-H) Type I IFN responses in the infected draining popliteal LN were proportional to viral RNA levels and were delayed in mice subjected to mosquito bites. (F) SFV E1 RNAs correlated with IFN-β transcripts in the draining popliteal LN in a time dependent manner. Spearman's correlation was used to generate P and R^2 values. Gene transcripts were normalised to Tata Bind protein (TBP). (G) Mice were infected with SFV4 +/- mosquito bite and transcripts quantified by QPCR in draining popliteal LNs (H) Mice were similarly infected with SFV4 either in the presence (light grey) or absence of a mosquito bite (dark grey) and transcripts for type I IFNs and prototypic ISGs in the draining popliteal LN assayed by Taqman low density array at 24h post infection. Transcript numbers were normalized using 18S.

Figure S2

Fold change of innate immune transcripts in skin following a mosquito bite or SFV4 infection alone (refers to Figure 2).

Mice were subjected to mosquito bites alone or virus infection alone and transcript fold change quantified by Taqman low-density array. Fold changes was calculated by comparison to resting skin and grouped based on functional classes of genes; CC chemokines, CXC chemokines, cytokines and type I IFNs, ISGs and innate immune sensors. Bars show median value +/- interquartile range.

Figure S3

Mosquito bite-experienced mice exhibit similar gene expression changes 6h following a new mosquito bite, compared to bite-naïve mice bitten for the first time (refers to figure 2).

Mice were either left unbitten or bitten with mosquitoes, once a week for 4 weekd. Both roups of mice were then exposed to biting mosquitoes and gene transcript fold change determined by Taqman low-density array at 6 hours post bite. Bars represent median fold

change of bite-naïve mice (blue) and bite-experienced mice (red) compared to resting unbitten mice. Bars show median value +/- interquartile range.

Figure S4

Cutaneous innate immune responses to bites and virus infection (refers to figure 2).

(A,B) Copy numbers of transcripts for CXCL2 (A) and IL-1β (B) were determined by qPCR in mosquito bitten skin and in skin stimulated with known inducers of cutaneous neutrophil influx at 6h.

(C,D) CXCL2 (n=6) and IL-1β transcripts levels were determined by qPCR in skin following either mosquito bite alone, virus infection alone, or the combination of both.

(E) To better determine the kinetics of neutrophil entry and tissue residency we employed a highly sensitive qPCR-based strategy to quantify the expression of neutrophil markers, CXCR2 and S100A9. This analysis showed a robust, but highly transient increase in both CXCR2 and S100A9 expression, peaking at 3h post bite/infection, and which was comparable to the increases seen in skin following application of Alum, Pam3CSK4 or TPA at 6h. Bars show mean +/- SD. Statistical testing was undertaken using 1-way ANOVA and Tukey's multiple comparison post tests.

(F) To determine if virus could infect neutrophils recruited to the bite site, mice were bitten then infected with $5x10^5$ PFU of SFV4(Xho)-EGFP. Skin cells positive for $CD45^{hi}$, $CD11b^{hi}$ and $Ly6G^{hi}$ expression were gated by FACS and their positivity for SFV-GFP determined by measuring both GFP signal and also the signal generated by an anti-GFP:APC antibody. The intensity of GFP signal and α-GFP:APC staining was compared to that of infected BHK fibroblasts and suggested that neutrophils were not infected with SFV.

Figure S5

Neutrophils were depleted in vivo using the Ly6G antibody IA8 (refers to figure 4).

(A,B) The IA8 antibody was effective at depleting neutrophils from the systemic circulation and reduced the abundance of neutrophil specific markers in mosquito bitten skin. Neutrophils were depleted in vivo using the anti-Ly6G IA8 antibody and the number of circuiting and skin resident neutrophils compared to numbers in mice treated with a non-depleting isotype control antibody. (A) Circulating neutrophils ($CD11b^{hi}$ $CXCR2^{hi}$) in the plasma were quantified. (B) PMN infiltration into mosquito bitten skin of IA8-treated mice was analyzed by measuring the increase of neutrophil specific gene transcripts CXCR2 and S1A009 by QPCR at 3h post bite. Treatment with IA8 reduced the number of CXCR2 transcripts. Data points represent the values generated by individual mice.

(C) Depletion of neutrophils reduces survival to SFV6 infection irrespective of the presence of mosquito bites. Mice were depleted of neutrophils using IA8 and then infected with virulent SFV6, either in absence (red line) or presence of a mosquito bite (blue line). Non-bitten mice that had treated with the non PMN-depleting control 2A3 antibody, were also infected for comparison (green line), n=15.

Figure S6

Pro-inflammatory agents enhance infection despite a pronounced type I IFN response (refers to figure 5).

(A,B) Pam3CSK4 or Alum, when injected s.c. into the skin at the time of virus infection, does not suppress induction of anti-viral type I IFN or ISG induction by virus at 24hpi. Copy numbers of gene transcripts for IFN-β (A) and the prototypic ISG, CXCL10 (B), were determined by qPCR.

Figure S7

Leukocyte populations in the skin of mosquito bitten SFV-infected mice (refers to figure 7)

(A,B) The majority of lyve1+ cells analyzed by FACS were dermal macrophages. Cutaneous cells were analyzed by FACS following digestion of resting skin (A) and virus infected skin at 24h (B). Back-gating of all Lyve1^{hi} cells (red dots) reveals that the majority are CD45+CD11b+F4/80+ macrophages, and that these are depleted at 24 hpi with SFV.

(C) Success of our CD11b+ sorting of cutaneous cells. Mosquito bitten, virus infected skin was digested to release a single-cell solution, cells labelled with magnetic beads to CD11b and sorted on columns. The resulting negative and positive fractions were analyzed for FACS to characterize their purity.

(D, E) Leukocyte influx into skin of CCR2 null mice. (D) Neutrophil influx in the skin of CCR2 null mice was compared to that of wild type mice. Following SFV4 infection at mosquito bite sites, neutrophil influx at 3 hpi was similar in both wild type and CCR2 deficient mice. (E) Live cutaneous cells were gated to remove Ly6G^{hi} neutrophils and then analyzed for CD11b and Ly6C expression. Monocyte population was defined as CD11b^{hi}Ly6C^{hi}. Bites and SFV infection resulted in significant influx of monocytes in WT but not CCR2 null mice at 18hpi.

Extended Experimental Procedures

Cell culture, viruses and mice.

Ae. aegypti-derived AAG2 and *Ae. albopictus*-derived C6/36 mosquito cells were grown at 28°C in L-15 medium with 10% fetal calf serum, 10% tryptose phosphate broth, 100 units/ml penicillin and 0.1 mg/ml streptomycin. BHK-21 cells were grown in Glasgow minimum essential medium (GMEM) with 5% fetal calf serum, 10% tryptose phosphate broth, 100 units/ml penicillin and 0.1 mg/ml streptomycin at 37°C in a 5% CO_2 atmosphere. Two SFV strains were used: SFV4 is an avirulent strain that rarely triggers clinical disease, while highly SFV6 is highly virulent and causes lethal encephalitis within a week (Ferguson et al., 2015; Michlmayr et al., 2014). SFV4 was used to study viral dissemination and the local immune response, while SFV6 was used to study morbidity. Details of reporter viruses can be obtained from the authors. The pCMV-SFV4 backbone for production of SFV4 has been previously described (Ulper et al., 2008). The EGFP marker gene was inserted into the C-terminal region of nsP3 via a XhoI site naturally occurring in the genomic sequence (leading to expression of nsP3 fused to EGFP), while *Renilla* luciferase (RLuc) was inserted between duplicated nsP2 cleavage sites at the nsP3/4 junction as a cleavable reporter, using strategies previously described (Rodriguez-Andres et al., 2012). Plasmids containing the genomic sequence of wild type SFV and recombinant clones containing genetic markers (strains SFV4 and SFV6) were electroporated into BHK cells to generate infectious virus. All viruses were then passaged once in either *Aedes* AAG2 or C6/36 cells, purified from supernatant, and resuspended in Tris-NaCl-EDTA buffer as described, and titrated in BHK-21 cells by plaque assay (see below). Viruses were diluted in PBSA (PBS with 0.75% bovine serum albumin) to $1x10^7$ plaque forming units (PFU)/ml for injection into mice. Working stocks of wild-type BUNV were grown in BHK-21 cells at the lower temperature of 33°C, to decrease the frequency of defective viral particles, centrifuged to remove cell debris and virus titers were determined by plaque assays on BHK-21 cells (Bridgen et al., 2001).

Titration of virus stocks and quantification of viraemia *in vivo* were performed using plaque assays. Virus stocks, plasma or serum (as indicated in the Figure legends) were serially diluted in 0.75% PBSA and used to infect 70-80% confluent BHK-21 cells for 1 hour, and then overlaid with a 1:1 mixture of 1.2% Avicel RC-591 NF (kindly provided by FMC BioPolymer) in dH_2O and 2xMEM with 4% fetal calf serum, 200 units/ml penicillin and 0.2 mg/ml streptomycin. After 2 days incubation at 37°C/5% CO_2, cells were fixed using 10%

paraformaldehyde and stained with 0.1% toluidine blue. Viral titer was calculated based on number of plaques (Rodriguez-Andres et al., 2012).
7-week-old C57bl/6 and SCID mice were purchased from Charles River. CCR2 deficient mice were originally obtained from The Jackson Laboratory (stock number 004999) and bred in-house. *Il1r1*$^{-/-}$ mice were obtained from The Jackson Laboraory (B6.129S7-/l1r1^{tm1Imx}/J, stock number 003245). All mice were maintained under specific pathogen-free conditions at the Central Research Facility, University of Glasgow. All mice were housed in conventional (C57bl/6, *Ccr2*$^{-/-}$ deficient mice) or filter-topped cages (SCID and *Il1r1*$^{-/-}$ mice) and maintained in accordance with local and UK Home Office regulations.

Mosquito biting of mice and virus infection

Before mosquito biting, mice were anesthetized with an i.p. injection of KETASET (ketamine hydrochloride). To ensure mosquitoes bit a defined/restricted area of skin, the upper side of the foot was placed onto a mosquito cage containing 100 female *Ae. aegypti* mosquitoes (locally bred colony) and secured into place using tape. Up to 4 mice were similarly prepared per mosquito cage. The remainder of the mouse body surfaces, including toes and lower leg, were protected from probing mosquitoes by a textile and aluminium foil barrier. Mice were carefully monitored during mosquito biting, and a maximum of 5 mosquitos were allowed to engorge from the exposed area. Immediately after completion of mosquito biting (<5 minutes), the bitten skin was injected with a defined dose of virus in a small 1 μl volume. Allowing more than one mosquito to probe/bite the available skin surface ensured that most of the exposed skin was subjected to probing/bites. Thus, by clearly defining the number of mosquito bites to a restricted area of skin and by injecting a known titre of virus inoculum into this defined cutaneous site, it was possible to guarantee that virus was injected into either; i) mosquito bitten, or ii) resting unaffected skin. This approach enabled the effect of bites on concurrent virus infection to be quantifiably compared to virus infection alone in the absence of a bite. This comparison is not possible if using infected mosquitoes to infect mice; the inoculum supplied by biting mosquitoes was too variable and unpredictable to allow effective comparisons to needle inoculated virus in the absence of bites.
For virus infection, both in the absence or presence of mosquito bites, mice were anesthetized (KETASET injection (i.p.), or isoflurane by inhalation). 1 μl of virus in PBSA was injected into the skin of the upper side of the foot using either; 10^3 or 10^4 PFU SFV4, $2.5x10^2$ PFU SFV6 or $2.5x10^4$ PFU BUNV. Injections were undertaken using a Hamilton Syringe and custom-made point 4 style 33 gauge needles (Hamilton, Switzerland).

Mosquito saliva

Mosquitoes were starved for one day prior to salivation. Using a microscope, legs and wings of female mosquitoes were carefully dissected after which the proboscis was placed in a 10 μl tip containing 1 μl non-drying immersion oil (Cargille). Subsequently, mosquitoes were placed at 28°C and allowed to salivate for >1 hour. Successful salivation was confirmed by microscopy; saliva was visible as bubbles in the oil. The tips were briefly centrifuged to collect and combine saliva droplets. The saliva was further pooled by centrifugation of the oil-saliva mixture for 15 minutes, 1000xg at 4°C, after which excess oil was removed with the aid of a dissecting scope. Saliva was stored at -80°C until use. To investigate the influence of saliva on viral replication, 1ul 0.75% PBSA containing 10^4 PFU SFV4 with or without the saliva of 5 mosquitoes was injected in resting or bitten skin as described above.

Measurement of RLuc activity and tissue fluid accumulation in vivo

To monitor viral replication *in vivo*, mice were infected with SFV4 encoding RLuc as a non-structural protein. Virus encoded RLuc activity was assessed using RediJect Coelenterazine h (Perkin Elmer) and an Intravital Imaging System (IVIS Spectrum; Caliper Life Sciences) as per manufacturers instructions. Briefly, mice were anesthetized using isoflurane-inhalation and injected intravenously, in the tail vein, with 15 μg of Coelenterazine h before RLuc activity measurement for up to ten minutes post injection. Injection of Coelenterazine h resulted in substantial background signal at the site of intravenous injection in the tail. Accordingly, this injection site artifact was removed from images to prevent confusion with virus-derived signal elsewhere in the body.

To determine the extent of fluid accumulation and vascular leakage at mosquito bite sites, mice were i.p. injected with Evans blue dye before infection/bite. Level of fluid accumulation at infection/bite site at 3 hours post challenge was determined by colorimetric measurement (620 nm) of tissue-free dye concentration after soaking samples in formamide for 24 hours.

Depletion of neutrophils

To deplete neutrophils, mice were injected with antibodies that bind to Ly6G as per manufacturer's instructions (BioXcell). Briefly, mice were injected i.p. with 200μl of IA8 antibody or control antibody (2A3) at 4 days and 1 day before infection, which specifically and effectively depletes $Ly6G^+$ cells (Jamieson et al., 2012). Successful neutrophil depletion

was confirmed by flow cytometry analysis of peripheral blood using neutrophil markers $CD11b^{hi}$ and $CXCR2^{hi}$ and qPCR analysis of CXCR2 transcripts in skin (Figure S5).

RNA extraction and gene expression analysis

RNA was extracted using PureLink Plus columns and DNA digested on column as per manufacturer's instruction (Life technologies). Briefly, tissue samples were homogenised in TRIzol (Life Technologies) using a TissueLyser LT with 7mm metal beads (Qiagen), followed by purification using PureLink columns with DNase digestion (Life Technologies). Up to 2 μg RNA was converted to cDNA using the High Capacity RNA-to-cDNA kit (Life technologies). Gene expression analysis was undertaken using custom designed SYBR-green based qPCR assays using PerfeCTa (Quanta). Because of the expression strategy of alphaviruses and the nature of their positive-sense RNA genomes, the qPCR assay for SFV E1 measures the sum value of both genome and subgenomic RNA used for E1 gene expression. A selection of representative samples was additionally analysed using Taqman Low Density Arrays (TLDA). For TLDA analysis, the cDNA generated from the equivalent of 1μg of total RNA was loaded into a custom TLDA plate and samples assayed as per manufacturer's instructions (Applied Biosystems). For serums samples cell-free virus RNA was extracted and copies of the SFV RNA determined by qPCR. Because there is no housekeeping gene to reference, a standard volume (100ul) of serum was used, and 1/60 of the resulting cDNA loaded into each qPCR. All SYBR green and Taqman assays were undertaken on a 7900HT Real time machine (Applied Biosystems). Primer sequences are available upon request.

ELISAs were undertaken using Duoset kits (R and D systems). Briefly, tissues samples were immediately lysed in T-PER lysis solution in the presence of protease inhibitors (Roche) by shaking at 50Hz with a 7mm steel bead (Qiagen) for 10 minutes in the TissueLyser LT. Supernatants were clarified by centrifugation at 16,000g for 15 minutes at $4^{o}C$. Supernatants were diluted 1:2 with ELISA diluent and analysed using DuoSet as per manufacturers instructions (R&D systems).

Survival curves

After infection with either $2.5x10^{2}$ PFU SFV6 or 10^{5} PFU SFV L10, mice were monitored several times a day for development of clinical signs. Mice were culled when they reached clinically defined end-points of disease, as previously described (Fazakerley, 2002; Michlmayr et al., 2014).

Flow cytometery, magnetic cell separation, immunohistochemistry and histology

For flow cytometry, bitten skin of the foot or back was enzymatically digested to release cells using 1 mg/ml collagenase D (Roche), 0.5 mg/ml dispase (Roche) and 0.1 mg/ml DNaseI (Invitrogen) in Hanks' balanced salt solution (Sigma) at 37°C, 1300 RPM. Cells were stained using a subset of the following antibodies: CD45 (30-F11), CXCR2 (TG11), Ly6G (IA8), APC or PerCP streptavidin, GFP:APC (FM264G) (all Biolegend), CD11b (M1/70), F4/80 (BM8), Ly6G (RB6-8C5), Ly6C (HK1.4), Lyve-1 (ALY7) and pro-IL-1β (NJTEN3) or matching IgG1 K isotype control (P3.6.2.8.1) (all eBioscience). Furthermore, cells were stained with Fixable Viability Due eFluor780 (eBioscience) and fixed in 4% methanol-free paraformaldehyde (Thermo Scientific) or Cytofix/Cytoperm (BD) before analysis using a MACSQuant (Milteny). Analysis was performed using Flowjo version 8.8.7 or 10.0.7 (TreeStar). Cells were first gated to exclude doublets and dead cells, and then gated for CD45 or CD11b to remove stromal cells.

For magnetic cell separation, virus infected skin was enzymatically digested as described above. Cells were sorted based on CD11b expression as per manufacturers instructions (CD11b MicroBeads, clone M1/70, Miltenyi). Briefly, cells were labelled using 10 µl CD11b microbeads after which the positive and negative fraction were separated on MS columns. The positive and negative fraction were subsequently cultured in RPMI media 1640 (Invitrogen) with 10% fetal calf serum, 100 units/ml penicillin, 0.1 mg/ml streptomycin, 0.05 mg/ml gentamycin, 50 nM β-Mercaptoethanol and 2.5 mM HEPES. Virus release was measured in cell-free supernatant at various periods of culturing using plaque assays.

For immunohistochemistry, skin of the back was shaven before bite/infection. Skin was fixed for 24 hours in 4% methanol-free paraformaldehyde (Thermo Scientific) before freezing in OCT embedding medium (Tissue Tech) on dry ice. Sections of 8-10 µm were cut on a Shandon Cryotome (Thermo Scientific) and mounted onto Colorfrost Plus microscope slides (Thermo Scientific). After washing with PBS, a hydrophobic barrier was applied using an ImmEdge pen (Vector Laboratories) around the sections. Subsequently, sections were blocked in Tris-Saline-Tween (TBS)/5% fish gelatin (Sigma-Aldrich) for one hour, washed with TBS, and incubated overnight at 4°C with a primary antibody against Lyve-1 (affinity purified polyclonal goat IgG, R&D systems) or inflammatory macrophages (ER-HR3 conjugated to APC, BioLegend) in TBS/2% fish gelatin. Lyve-1 treated slides were washed three times in TBS before addition of the secondary chicken anti-goat IgG Alexa Fluor 647 conjugated antibody (Life Technologies) for 1 hour at 4°C. Sections were washed twice in TBS before mounting in Vectashield mounting medium with DAPI (Vector laboratories).

For histology, skin was fixed overnight in neutral buffered formalin (Leica) before progressive dehydration through increasing concentrations of ethanol to xylene (tissue processor Shandon Citadel 1000, Thermo Scientific). Skin samples were embedded in paraffin wax and 10 μm sections were cut and mounted onto Superfrost slides (Fisher Scientific). Haematoxylin and eosin staining was performed according to standard protocol. Sections were rehydrated in water via decreasing alcohol concentrations before a 7-minute stain in Haematoxylin Z (Cell path). Sections were rinsed in water, 1% acid alcohol, water, and immerged in Scott's Tap Water Substitute for 2 minutes. Sections were washed in water before a 4-minute stain in Putts Eosin (Cell Path), and washed in running water, 70% ethanol, 100% ethanol (2x), xylene (3x) before mounting using dibutyl phthalate xylene and visualization on a light microscope.

Statistical analysis

Data were analyzed using Prism Version 5 software. *In vivo* derived data from virus-infected mice was not normally distributed and was accordingly analyzed using the non-parametric based tests Mann-Whitney, Kolmogorov-Smirnov, or Kruskal-Wallis test with Dunn's multiple comparison. Unless otherwise stated all column plots have statistical significance indicated; *p<0.05, **p<0.01, ***p<0.001, ****p<0.0001, ns=not significant. Unless otherwise stated all column plots show the median value +/- interquartile range. Wherever possible, preliminary experiments were performed to determine requirements for sample size, taking into account the available resources and ethical use of animals. Animals (gender and age matched) were assigned randomly to experimental groups. For plaque assays, samples were coded and analyzed blind by a separate investigator. TLDA gene expression data was subjected hierarchal clustering in GeneSpring (Agilent) to generate heat maps. All survival curves were analyzed using the logrank (Mantel Cox) test. Correlation was calculated using Pearson. For qPCR data, each dot plotted represents the median of 4 technical replicates of one biological replicate. All results shown are representative of either two or three experiments. Importantly, biological replicates were excluded from analysis if s.c. or i.d. injection of virus inadvertently punctured a blood vessel.
