# Supplementary material for: Host Inflammatory Response to Mosquito Bites Enhances the Severity of Arbovirus Infection
Source: Immunity. 2016 Jun 21;44(6):1455–69. doi: 10.1016/j.immuni.2016.06.002 (PMC4920956; doi:10.1016/j.immuni.2016.06.002)
Supplement: Document S2. Article plus Supplemental Information [file mmc2.pdf]

Article

Immunity

Host Inflammatory Response to Mosquito Bites Enhances the Severity of Arbovirus Infection

Graphical Abstract

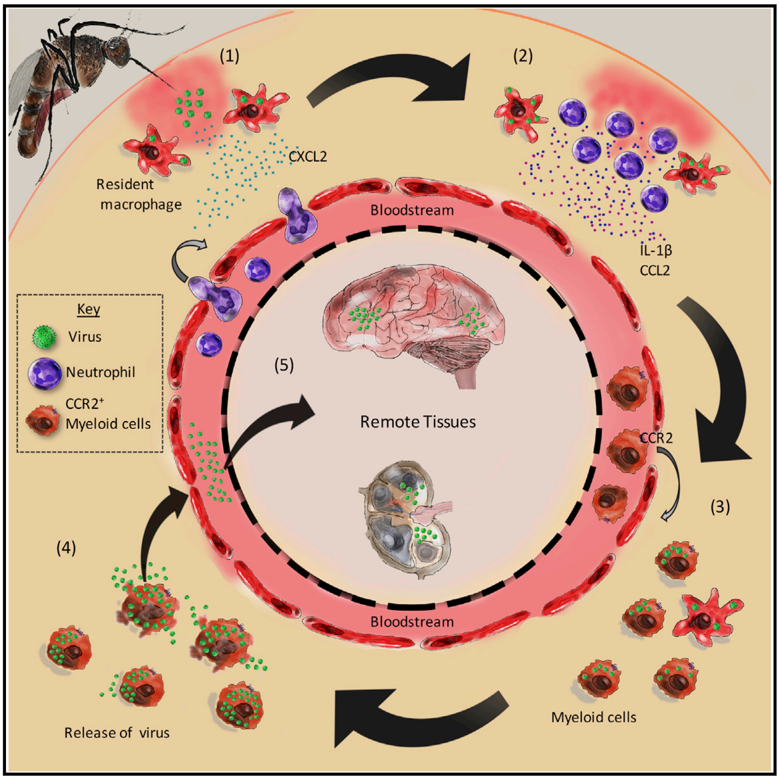

Highlights

- Mosquito bites enhance virus replication and dissemination and increase host mortality
- Neutrophil-driven inflammation retains virus in skin to drive macrophage recruitment
- Recruited and resident myeloid cells become infected and replicate virus
- Blocking leukocyte recruitment to bite site inhibits viral infection

Authors

Marieke Pingen, Steven R. Bryden, Emilie Pondeville, ..., John K. Fazakerley, Gerard J. Graham, Clive S. McKimmie

Correspondence

c.s.mckimmie@leeds.ac.uk

In Brief

The inoculation of viruses into mosquito bite sites is an important and common stage of arbovirus infections. McKimmie and colleagues show that inflammation at bite sites aids viral replication and dissemination in vivo, resulting in more severe infection. These findings define additional targets for post-exposure prophylactic intervention.

Pingen et al., 2016, Immunity *44*, 1455–1469
June 21, 2016 © 2016 The Author(s). Published by Elsevier Inc.
http://dx.doi.org/10.1016/j.immuni.2016.06.002

CellPress

Immunity
Article

Host Inflammatory Response to Mosquito Bites Enhances the Severity of Arbovirus Infection

Marieke Pingen,[1] Steven R. Bryden,[1,2] Emilie Pondeville,[3] Esther Schnettler,[3] Alain Kohl,[3] Andres Merits,[4] John K. Fazakerley,[5] Gerard J. Graham,[2] and Clive S. McKimmie[1,*]
[1]Virus Host Interaction Team, Section of Infection and Immunity, Leeds Institute of Cancer and Pathology, University of Leeds, Leeds LS9 7TF, UK
[2]Institute of Infection, Immunology and Inflammation, University of Glasgow, Glasgow G12 8TA, UK
[3]MRC-University of Glasgow Centre for Virus Research, Glasgow G61 1QH, UK
[4]Institute of Technology, University of Tartu, 50411 Tartu, Estonia
[5]The Pirbright Institute, Ash Road, Pirbright, Surrey GU24 0NF, UK
*Correspondence: c.s.mckimmie@leeds.ac.uk
http://dx.doi.org/10.1016/j.immuni.2016.06.002

SUMMARY

***Aedes aegypti* mosquitoes are responsible for transmitting many medically important viruses such as those that cause Zika and dengue. The inoculation of viruses into mosquito bite sites is an important and common stage of all mosquito-borne virus infections. We show, using Semliki Forest virus and Bunyamwera virus, that these viruses use this inflammatory niche to aid their replication and dissemination in vivo. Mosquito bites were characterized by an edema that retained virus at the inoculation site and an inflammatory influx of neutrophils that coordinated a localized innate immune program that inadvertently facilitated virus infection by encouraging the entry and infection of virus-permissive myeloid cells. Neutrophil depletion and therapeutic blockade of inflammasome activity suppressed inflammation and abrogated the ability of the bite to promote infection. This study identifies facets of mosquito bite inflammation that are important determinants of the subsequent systemic course and clinical outcome of virus infection.**

INTRODUCTION

The burden of mosquito-borne viral disease is profound. In recent years there has been a rapid increase in both the incidence and geographical range of such diseases, with spread to more temperate climates becoming more likely. Medically important viruses spread by arthropods (known as arboviruses) infect hundreds of millions of people each year (Bhatt et al., 2013; Weaver and Lecuit, 2015). This includes the chikungunya and Zika viruses that have recently triggered large-scale epidemics in the Americas (Burt et al., 2012; Gatherer and Kohl, 2016). The day-biting *Aedes* mosquitoes, and in particular *A. aegypti*, are the primary vectors. Arboviruses are an exceptionally large and diverse group of viruses (Elliott, 2014; Gould and Solomon, 2008; Powers et al., 2001). This heterogeneity, combined with the inability to accurately predict future arbovirus epidemics, makes developing and stockpiling specific drugs and vaccines very challenging.

All mosquito-borne viruses share a common attribute: their site of inoculation at mosquito bite sites. This aspect of their life cycle might provide a novel target for preventing diseases spread by this vector. In susceptible vertebrates, arbovirus replication in tissues results in a transient but very high level of infectious virus in the blood that is sufficient for a feeding arthropod to become infected. The high-level viraemia often induces a debilitating febrile illness and can result in the spread of virus to other tissues such as the brain, joints, and muscle. The early events of arbovirus infection are important for survival of the host, with a close relationship between early peripheral virus burden and mortality (Ryman and Klimstra, 2008). However, there remains a need to understand the determinants of early peripheral virus burden and what role cutaneous innate immune responses have in modulating viral replication and spread. It has previously been shown that mosquito bites enhance subsequent disease severity (Cox et al., 2012; Edwards et al., 1998; Limesand et al., 2000; Schneider et al., 2006). A similar observation has been made for ticks and biting flies (Dessens and Nuttall, 1998; Peters et al., 2008). When arboviruses are transmitted by mosquitoes, they replicate and disseminate more effectively to the blood, which may increase both their chance of onward transmission and their ability to cause more pronounced disease. The experimental deposition of uninfected mosquito saliva alone, in the absence of a bite, is sufficient to mediate this effect (Conway et al., 2014; Le Coupanec et al., 2013; Limesand et al., 2000; Moser et al., 2015; Styer et al., 2011). Although work has begun to define the factors within mosquito saliva that modulate arbovirus infection, the mechanistic basis that explains these observations is not known. Saliva from biting mosquitoes has been shown to have potent effects on various mammalian biological processes to support successful blood feeding (Fontaine et al., 2011), although evidence to support clearly defined immune-modifying functions in vivo is lacking. Here, we define the mechanistic basis by which mosquito bites enhance arbovirus infection in vivo.

Due to the importance of type I interferons (IFNs) and T cell responses in anti-viral immunity, their functional perturbation by mosquito saliva during infection has often been hypothesized

This is an open access article under the CC BY license (http://creativecommons.org/licenses/by/4.0/).

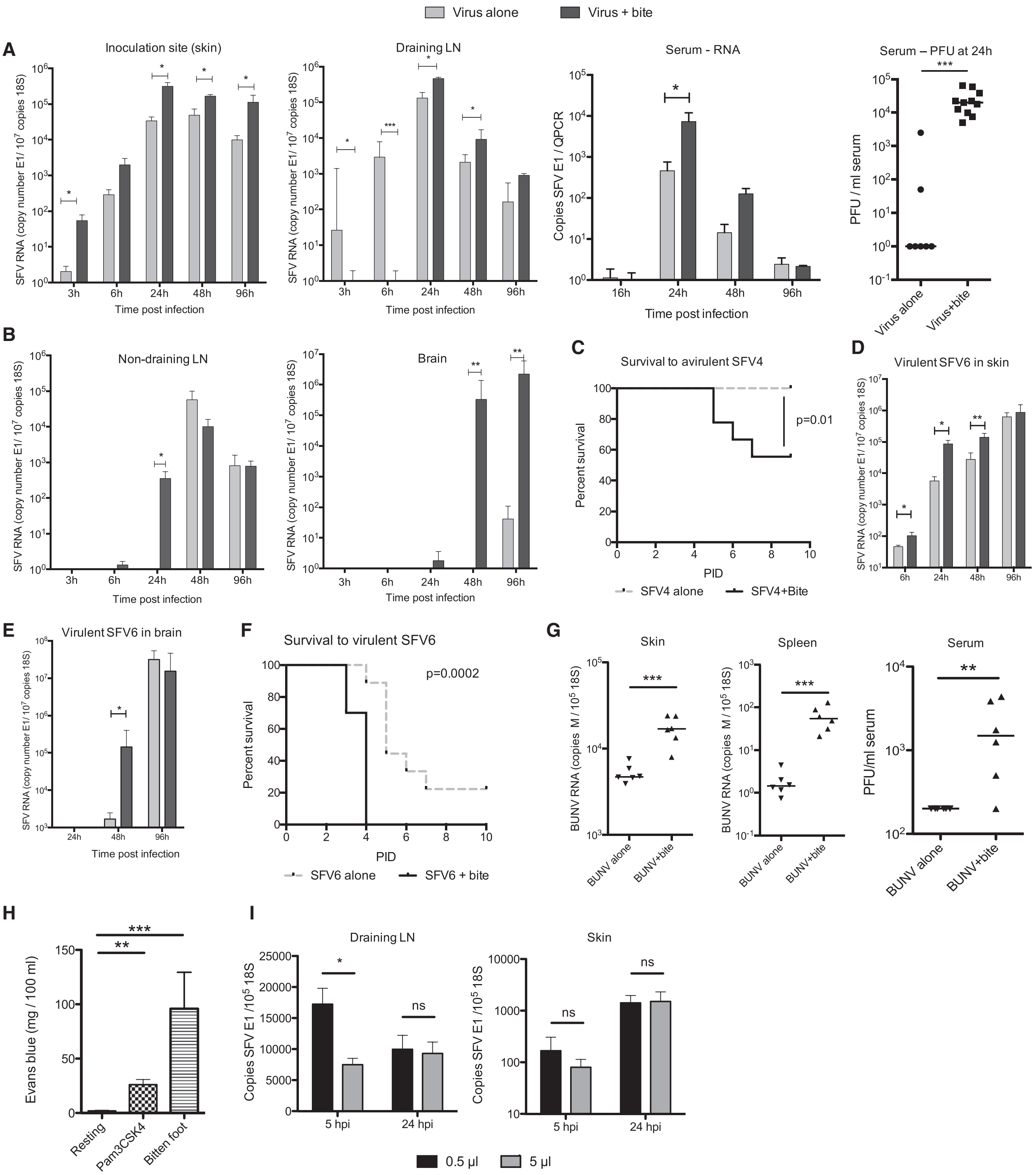

Figure 1. Mosquito Bites Facilitate Virus Retention and Replication at Cutaneous Inoculation Sites, Enhance Viraemia and Systemic Dissemination, and Increase Mortality to SFV Infection

(A and B) Mice were infected with 10^3 PFU of SFV4 either in presence (dark gray bars) or absence (light gray bars) of a mosquito bite. Copy number of SFV RNA (E1 gene) and host 18S was determined by qPCR. Virus titers in the serum were also quantified by plaque assay ($n \geq 7$).

(C) Mice were infected either in the presence (black line) or absence (grey dotted line) of a mosquito bite with SFV4 (n = 10).

(D and E) Mice were infected with 250 PFU SFV6 in presence (dark gray bars) or absence (light gray bars, n = 5) of a mosquito bite.

(F) Mice were infected either in the presence (black line) or absence (grey dotted line) of a mosquito bite with SFV6 (n = 10).

(legend continued on next page)

(McCracken et al., 2014; Schneider et al., 2004). However, we showed that rather than perturb anti-viral immune responses, mosquito bites triggered a leukocyte influx that facilitated infection by providing new cellular targets for infection. We identified a two-step process in which mosquito bites caused an influx of inflammatory neutrophils that helped coordinate innate immune responses and pave the way for the chemokine receptor CCR2-dependent entry of myeloid cells that are permissive to viral infection. Furthermore, mosquito bites elicited a pronounced edema that retained more of the virus inoculum in the skin and facilitated infection of these cutaneous cells. Inhibition of key components of the inflammatory response to the bite reduced leukocyte influx, suppressed viral replication, and increased host survival. These findings identify an important aspect of the host immune response to mosquito bites that inadvertently promotes concurrent arbovirus infection.

RESULTS

Mosquito Bites Enhance the Severity of Virus Infection

We developed an in vivo model system that makes use Semliki Forest virus (SFV), a mosquito-borne alphavirus that is a close relative of the chikungunya virus (Powers et al., 2001). We used *Aedes* mosquito cell-produced virus for inoculations to ensure that the first round of infection resembled the mosquito-transmitted virus as closely as possible. Unlike many human pathogens such as dengue and chikungunya viruses, SFV replicates and disseminates efficiently within both immune-competent mice and aedine mosquitoes (Ferguson et al., 2015; Rodriguez-Andres et al., 2012). SFV4 is an avirulent strain that rarely triggers clinical disease, whereas SFV6 is highly virulent and causes a lethal encephalitis (Ferguson et al., 2015; Michlmayr et al., 2014). After subcutaneous injection of SFV4 in the absence of mosquito bites, the virus rapidly disseminated to the draining popliteal lymph node (dLN) within 3 to 6 hr post-infection (hpi) (Figures S1A–S1D). A low-level viraemia peaked at 24 hpi with systemic spread to distal tissues apparent from 48 hpi onward, before occasional dissemination to the brain at 96 hpi (Figures S1A–S1C). Tissues identified by intravital imaging as positive for virus replication were dissected and further analyzed by quantitative (q)PCR to measure the level of viral RNAs (Figures S1B–S1D). The absence of detectable viral replication at remote sites before 48 hpi suggested that the majority of virus in the blood at 24 hpi was derived from cells infected at the inoculation site and the lymphoid tissue that drains it.

To determine whether mosquito bites had the ability to affect virus infection in this model, mice were bitten with *A. aegypti* mosquitoes and then immediately infected with a defined dose of SFV4 at the bite site in a 1 μL volume (Figures 1A and 1B). By clearly defining the number of mosquito bites to a restricted area of skin and by injecting a known titer of virus inoculum, it was possible to reproducibly infect either mosquito-bitten skin or resting unaffected skin. The presence of mosquito bites resulted in an order of magnitude higher virus RNA copy number in the skin at the inoculation site at most time points compared to unbitten mice and in the dLN and blood from 24 hpi onward. Virus infection with mosquito bites mediated earlier and greater dissemination of virus to remote lymphoid tissue and to the brain (Figure 1B) and in a significant proportion of the mice converted an avirulent infection into a lethal one (Figure 1C). In comparison, infection with the SFV6 strain of virus was fatal irrespective of the presence of mosquito bites at the inoculation site. As with SFV4, bites enhanced SFV6 infection at early time points, facilitating a more rapid dissemination to the brain, and mice succumbed earlier to infection (Figures 1D–1F and S1E). Mosquito bites also significantly enhanced infection with Bunyamwera virus (BUNV) (Figure 1G), a genetically unrelated RNA negative-sense virus that is also transmitted by *Aedes* mosquitoes (Elliott, 2014). BUNV, which otherwise struggles to replicate in wild-type mice after extraneural inoculation (Bridgen et al., 2001), demonstrated significantly higher replication and systemic spread to distal tissues when inoculated into mosquito bites. These results indicate that mosquito bites enhanced BUNV infection in vivo. Together these data suggest that early skin-centric processes triggered by mosquito bites have a substantial and defining impact on virus replication in vivo and that this impacts end-stage disease.

Mosquito Bite Sites Are Characterized by Extensive Edema that Is Associated with Retention of Viral Inoculum at the Bite Site

In the absence of bites, SFV disseminated rapidly to the dLN where copy number of virus RNA correlated with type I IFN expression in a time-dependent manner (Figure S1F). The presence of bites inhibited initial transfer of virus to the dLN (Figure 1A), which correlated with a delayed induction of gene transcripts for the prototypic IFN-stimulated gene (ISG) CXCL10 and also IFN-γ (Figure S1G). However, by 24 hpi, type I IFN and ISGs in the dLN were elevated compared to unbitten mice, correlating with higher virus RNA copy number at this time point (Figures 1A and S1H). To determine whether altered fluid flow within bitten skin could account for the suppression of early viral dissemination to the dLN at 6 hr, we quantified the level of vascular leakage at bite sites by injecting mice with Evans blue dye and measuring the amount dye that leaked from the circulation into the bite site (Figure 1H). At 3 hr after mosquito bite, the concentration of Evans blue detected in bitten skin was significantly higher compared to both resting skin and skin inflamed with the Toll-like receptor 2 (TLR2) agonist Pam3CSK4, a known inducer of edema (Supajatura et al., 2002). This suggested that a substantial bite-associated edema occurred that was associated with retention of virus in the skin. We hypothesized that the increased volume of tissue fluid delayed drainage of free virus, and that its retention was likely to facilitate infection of cutaneous cells and could also explain the delayed innate immune activation in the

(G) Mice were infected with 10^4 PFU BUNV in presence or absence of a mosquito bite (n = 6) and the level of viral RNA and infectious titer quantified at 24 hpi by qPCR and plaque assay. Gene expression of the virally encoded M-segment was used to assay BUNV RNA.

(H) Mice were injected with either saline or TLR2 ligand Pam3CSK4 or exposed to mosquito bites and edema determined at 3 hpi (n = 8).

(I) Mice were infected with 10^3 PFU SFV4 constituted in differing volumes and SFV E1 RNA copy number determined by qPCR (n = 8).

All column plots show the median value ± interquartile range. Results shown are representative of either two or three experiments. *$p < 0.05$, **$p < 0.01$, ***$p < 0.001$. See also Figure S1.

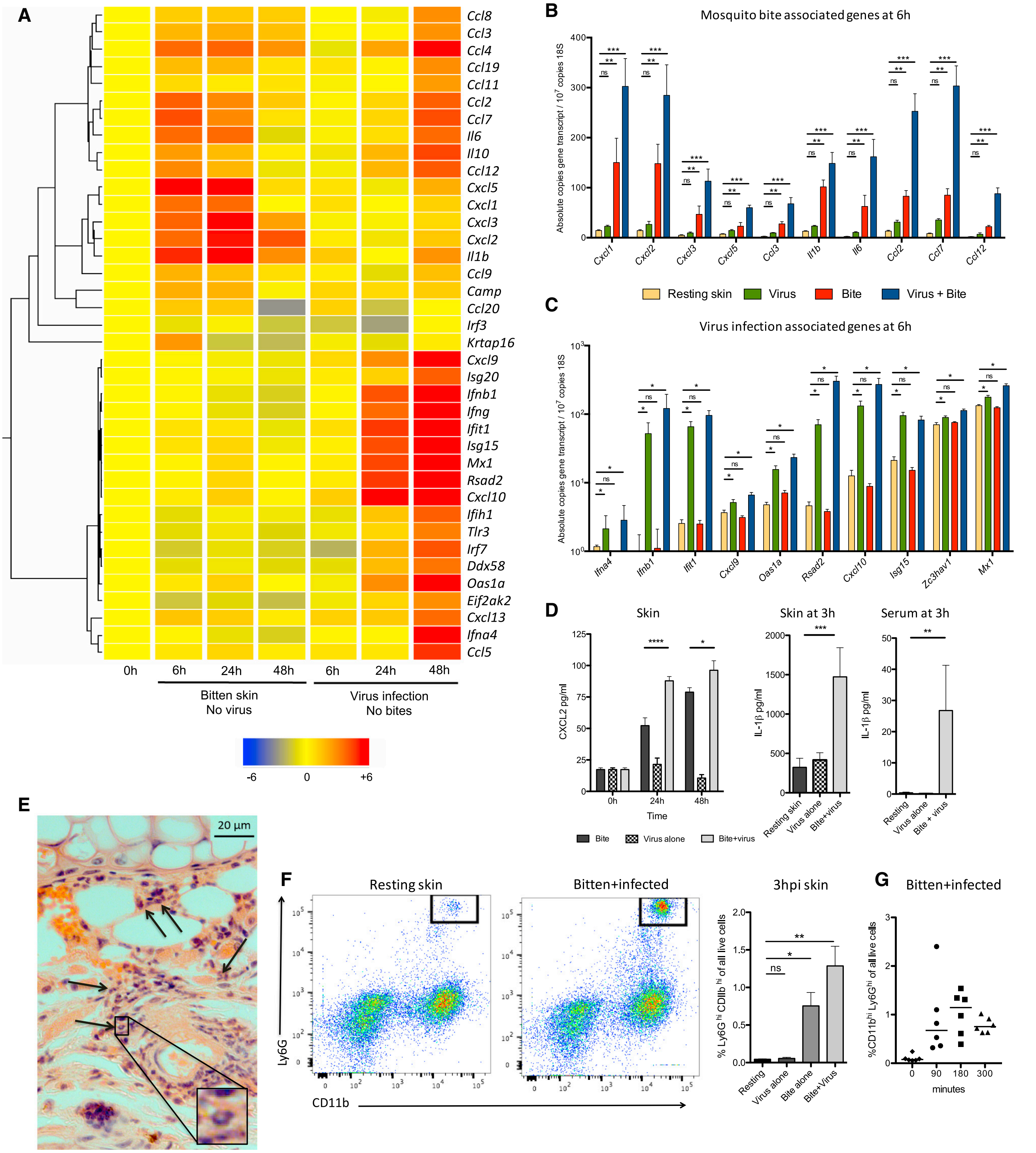

Figure 2. Mosquito Bites Induce the Rapid Recruitment of Neutrophils

(A) Cutaneous immune responses to bites alone, without concurrent virus infection, were compared to mice infected with SFV4 alone, in the absence of bites. Gene expression was determined using Taqman low-density arrays and hierarchical clustering undertaken to group genes with similar patterns of expression.

(B and C) Bite-associated gene expression (B) and SFV4 infection-associated genes (C) at 6 hr were validated by absolute qPCR (n $\geq$ 6).

(D) CXCL2 (n = 6) and IL-1β protein levels (n = 10) were determined by ELISA in skin samples from uninfected and SFV4-infected mosquito bite sites.

(E and F) Neutrophil infiltration was determined by histology (E) and flow cytometry (F) at 3 hr after bite or infection with SFV4.

(E) Tissue sections stained by H&E. Black arrows indicate typical multi-lobed nuclei of neutrophils.

(legend continued on next page)

dLN. To determine whether increased interstitial fluid alone could account for the delayed dissemination of virus to dLN, unbitten mice were inoculated with the same dose of virus in either a small volume (0.5 μL) or large volume (5 μL) inoculum of virus in albumin-containing saline. Those mice that received the virus in the larger volume demonstrated impaired spread of virus to the dLN at 5 hpi, although viral RNA copy number were similar at later time points (Figure 1I). In summary, mosquito bites induced an edema that retained virus in the skin, facilitating infection of cutaneous cells, but increased interstitial fluid alone is unlikely to account for enhancement of infection by mosquito bites at later time points.

Mosquito Bites and Virus Infection Combine to Induce a Substantial but Transient Increase in Cutaneous Neutrophils

To determine the mechanism by which mosquito bites enhanced virus infection, we next defined the effect that bites alone had on cutaneous innate immune responses. The expression of 64 key innate immune genes were assayed in the skin of mice that were either bitten with mosquitoes or infected with virus in the absence of mosquito bites. Hierarchical clustering identified a distinctive innate immune signature in bite sites that was dominated by neutrophil-attracting chemokines (CXCL1, CXCL2, CXCL3, and CXCL5) and the cytokines IL-1β and IL-6, which were not significantly induced by virus infection alone (Figures 2A–2C and S2). Genes also upregulated by bites included the monocytic chemoattractive chemokines CCL2, CCL4, CCL5, CCL7, and CCL12. This expression pattern was similar in bitten mice that had previously been exposed to multiple mosquito bites (Figure S3). In comparison to mosquito-bitten skin, the cutaneous gene expression signature of virus infection alone was both markedly delayed and dominated by type I IFN and ISGs (Figures 2A–2C and S2).

Induction of CXCL2 and IL-1β by bites was compared to other known inducers of neutrophil influx and to virus infection alone (Figures S4A and S4B). Despite the relatively discrete area of skin affected by biting, compared to that affected by injection of other innate immune stimulants, bites resulted in a substantial increase in gene transcripts, which was further validated at the protein level by ELISA (Figure 2D). Accordingly, mosquito bites elicited a substantial infiltrate of neutrophils from as early as 90 min onward, as demonstrated by histology (Figure 2E), flow cytometry analysis of Ly6G^{hi}CD11b^{hi}CD45^{+} neutrophils (Figures 2F and 2G), and qPCR analysis of neutrophil-specific markers (Figure S4E). Despite the inability of virus infection alone to induce substantial expression of CXCL2 or IL-1β, or Ly6G^{hi} CD11b^{hi}CD45^{+} neutrophil influx, infection combined with bites induced significantly higher expression and an enhanced neutrophil influx compared to bite alone (Figures 2D and 2F). Thus, although virus infection alone was not sufficient to induce the expression of most bite-associated genes, infection did nonetheless combine with mosquito bites in the induction of bite-associated genes and triggered a more pronounced neutrophil influx. In summary, neutrophil influx is a key aspect of virus-infected bite sites and that this is associated with enhanced viral replication. One possible explanation is that newly recruited neutrophils support viral replication by providing additional cellular target for virus infection, as is the case for *Leishmania* transmission by sandflies (Peters et al., 2008). However, we found that neutrophils at bite sites were refractory to infection (Figure S4F).

Mosquito Bites Do Not Subvert the Induction of Antiviral Immune Responses

Mosquito saliva contains a myriad of biologically active components such as vasodilators (Fontaine et al., 2011) and it has been hypothesized that it also has immune-modulating factors that facilitate host infection by a variety of pathogens (Schneider et al., 2004). Indeed, much effort is being made to identify these factors from a variety of blood-feeding arthropods (Déruaz et al., 2008; King et al., 2011). It has been suggested that mosquito bites promote a T helper 2 (Th2)-cell-dominated immune response that enhances virus replication (Schaeffer et al., 2015; Schneider et al., 2004). Although our kinetic data (Figure 1) and those of others (Styer et al., 2011) suggest that enhancement of infection by mosquito bites occurs too early for adaptive immunity in naive mice to impact on this process, we nevertheless looked at this possibility. Bite-associated enhancement of infection was apparent in severe combined immunodeficiency (SCID) mice, which lack T and B cells (Figure 3A), whereas classic Th1 or Th2 cytokines (e.g., IFN-γ, IL-4) could not be detected after mosquito biting of naive wild-type mice in the absence of virus infection (Figure S2). Prior exposure of mice to mosquito bites primed them to rapidly express cutaneous IFN-γ and IL-10 upon further mosquito biting (Figure 3B). However, we found that these bite-experienced mice did not demonstrate any increased susceptibility to, or protection from, bite enhancement of SFV4 infection compared to bite-naive mice (Figure 3C). Thus, mosquito bite enhancement of virus infection is independent of host cutaneous IFN-γ and does not require adaptive immunity.

We next determined whether mosquito bites suppressed cutaneous anti-viral innate immune responses. We assayed the transcriptional induction of IFNs and ISGs known to have important anti-viral functions (Figures 3D and 3E; Schoggins et al., 2011). However, rather than suppress cutaneous IFN-β induction to virus, mosquito bites resulted in enhanced cutaneous IFN-β transcript induction at 24 hpi (Figure 3D), probably as a direct result of higher virus replication as indicated by increased virus RNA copy numbers (Figure 1). Indeed, once the higher copy numbers of virus RNA present in the skin were accounted for, ISG induction by virus was mostly unaffected by mosquito bites (Figure 3E), suggesting that bites do not facilitate virus infection by suppressing the cutaneous induction of IFNs. All together these results indicate that mosquito bite enhancement of virus infection is not due to suppression or subversion of skin anti-viral immune responses by bites.

(F) Numbers represent percent of CD11b^{hi}Ly6G^{hi} cells of all live cells (n = 4).

(G) Neutrophils were present in high numbers by 90 min after bite/infection, peaking at 180 min.

All column plots show the median value ± interquartile range. Results shown are representative of either two or three experiments. *$p < 0.05$, **$p < 0.01$, ***$p < 0.001$. See also Figures S2–S4.

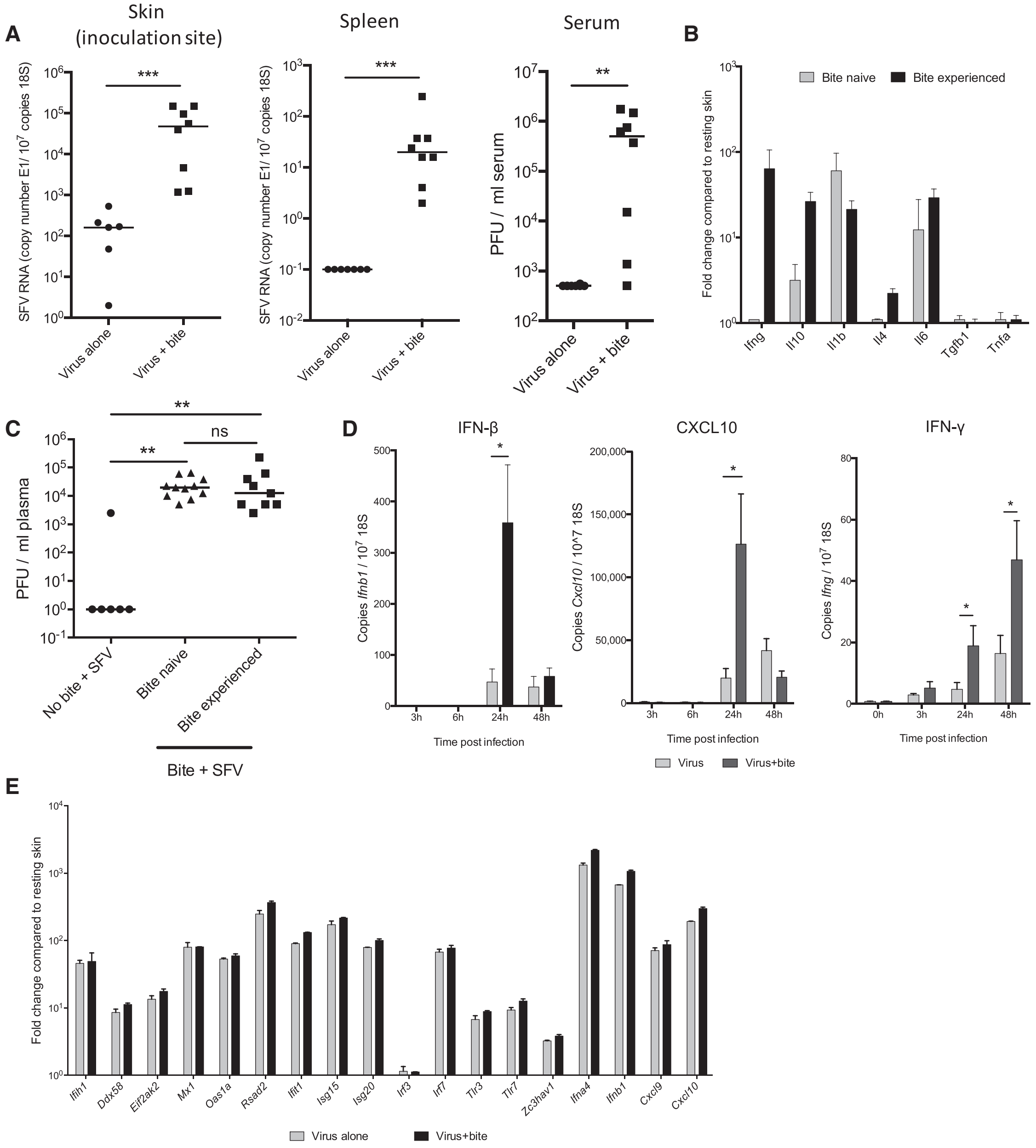

Figure 3. Mosquito Bites Do Not Subvert the Induction of Antiviral Immune Responses

(A) SCID mice were infected with 10^4 PFU SFV4, either in presence or absence of mosquito bites and the levels of viral RNAs determined (n = 8).

(B and C) Wild-type mice were either subjected to four sessions of mosquito biting at 1-week intervals or left unexposed to mosquitoes (bite-naive). Mice were then subjected to mosquito biting and gene transcripts assayed in skin at 6 hr after bite (B) or infected with 10^4 PFU SFV4 into the bite site and serum viraemia determined at 24 hpi (C) ($n \geq 6$).

(D and E) Cutaneous type I IFN and ISG gene expression was enhanced by the presence of mosquito bites. Mice ($n \geq 5$) were infected with SFV4 either in presence or absence of mosquito bites and the cutaneous expression of transcripts determined by absolute qPCR (D), or fold change determined by TLDA (E). Fold change was calculated by comparison to resting skin and was normalized to the level of SFV E1 copy number.

All column plots show the median value ± interquartile range. *$p < 0.05$, **$p < 0.01$, ns = not significant.

Neutrophils Drive a Cutaneous Pro-inflammatory Program that Inadvertently Facilitates Virus Infection

We hypothesized that if recruited neutrophils are not the target of virus infection themselves, they might nonetheless be instrumental in coordinating the tissue response to mosquito bite trauma that supports enhanced virus infection. Neutrophils have long been established as pivotal regulators of vascular permeability, edema, and the influx of myeloid cells (Charmoy et al., 2010; Nakamura et al., 2009; Wedmore and Williams, 1981). Although much of the vascular leakage at bites is probably due to vessel rupture by probing mosquitoes, neutrophils enhance this leakage, as shown by the fact that depletion of

neutrophils (using the IA8 antibody that binds neutrophil-expressed Ly6G) significantly reduced edema (Figures 4A and S5A). Depletion of neutrophils also greatly impaired the induction of many bite-associated genes at 6 hpi (Figures 4B and 4C), whereas in non-depleted mice, neutrophil-expressed CXCR2 copy number correlated with many of these genes (Figure 4D), suggesting that neutrophil influx into the bite site was required for their induction. Bite-associated genes that required neutrophil influx included IL-1β and the monocyte-attracting chemokines CCL2, CCL7, and CCL12 (Figure 4C). Almost all IL-1β-expressing cells at bite sites were positive for Ly6G and CD11b by flow cytometry, suggesting that infiltrating neutrophils themselves were the main cellular source of this cytokine (Figures 4E–4G). In comparison, those genes identified as specifically upregulated by virus infection alone in Figure 2, were unaffected by neutrophil depletion at 6 hpi (Figure 4H). In summary, inflammatory neutrophils that express IL-1β are required for the induction of cutaneous inflammatory responses to mosquito bites.

Next, we determined whether neutrophil-dependent inflammation is required for bite enhancement of infection. Depletion of neutrophils reduced SFV4 E1 RNAs by 5-fold at both the skin inoculation site, dLN, and distal lymphoid tissue at 24 hpi (Figures 4I and 4J), while viraemia was reduced 10-fold, such that it was not significantly different to unbitten controls (Figure 4K). Furthermore, the early dissemination of virus to dLN observed in unbitten mice was also partially restored in bitten mice in the absence of neutrophils, suggesting that lymph flow had transported more of the initial inoculum to the draining LN (Figure 4L). Thus, mosquito bites triggered a seemingly counter-productive neutrophil-dependent response that enhanced SFV infection at cutaneous inoculation sites.

To ascertain whether this effect is specific to mosquito bites, we determined whether innate immune agonists that also trigger neutrophil influx could similarly enhance infection. These included a phorbol ester (TPA), alum, and the TLR2 ligand Pam3CSK4, all of which induced cutaneous CXCL2 and IL-1β expression in our mice (Figures S4A and S4B). We compared the ability of these agents to enhance SFV4 infection to that of mosquito bites and separately to that of injected mosquito saliva, which had been previously obtained from female *A. aegypti* mosquitoes. All these agents substantially enhanced SFV4 replication in the skin and dissemination to the blood despite them having no known structural similarity to each other (Figure 5A). Furthermore, as with mosquito bites, enhancement of SFV4 infection by Pam3CSK4 and phorbol ester occurred in a neutrophil-dependent manner (Figures 5B and 5C) and was not due to a suppression of type I IFN induction (Figure S6). Pam3CSK4 was also able to significantly enhance infection with the unrelated BUNV (Figure 5D). Together this suggests that mosquito-sourced factors at bite sites do not enhance infection via any specific evolved function, but instead by their inadvertent ability to promote a neutrophil-dependent inflammation after a bite.

Therapeutic Inhibition of the Inflammasome Inhibits Neutrophil Influx and Prevents Bite Enhancement of Virus Infection

We next determined whether it was possible to alter the outcome of infection by modulating host immune responses to bites. Because neutrophil depletion was so effective at suppressing bite enhancement of infection (Figure 4), we first determined whether neutrophil depletion could also decrease mortality with SFV6 infection. However, we found that neutrophils were required for protection from SFV6 infection irrespective of the presence of mosquito bites, with roughly 50% of neutrophil-sufficient mice surviving infection, compared to 10% of mice surviving in the absence of neutrophils (Figure S5C). Together these data suggest that although neutrophils initiate counterproductive responses at mosquito bites for the host, they are nonetheless required at later stages of disease to prevent mice from succumbing to infection.

Although the wholesale depletion of neutrophils is clearly inappropriate for preventing bite-enhanced SFV infection, a more refined interventionist approach would be to reduce neutrophil recruitment to bitten skin while leaving systemic neutrophil numbers untouched. Because neutrophil-derived IL-1β expression was a key feature of mosquito bites and because inflammasome activation has been implicated in enhancing neutrophil influx (Nakamura et al., 2009), we wanted to determine whether it was possible to target this pathway. We first wanted to confirm that mosquito bite enhancement of virus infection was dependent specifically on IL-1β. Accordingly, *Il1r1*$^{-/-}$ mice demonstrated both a deficiency in neutrophil recruitment to infected bite sites and were not susceptible to bite-mediated enhancement of virus infection (Figures 6A–6C), identifying this pathway as a putative therapeutic target.

We next determined the efficacy of the well-characterized caspase-1-specific antagonist Z-YVAD-FMK (Jabir et al., 2014) in preventing bite enhancement of virus infection. After Z-YVAD-FMK treatment, mosquito-bitten virus-infected mice demonstrated significantly less serum IL-1β (Figure 6D), a reduction in the cutaneous expression of bite-associated CXCL2 and IL-6 (Figure 6E), and fewer cutaneous neutrophils (Figures 6F and 6G). We next determined whether the innate immune suppression of bite-associated inflammation provided by inflammasome inhibition affected the replication and dissemination of SFV. Importantly, Z-YVAD-FMK administration had no effect on viral titers by 24 hpi in the absence of mosquito bites (Figure 6H), suggesting that this agent has no intrinsic anti-viral effect. However, inflammasome inhibition was highly efficacious at lowering viral titers by 24 hpi in mice infected at mosquito bite sites (Figures 6I and 6J). Copies of SFV4 RNA in the brain at day 5 were low or undetectable in treated mice, whereas treated SFV6-infected mice had an increased survival rate (Figures 6K and 6L). Together, these observations show that the IL-1β pathway promoted mosquito bite inflammation, that this was necessary for the effective replication and dissemination of virus from the bite site, and that these in turn determine the subsequent systemic course of infection and clinical outcome in the mouse.

CCR2-Dependent Migration and Infection of Myeloid Cells Is Required for Mosquito Bite Enhancement of Infection

Finally, we wanted to determine the mechanism by which inflammation augmented virus infection. We first defined which cell types were responsible for supporting enhanced viral replication at bite sites. Lyve1 is a protein expressed by both lymphatic

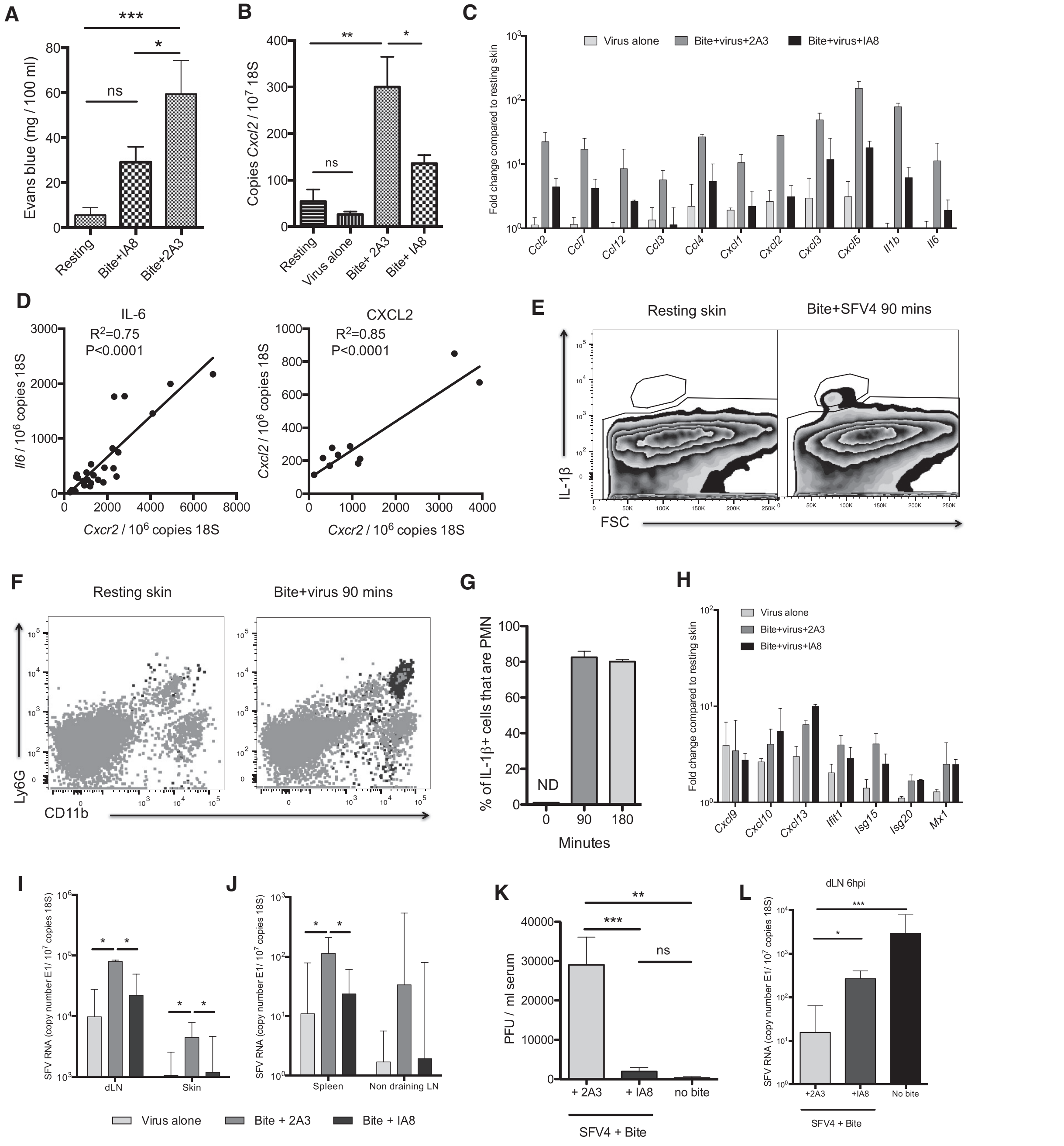

Figure 4. Mosquito Bite-Infiltrating Neutrophils Express IL-1β, Co-ordinate Innate Immune Gene Expression, and Are Required for the Effective Replication and Dissemination of Virus

(A–C) Mice were either depleted of neutrophils by i.p injection of the IA8 antibody or control treated with non-depleting 2A3 control antibody, bitten with mosquitoes, and infected with 10^4 PFU SFV4 ($n \geq 6$).

(A) Edema in skin was quantified after i.p. injection of evans blue at 4 hpi.

(B) CXCL2 transcripts were determined by qPCR in the skin of mice at 6 hr, n = 12.

(C) Skin-bite-associated genes were assayed by qPCR at 6 hpi. Untreated, SFV4-infected mice were included for comparison.

(D) IL-6 (n = 26) and CXCL2 (n = 12) transcripts correlated with the number of CXCR2 transcripts in mosquito-bitten skin.

(E–G) Skin biopsies at 90 min after bite or SFV4 infection were digested to release cells and stained for flow cytometry. Cells were gated based on their IL-1β staining (E) and back-gated onto a Ly6G/CD11b plot as dark gray dots (F). The percent of IL-1β cells that were $Ly6G^{hi}CD11^{hi}$ at 90 min after bite/infection (n = 6, ND = not detected) (G).

(H) Skin virus-associated genes were assayed by qPCR at 6 hpi. Untreated, SFV4-infected mice were included for comparison.

(legend continued on next page)

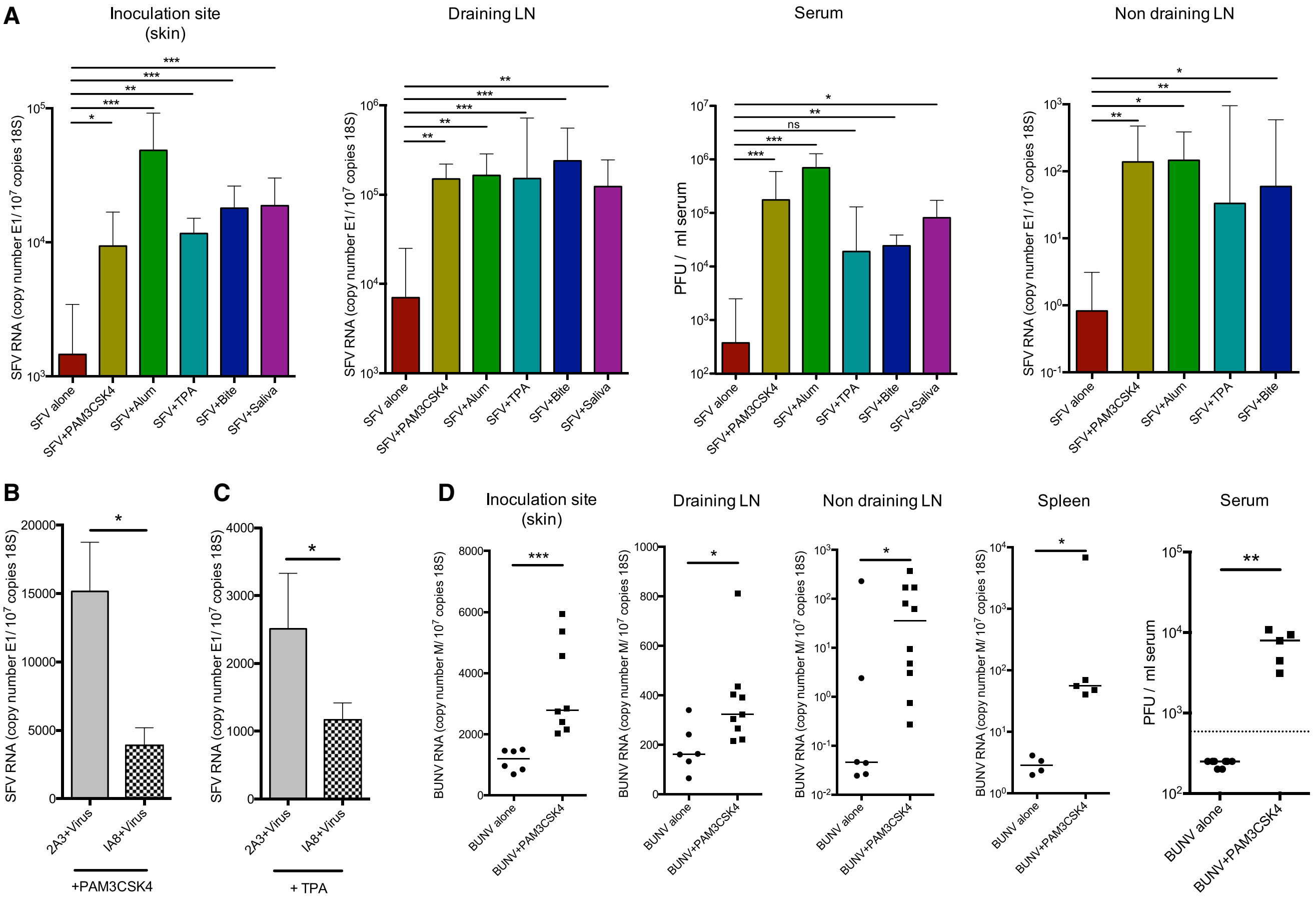

Figure 5. Structurally Unrelated Pro-inflammatory Agents and Mosquito Saliva Promote Virus Infection

(A) After s.c. administration of TLR2 ligand Pam3CSK4, alum, or mosquito saliva or after mosquito biting or topical application of the phorbol ester TPA, mice were infected with 10^4 PFU SFV4 at the same site ($n \geq 5$), and the level of viral RNA determined by qPCR and infectious titer determined by plaque assay at 24 hpi.

(B and C) Mice were depleted of neutrophils using the IA8 antibody or given 2A3 control antibody, treated with a s.c. injection of Pam3CSK4 (B) or topical application of TPA (C), and then infected with 10^4 SFV4 at the same cutaneous site. At 24 hpi, SFV RNA copy numbers were determined in the skin (n = 5).

(D) After s.c. administration of the TLR2 ligand Pam3CSK4, mice were infected with 10^4 BUNV, and the level of viral RNA (M-segment) and infectious titer quantified at 24 hpi.

All column plots show the median value ± interquartile range. *$p < 0.05$, **$p < 0.01$, ***$p < 0.001$, ns = not significant. See also Figure S6.

endothelial cells and CD45$^+$F4/80$^+$CD11b$^+$ macrophages in the skin, which can be differentiated from each other based on their morphology (Schledzewski et al., 2006) and by flow cytometry (Figure S7). After infection with enhanced green fluorescent protein (EGFP)-expressing SFV, we found large numbers of EGFP$^+$ Lyve1$^+$ cells in the dermis (Figure 7A). Lymphatic endothelial cells did not express EGFP (data not shown), although Lyve1$^+$ macrophages were positive for SFV-EGFP at 6 hpi (Figures 7A and 7B). Between 4 and 16 hpi, large numbers of CD11b$^+$Ly6C$^+$ Ly6G$^-$ myeloid cells infiltrated bite sites and numbers of these cells were increased in virus-infected bite sites, whereas in the absence of bites, virus infection alone elicited little influx of these cells (Figure 7C). A subset of these cells were positive for virally encoded EGFP (Figures 7D and 7E). We found that by 24 hpi in virus-infected bite sites, there was a selective loss of Lyve1$^+$ F4/80$^+$CD11b$^+$ cells, whereas numbers did not decrease in bite sites in the absence of virus, suggesting that they had been depleted after infection with this often cytolytic virus (Figures 7F and S7B).

Infection of myeloid cells and dendritic cells in skin has also been reported during infection with dengue virus in the absence of mosquito bites (Pham et al., 2012; Schaeffer et al., 2015; Schmid and Harris, 2014), suggesting that several arboviruses might have evolved to take advantage of the myeloid cell infiltrate at bite sites to enhance replication. To determine whether infection of myeloid cells at bite sites results in the release of

(I–K) At 24 hpi, SFV RNA copy number was determined in dLN and inoculation site (skin) (I) and in lymphoid tissues distal to inoculation site (J) by qPCR, n = 7, and infectious virus in the serum determined (K) n = 10.

(L) SFV RNA was quantified at 6 hr in the draining popliteal LN to determine whether neutrophil depletion could overcome the initial block imposed by bites on early virus dissemination (n = 7).

All column plots show the median value ± interquartile range. Results shown are representative of either two or three experiments. See also Figure S5.

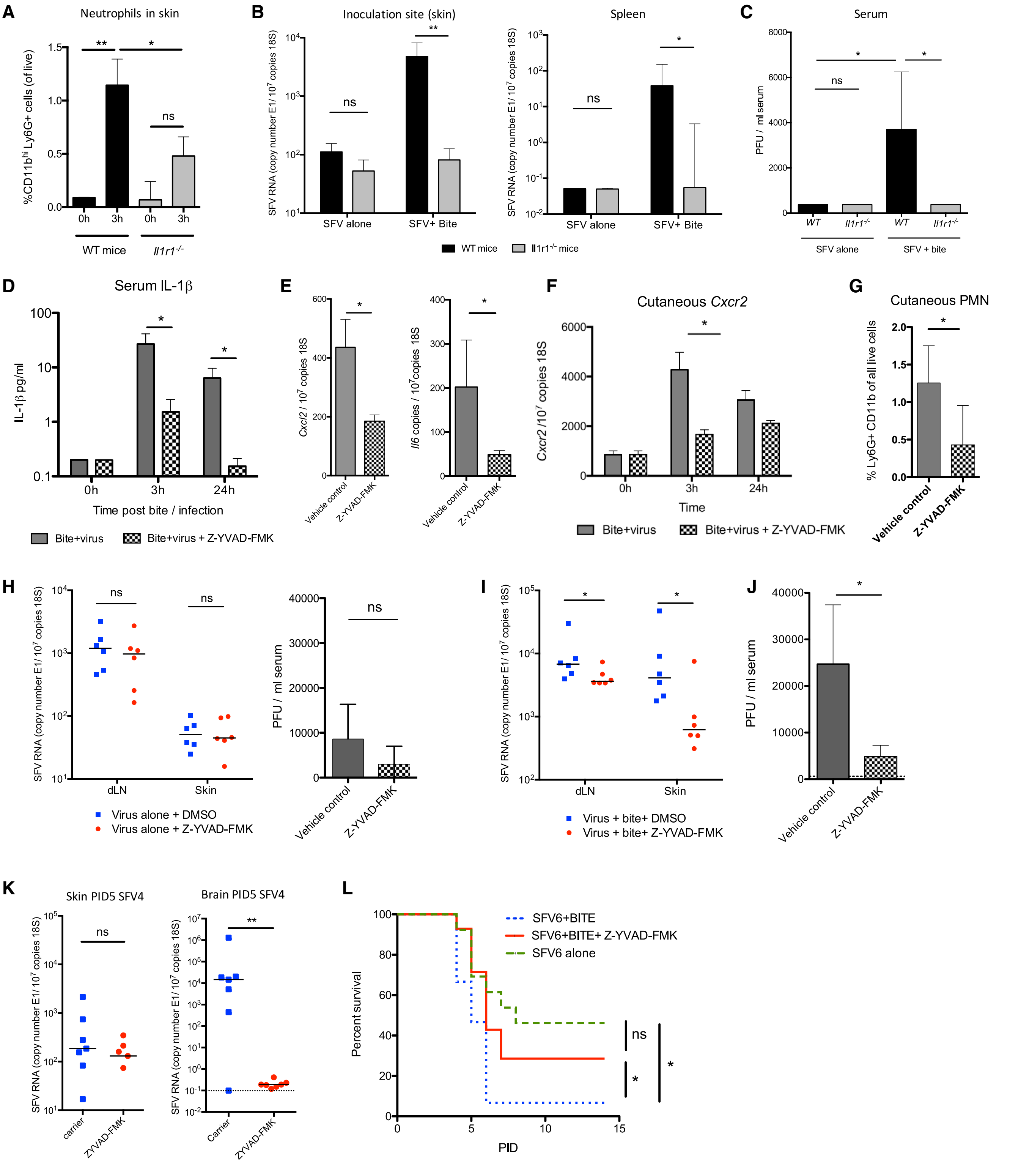

Figure 6. Mosquito Bite Enhancement of Virus Infection Is Dependent on IL-1β

(A) Numbers of neutrophils infiltrating SFV4-infected bite sites were quantified in WT and *Il1r1*$^{-/-}$ mice (n = 6).

(B and C) WT and IL-1R-null mice (n ≥ 6) were infected with 10^4 SFV4 at mosquito bite sites or resting skin. SFV RNA copy numbers (B) and viraemia were determined (C) at 24 hpi.

(D–G) Mice were treated with the caspase-1 inhibitor Z-YVAD-FMK i.p. (1.5 mg/kg) or vehicle control, bitten by mosquitoes, and then infected with 10^4 SFV4 at the bite site.

(legend continued on next page)

new infectious virus, we isolated cutaneous cells at 16 hpi, magnetically purified CD11b$^+$ cells on columns (Figures 7G and S7C), and quantified the amount of virus released after 6 hr in culture by plaque assay (Figure 7H). On a per-cell basis, the CD11b$^-$ fraction released little new infectious virus and the CD11b$^+$ enriched fraction released substantially more, demonstrating that CD11b$^+$ cells are capable of releasing high amounts of infectious virus.

To determine the contribution that myeloid cell-derived virus makes toward viral replication and dissemination in vivo, we made use of mice that lack the chemokine receptor CCR2. These mice are monocytopenic and are deficient in all dermal bone marrow-derived macrophages (Serbina and Pamer, 2006; Tamoutounour et al., 2013). Compared to WT mice, infection of *Ccr2*$^{-/-}$ mice at bite sites resulted in similar increases in innate immune gene expression and neutrophil influx and demonstrated similar virus titers at 4 hpi but failed to elicit an influx of myeloid CD11b$^+$Ly6C^{hi} cells (Figures 7I, 7J, S7D, and S7E). *Ccr2*$^{-/-}$ mice were protected from bite enhancement of SFV replication (Figure 7K). Mosquito bites also significantly enhanced infection with BUNV in a CCR2-dependent manner (Figure 7L). *Ccr2*$^{-/-}$ mice were not intrinsically less susceptible to infection because in the absence of bites, SFV and BUNV titers were similar to WT mice at 24 hpi (Figures 7K and 7L). Together this demonstrates that the inability of *Ccr2*$^{-/-}$ mice to elicit a myeloid cell influx into bite sites prevented enhancement of infection. In summary, we show that mosquito bites enhance mosquito-borne virus infection by promoting the recruitment and infection of bone marrow-derived myeloid cells that release new infectious virus and in doing so enhance the replication and dissemination of virus to the blood and remote tissues.

DISCUSSION

The interface between mammals and mosquitoes at bite sites is an important and common stage of all arbovirus infections. This crucial aspect of arbovirus transmission is a bottleneck that can limit dissemination of virus to the bloodstream and the development of clinically apparent disease. Although mosquito saliva is well described as a potent enhancer of infection for several evolutionary distinct and medically important arboviruses, the cellular and molecular events that are responsible were not well defined. Previously, it had been suggested that mosquito saliva might modulate host immunity to create an immunosuppressed niche, as has been shown for biting ticks that suppress chemokine function (Déruaz et al., 2008). Ticks have evolved a unique repertoire of immunosuppressive factors, which most likely reflects their necessity to remain embedded in mammalian skin for many days. In comparison, mosquitoes feed only transiently and host immune responses are unlikely to exert sufficient effect on feeding efficiency to drive the evolution of vector-derived immunomodulatory factors. Instead we show that mosquito bite enhancement of virus infection results from virus replication in myeloid cells recruited to mosquito bites by a neutrophil-driven inflammasome-dependent, edematous inflammation. Whether the recruitment to bite sites of these cells is serendipity for the virus or whether the virus has evolved to replicate in these cells is not clear. However, the consequences are profound and affect the subsequent systemic course and clinical outcome of the infection. That this phenomenon occurs with two genetically distinct arboviruses from separate families suggests that it might also occur with many other mosquito-transmitted viruses.

We found that the neutrophil influx to mosquito bites was essential for coordinating the tissue response to this insult, without which innate immune gene expression was severely curtailed. This is somewhat similar to host responses to *Leishmania* infection, in which neutrophil recruitment is necessary for driving chemokine CCL3-dependent influx of dendritic cells to the skin (Charmoy et al., 2010). Separately, although we cannot discount the possibility that putative Th2-cell-driven allergic responses to bites in mosquito-experienced BALB/c mice (Schneider et al., 2007) might also a have role in potentiating virus infection, the ability of bites to fully potentiate virus infection in SCID mice suggests that they are dispensable in our model system.

Therapeutic blockade of caspase-1 and neutrophil depletion successfully suppressed cutaneous innate immune responses to bites and thus prevented virus from gaining a replicative advantage. Speculatively, suppressing specific aspects of the host response to mosquito bites might prove efficacious in preventing the onset of disease. We suggest a strategy that minimizes mosquito biting (e.g., use of repellents) combined with post-exposure prophylaxis at bite sites as an effective strategy for limiting mosquito-borne virus infection, especially for infections transmitted by *Aedes* mosquitoes that preferentially bite during the day. As a corollary, our data also suggest that approaches that aim to prevent natural arbovirus infection through vaccination to mosquito salivary components, while having much potential, should be carefully designed not to enhance cutaneous inflammation, and might explain why some vaccines to saliva can worsen outcome (Reagan et al., 2012). Our findings also have implications for the development of new "naturalized" models of virus infection that make use of mosquito saliva enhancement. Many viruses, including important human pathogens such as chikungunya virus (Teo et al., 2012), dengue virus (Zompi and Harris, 2012), and bunyaviruses (Bridgen et al., 2001), struggle to replicate and disseminate within wild-type mice after infection by needle into resting skin. Accordingly,

(D) Serum IL-1β expression was determined by ELISA (n = 10).

(E) Cutaneous bite-associated innate immune transcripts CXCL2 and IL-6 were determined by qPCR at 3 hpi (n = 5).

(F and G) Neutrophil influx into bite sites (n $\geq$ 6) was ascertained by assaying cutaneous CXCR2 expression (F) and the frequency of Ly6G^{hi}CD11b$^+$SSC^{hi} cells by flow cytometry at 3 hpi (G).

(H–K) Mice were treated with Z-YVAD-FMK i.p. and infected with 10^4 SFV4 in the absence (H) or presence (I–K) of mosquito bites. SFV RNA was determined by qPCR at 24 hpi (I) and at PID5 (K). Viraemia at 24 hpi was determined by plaque assay (J) (n = 6).

(L) Survival curve of mice infected with SFV6 at bite sites with or without treatment with caspase-1 inhibitor Z-YVAD-FMK i.p. at time of infection (n = 15). Results shown are representative of either two or three experiments.

All column plots show the median value ± interquartile range. $^*p < 0.05$, $^{**}p < 0.01$.

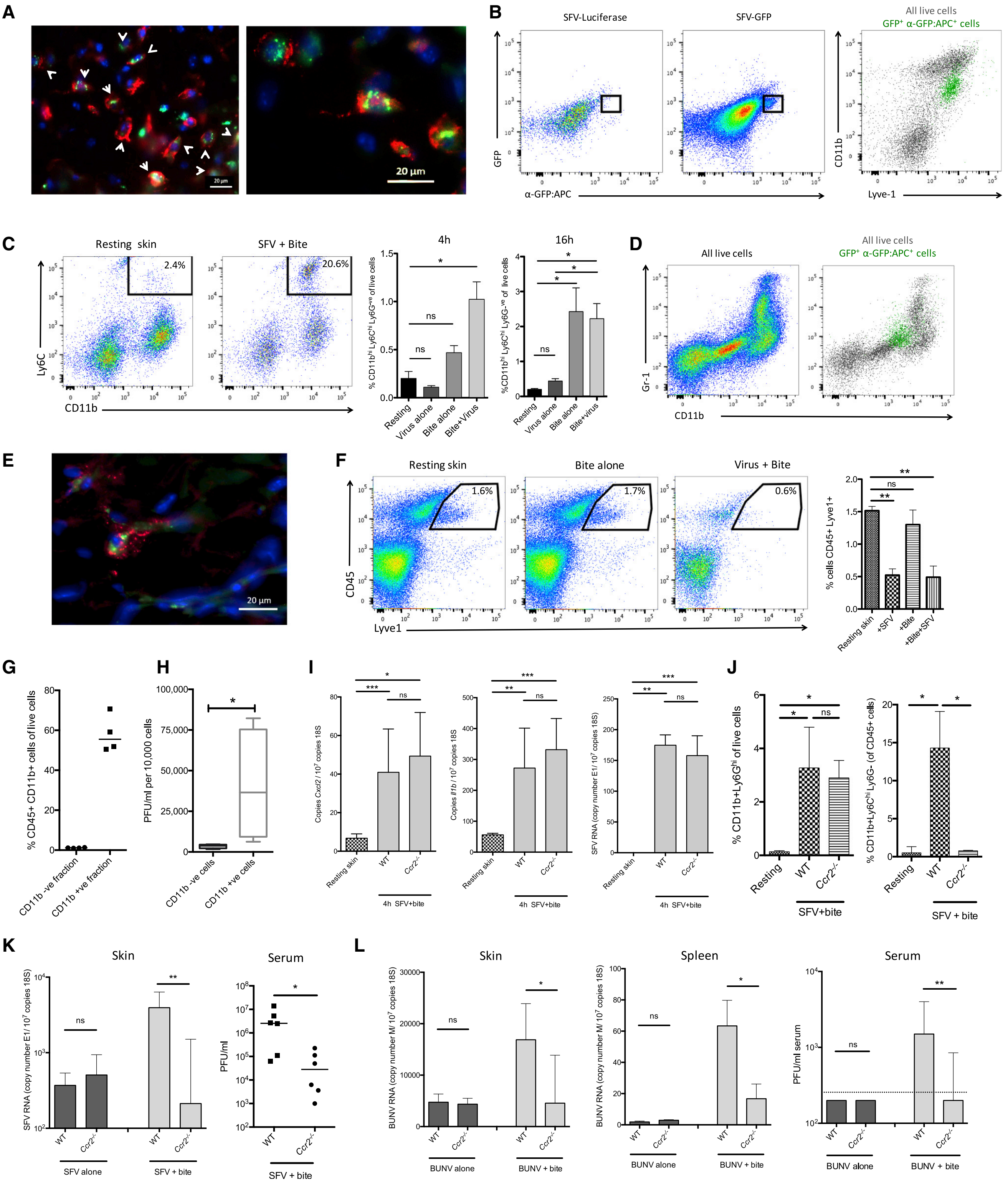

Figure 7. CCR2-Dependent Migration and Infection of Myeloid Cells Is Required for Mosquito Bite Enhancement of Virus Infection

(A) Mice were infected intradermally with SFV4(Xho)-EGFP (green) in back dorsal skin and at 6 hpi sections stained for lyve1 (red) and DAPI (blue). Arrows indicate double-positive cells in lower magnification image.

(B) Skin SFV4(Xho)-EGFP-infected cells were identified by both their expression of EGFP and staining against EGFP and back-gated onto a CD11b/Lyve1 plot, represented as green dots. Luciferase-expressing SFV4 was used as a control.

(legend continued on next page)

immunosuppressed mice are often used as an alternative, which precludes the experimental study of many aspects of host immune responses to these infections. Building on the work of other important studies (Conway et al., 2014; Cox et al., 2012; McCracken et al., 2014; Moser et al., 2015; Schneider et al., 2006; Styer et al., 2011), we suggest that the inclusion of mosquito bites, or their saliva, might sufficiently enhance viral replication to enable the study of these infections in wild-type mice.

In conclusion, we highlight an important aspect of host innate immunity at mosquito bite sites. These findings not only define the mosquito bite site as a putative target for post-exposure prophylactic intervention, but also pave the way for the development of in vivo models that better recapitulate an important aspect of mosquito-borne virus infection.

EXPERIMENTAL PROCEDURES

Detailed methodology is described in the Supplemental Information.

Cell Culture, Viruses, and Mice

Aedes mosquito cells and BHK cells were cultured using established protocols (Rodriguez-Andres et al., 2012). Details of reporter viruses can be obtained from the authors. The pCMV-SFV4 backbone for production of SFV4 has been previously described (Rodriguez-Andres et al., 2012; Ulper et al., 2008). Plasmids containing cDNAs of SFV were electroporated into BHK cells to generate infectious virus and then passaged once in mosquito cells. Titration of virus stocks and quantification of viraemia in vivo were performed via plaque assays (Rodriguez-Andres et al., 2012). Wild-type BUNV was generated as previously described (Bridgen et al., 2001). All mice were maintained under specific-pathogen-free conditions at the Central Research Facility, University of Glasgow. All mice were housed in accordance with local and Home Office regulations.

Mosquito Biting of Mice and Virus Infection

Anesthetized mice were positioned to expose a defined area of the left foot to a cage of *A. aegypti* mosquitoes. The remainder of the mouse body surface was protected. Mice were monitored during mosquito biting and a maximum of five mosquitos were allowed to engorge from the area exposed. Allowing more than one mosquito to probe/bite the available skin surface ensured that most of the exposed skin surface was subjected to probing/bites. Immediately after completion of mosquito biting, the bitten skin was injected with 1 μL of virus using a Hamilton Syringe (Hamilton). This approach enabled the effect of bites on concurrent virus infection to be quantifiably compared to virus infection alone in the absence of a bite.

RNA Extraction, Gene Expression Analysis, Flow Cytometery, and Immunohistochemistry

RNA was extracted using PureLink Plus columns and converted to cDNA using the High Capacity RNA-to-cDNA kit (Life Technologies). qPCR analysis was undertaken using SYBR-green PerfeCTa (Quanta) and TLDAs were used as per manufacturer's instructions on a 7900HT Real time machine (Applied Biosystems). ELISAs were undertaken using Duoset kits (R&D Systems). For flow cytometry, skin was enzymatically digested to release cells and stained using a subset of antibodies. Cells were stained with Fixable Viability Dye eFluor780 (eBioscience), fixed in 4% methanol-free paraformaldehyde (Thermo Scientific) or Cytofix/Cytoperm (BD), and analyzed on a MACSQuant Analyzer 10 (Miltenyi). For cell sorting, cells were labeled with CD11b beads and sorted on magnetized columns (Miltenyi). For immunohistochemistry, skin was fixed before freezing in embedding medium and sectioned. Sections were blocked in Tris-Saline-Tween (TBS)/5% fish gelatin (Sigma-Aldrich) incubated with a primary antibody against Lyve-1 and the secondary chicken anti-goat IgG Alexa Fluor 647-conjugated antibody (Life Technologies).

Statistical Analysis

Data were analyzed using the non-parametric-based tests Mann-Whitney or Kruskal-Wallis test with Dunn's multiple comparison. All column plots show the median value ± interquartile range with $^{*}p < 0.05$, $^{**}p < 0.01$, $^{***}p < 0.001$, $^{****}p < 0.0001$, ns = not significant. Wherever possible, preliminary experiments were performed to determine requirements for sample size, taking into account the available resources and ethical use of animals. Animals (gender and age matched) were assigned randomly to experimental groups. For plaque assays, samples were coded and analyzed blind by a separate investigator. All results shown are representative of either two or three experiments and where possible incorporate a variety of techniques and approaches. Importantly, biological replicates were excluded from analysis if s.c. or i.d. injection of virus inadvertently punctured a blood vessel.

SUPPLEMENTAL INFORMATION

Supplemental Information includes seven figures and Supplemental Experimental Procedures and can be found with this article online at http://dx.doi.org/10.1016/j.immuni.2016.06.002.

AUTHOR CONTRIBUTIONS

Conceptualization, C.S.M., J.K.F., and G.J.G.; Methodology, C.S.M., E.S., A.K., J.K.F., and G.J.G.; Investigation, M.P., S.R.B., E.P., E.S., and C.S.M.; Resources, E.S., A.K., E.P., A.M., and G.J.G.; Original draft, M.P. and C.S.M.; Review & Editing, all authors; Funding acquisition, C.S.M.

ACKNOWLEDGMENTS

We acknowledge the kind assistance of Melanie McFarlane and Stephanie Rainey for the provision of virus stocks and insect cell culture; Ryan Ritchie for help with IVIS; Margaret McFadyen and Joy Kean for culture of mosquitoes; Prof. Eileen Devaney for her detailed editing of the manuscript; and the Institute of Infection, Immunity and Inflammation Flow Cytometry Facility, all based at the University of Glasgow. This work was funded by the MRC (G1001724) and a BBSRC PhD studentship to S.R.B.

(C) Quantification of CD11b^{+}Ly6C^{hi} myeloid cell numbers in bite sites at 4 and 16 hr. Live cells were first gated for CD45^{hi} cells and to remove Ly6G^{hi} neutrophils (n = 4).
(D) CD11b and Gr-1 staining for all cells (left) and back gating of virus-infected cells at 16 hpi (green dots, right plot).
(E) Skin sections were stained for the bone-marrow-derived myeloid marker ER-HR3 (red) in SFV4(Xho)-EGFP-infected skin at 16 hpi.
(F) Percentage CD45^{+}Lyve1^{+} macrophages numbers of all live cells at 24 hpi in the skin (n = 4).
(G and H) SFV4-infected bite sites (n = 4) were digested at 16 hpi to release cells and CD11b^{+} cells sorted on columns to generate a CD11b^{-} fraction and a CD11b^{+} enriched fraction (G). Infectious virus released by each cell fraction after 6 hr in culture was quantified by plaque assay (H).
(I) Gene transcripts for bite-associated innate immune genes and viral RNA at 4 hpi ($n \geq 7$).
(J) Level of neutrophils at 4 hpi and monocytes at 18 hpi in the skin were determined by flow cytometry ($n \geq 4$).
(K and L) *Ccr2*$^{-/-}$ mice are protected from bite-enhanced arbovirus infection at 24 hpi. Wild-type and *Ccr2*$^{-/-}$ mice were infected with either 10^4 SFV4 (K) or 10^4 PFU of the genetically unrelated arbovirus BUNV (L) in the presence or absence of a mosquito bite.
(K) qPCR analysis of SFV RNA at 24 hr in the skin and viraemia at 24 hpi (n = 6).
(L) qPCR analysis of BUNV M-segment RNA at 24 hr in the inoculation site (skin), remote tissue (spleen), and viraemia at 24 hpi (n = 6).
All column plots show the median value ± interquartile range. $^{*}p < 0.05$, $^{**}p < 0.01$, $^{***}p < 0.001$, ns = not significant. Results shown are representative of either two or three experiments. See also Figure S7.

Received: August 20, 2015
Revised: March 10, 2016
Accepted: March 31, 2016
Published: June 21, 2016

REFERENCES

Bhatt, S., Gething, P.W., Brady, O.J., Messina, J.P., Farlow, A.W., Moyes, C.L., Drake, J.M., Brownstein, J.S., Hoen, A.G., Sankoh, O., et al. (2013). The global distribution and burden of dengue. Nature *496*, 504–507.

Bridgen, A., Weber, F., Fazakerley, J.K., and Elliott, R.M. (2001). Bunyamwera bunyavirus nonstructural protein NSs is a nonessential gene product that contributes to viral pathogenesis. Proc. Natl. Acad. Sci. USA *98*, 664–669.

Burt, F.J., Rolph, M.S., Rulli, N.E., Mahalingam, S., and Heise, M.T. (2012). Chikungunya: a re-emerging virus. Lancet *379*, 662–671.

Charmoy, M., Brunner-Agten, S., Aebischer, D., Auderset, F., Launois, P., Milon, G., Proudfoot, A.E.I., and Tacchini-Cottier, F. (2010). Neutrophil-derived CCL3 is essential for the rapid recruitment of dendritic cells to the site of *Leishmania major* inoculation in resistant mice. PLoS Pathog. *6*, e1000755.

Conway, M.J., Watson, A.M., Colpitts, T.M., Dragovic, S.M., Li, Z., Wang, P., Feitosa, F., Shepherd, D.T., Ryman, K.D., Klimstra, W.B., et al. (2014). Mosquito saliva serine protease enhances dissemination of dengue virus into the mammalian host. J. Virol. *88*, 164–175.

Cox, J., Mota, J., Sukupolvi-Petty, S., Diamond, M.S., and Rico-Hesse, R. (2012). Mosquito bite delivery of dengue virus enhances immunogenicity and pathogenesis in humanized mice. J. Virol. *86*, 7637–7649.

Déruaz, M., Frauenschuh, A., Alessandri, A.L., Dias, J.M., Coelho, F.M., Russo, R.C., Ferreira, B.R., Graham, G.J., Shaw, J.P., Wells, T.N.C., et al. (2008). Ticks produce highly selective chemokine binding proteins with antiinflammatory activity. J. Exp. Med. *205*, 2019–2031.

Dessens, J.T., and Nuttall, P.A. (1998). Mx1-based resistance to thogoto virus in A2G mice is bypassed in tick-mediated virus delivery. J. Virol. *72*, 8362–8364.

Edwards, J.F., Higgs, S., and Beaty, B.J. (1998). Mosquito feeding-induced enhancement of Cache Valley virus (Bunyaviridae) infection in mice. J. Med. Entomol. *35*, 261–265.

Elliott, R.M. (2014). Orthobunyaviruses: recent genetic and structural insights. Nat. Rev. Microbiol. *12*, 673–685.

Ferguson, M.C., Saul, S., Fragkoudis, R., Weisheit, S., Cox, J., Patabendige, A., Sherwood, K., Watson, M., Merits, A., and Fazakerley, J.K. (2015). Ability of the encephalitic arbovirus Semliki forest virus to cross the blood-brain barrier is determined by the charge of the E2 glycoprotein. J. Virol. *89*, 7536–7549.

Fontaine, A., Diouf, I., Bakkali, N., Missé, D., Pagès, F., Fusai, T., Rogier, C., and Almeras, L. (2011). Implication of haematophagous arthropod salivary proteins in host-vector interactions. Parasit. Vectors *4*, 187.

Gatherer, D., and Kohl, A. (2016). Zika virus: a previously slow pandemic spreads rapidly through the Americas. J. Gen. Virol. *97*, 269–273.

Gould, E.A., and Solomon, T. (2008). Pathogenic flaviviruses. Lancet *371*, 500–509.

Jabir, M.S., Ritchie, N.D., Li, D., Bayes, H.K., Tourlomousis, P., Puleston, D., Lupton, A., Hopkins, L., Simon, A.K., Bryant, C., and Evans, T.J. (2014). Caspase-1 cleavage of the TLR adaptor TRIF inhibits autophagy and β-interferon production during *Pseudomonas aeruginosa* infection. Cell Host Microbe *15*, 214–227.

King, J.G., Vernick, K.D., and Hillyer, J.F. (2011). Members of the salivary gland surface protein (SGS) family are major immunogenic components of mosquito saliva. J. Biol. Chem. *286*, 40824–40834.

Le Coupanec, A., Babin, D., Fiette, L., Jouvion, G., Ave, P., Missé, D., Bouloy, M., and Choumet, V. (2013). Aedes mosquito saliva modulates Rift Valley fever virus pathogenicity. PLoS Negl. Trop. Dis. *7*, e2237.

Limesand, K.H., Higgs, S., Pearson, L.D., and Beaty, B.J. (2000). Potentiation of vesicular stomatitis New Jersey virus infection in mice by mosquito saliva. Parasite Immunol. *22*, 461–467.

McCracken, M.K., Christofferson, R.C., Chisenhall, D.M., and Mores, C.N. (2014). Analysis of early dengue virus infection in mice as modulated by *Aedes aegypti* probing. J. Virol. *88*, 1881–1889.

Michlmayr, D., McKimmie, C.S., Pingen, M., Haxton, B., Mansfield, K., Johnson, N., Fooks, A.R., and Graham, G.J. (2014). Defining the chemokine basis for leukocyte recruitment during viral encephalitis. J. Virol. *88*, 9553–9567.

Moser, L.A., Lim, P.-Y., Styer, L.M., Kramer, L.D., and Bernard, K.A. (2015). Parameters of mosquito-enhanced West Nile virus infection. J. Virol. *90*, 292–299.

Nakamura, Y., Kambe, N., Saito, M., Nishikomori, R., Kim, Y.G., Murakami, M., Núñez, G., and Matsue, H. (2009). Mast cells mediate neutrophil recruitment and vascular leakage through the NLRP3 inflammasome in histamine-independent urticaria. J. Exp. Med. *206*, 1037–1046.

Peters, N.C., Egen, J.G., Secundino, N., Debrabant, A., Kimblin, N., Kamhawi, S., Lawyer, P., Fay, M.P., Germain, R.N., and Sacks, D. (2008). In vivo imaging reveals an essential role for neutrophils in leishmaniasis transmitted by sand flies. Science *321*, 970–974.

Pham, A.M., Langlois, R.A., and TenOever, B.R. (2012). Replication in cells of hematopoietic origin is necessary for Dengue virus dissemination. PLoS Pathog. *8*, e1002465.

Powers, A.M., Brault, A.C., Shirako, Y., Strauss, E.G., Kang, W., Strauss, J.H., and Weaver, S.C. (2001). Evolutionary relationships and systematics of the alphaviruses. J. Virol. *75*, 10118–10131.

Reagan, K.L., Machain-Williams, C., Wang, T., and Blair, C.D. (2012). Immunization of mice with recombinant mosquito salivary protein D7 enhances mortality from subsequent West Nile virus infection via mosquito bite. PLoS Negl. Trop. Dis. *6*, e1935.

Rodriguez-Andres, J., Rani, S., Varjak, M., Chase-Topping, M.E., Beck, M.H., Ferguson, M.C., Schnettler, E., Fragkoudis, R., Barry, G., Merits, A., et al. (2012). Phenoloxidase activity acts as a mosquito innate immune response against infection with Semliki Forest virus. PLoS Pathog. *8*, e1002977.

Ryman, K.D., and Klimstra, W.B. (2008). Host responses to alphavirus infection. Immunol. Rev. *225*, 27–45.

Schaeffer, E., Flacher, V., Papageorgiou, V., Decossas, M., Fauny, J.-D., Krämer, M., and Mueller, C.G. (2015). Dermal CD14(+) dendritic cell and macrophage infection by Dengue virus is stimulated by interleukin-4. J. Invest. Dermatol. *135*, 1743–1751.

Schledzewski, K., Falkowski, M., Moldenhauer, G., Metharom, P., Kzhyshkowska, J., Ganss, R., Demory, A., Falkowska-Hansen, B., Kurzen, H., Ugurel, S., et al. (2006). Lymphatic endothelium-specific hyaluronan receptor LYVE-1 is expressed by stabilin-1+, F4/80+, CD11b+ macrophages in malignant tumours and wound healing tissue in vivo and in bone marrow cultures in vitro: implications for the assessment of lymphangiogenesis. J. Pathol. *209*, 67–77.

Schmid, M.A., and Harris, E. (2014). Monocyte recruitment to the dermis and differentiation to dendritic cells increases the targets for dengue virus replication. PLoS Pathog. *10*, e1004541.

Schneider, B.S., Soong, L., Zeidner, N.S., and Higgs, S. (2004). *Aedes aegypti* salivary gland extracts modulate anti-viral and TH1/TH2 cytokine responses to sindbis virus infection. Viral Immunol. *17*, 565–573.

Schneider, B.S., Soong, L., Girard, Y.A., Campbell, G., Mason, P., and Higgs, S. (2006). Potentiation of West Nile encephalitis by mosquito feeding. Viral Immunol. *19*, 74–82.

Schneider, B.S., McGee, C.E., Jordan, J.M., Stevenson, H.L., Soong, L., and Higgs, S. (2007). Prior exposure to uninfected mosquitoes enhances mortality in naturally-transmitted West Nile virus infection. PLoS ONE *2*, e1171.

Schoggins, J.W., Wilson, S.J., Panis, M., Murphy, M.Y., Jones, C.T., Bieniasz, P., and Rice, C.M. (2011). A diverse range of gene products are effectors of the type I interferon antiviral response. Nature *472*, 481–485.

Serbina, N.V., and Pamer, E.G. (2006). Monocyte emigration from bone marrow during bacterial infection requires signals mediated by chemokine receptor CCR2. Nat. Immunol. *7*, 311–317.

Styer, L.M., Lim, P.Y., Louie, K.L., Albright, R.G., Kramer, L.D., and Bernard, K.A. (2011). Mosquito saliva causes enhancement of West Nile virus infection in mice. J. Virol. *85*, 1517–1527.

Supajatura, V., Ushio, H., Nakao, A., Akira, S., Okumura, K., Ra, C., and Ogawa, H. (2002). Differential responses of mast cell Toll-like receptors 2 and 4 in allergy and innate immunity. J. Clin. Invest. *109*, 1351–1359.

Tamoutounour, S., Guilliams, M., Montanana Sanchis, F., Liu, H., Terhorst, D., Malosse, C., Pollet, E., Ardouin, L., Luche, H., Sanchez, C., et al. (2013). Origins and functional specialization of macrophages and of conventional and monocyte-derived dendritic cells in mouse skin. Immunity *39*, 925–938.

Teo, T.-H., Lum, F.-M., Lee, W.W.L., and Ng, L.F.P. (2012). Mouse models for Chikungunya virus: deciphering immune mechanisms responsible for disease and pathology. Immunol. Res. *53*, 136–147.

Ulper, L., Sarand, I., Rausalu, K., and Merits, A. (2008). Construction, properties, and potential application of infectious plasmids containing Semliki Forest virus full-length cDNA with an inserted intron. J. Virol. Methods *148*, 265–270.

Weaver, S.C., and Lecuit, M. (2015). Chikungunya virus and the global spread of a mosquito-borne disease. N. Engl. J. Med. *372*, 1231–1239.

Wedmore, C.V., and Williams, T.J. (1981). Control of vascular permeability by polymorphonuclear leukocytes in inflammation. Nature *289*, 646–650.

Zompi, S., and Harris, E. (2012). Animal models of dengue virus infection. Viruses *4*, 62–82.

Immunity, Volume *44*

Supplemental Information

Host Inflammatory Response to Mosquito Bites Enhances the Severity of Arbovirus Infection

Marieke Pingen, Steven R. Bryden, Emilie Pondeville, Esther Schnettler, Alain Kohl, Andres Merits, John K. Fazakerley, Gerard J. Graham, and Clive S. McKimmie

Figure S1. In the absence of mosquito bites, SFV4 rapidly disseminates from skin inoculation sites to establish a peak viremia by 24 hours and activates the induction of type I IFNs in the draining popliteal lymph node (refers to Figure 1).

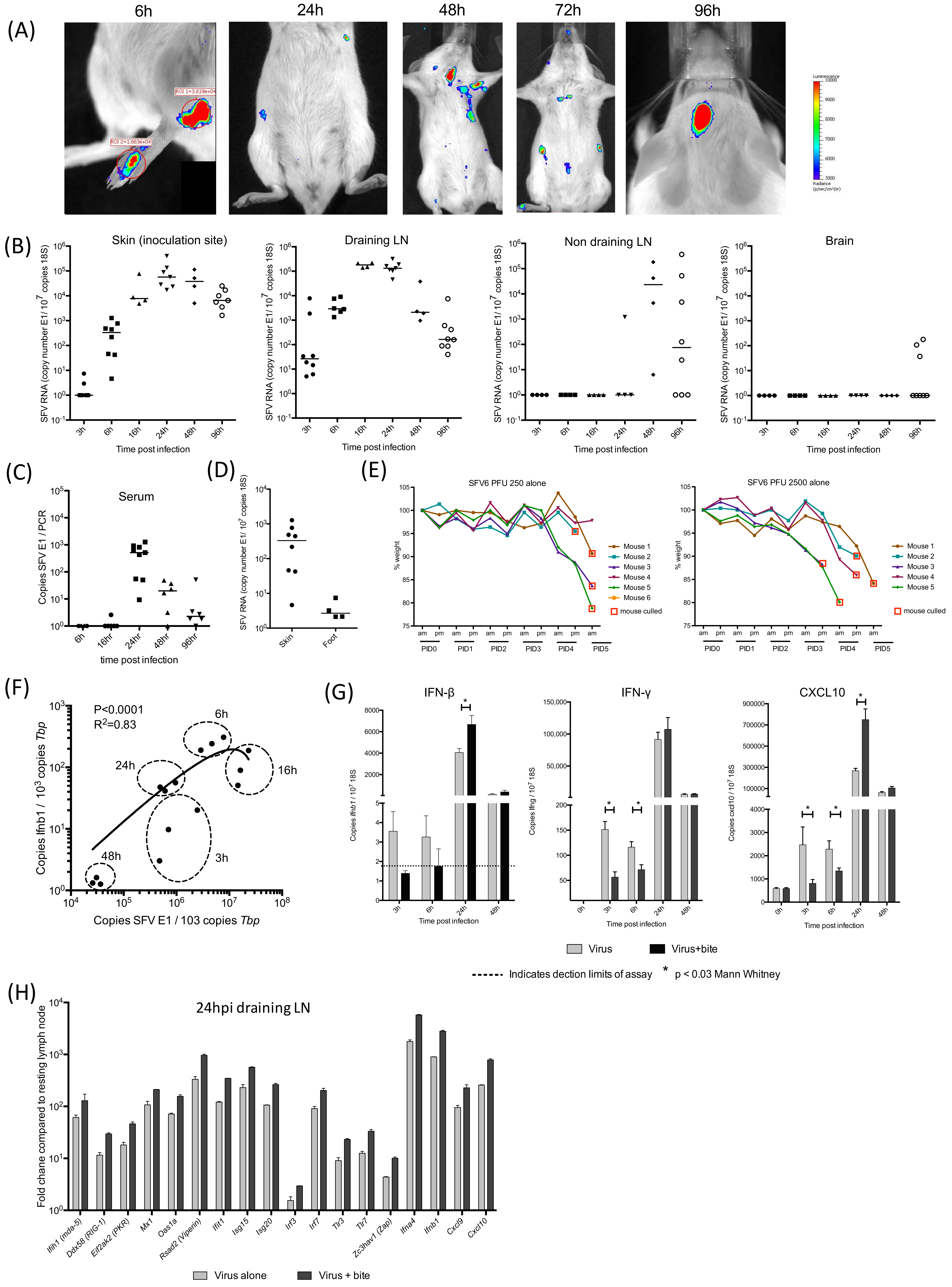

Figure S2. Fold change of innate immune transcripts in skin following mosquito bite or SFV4 infection alone (refers to Figure 2).

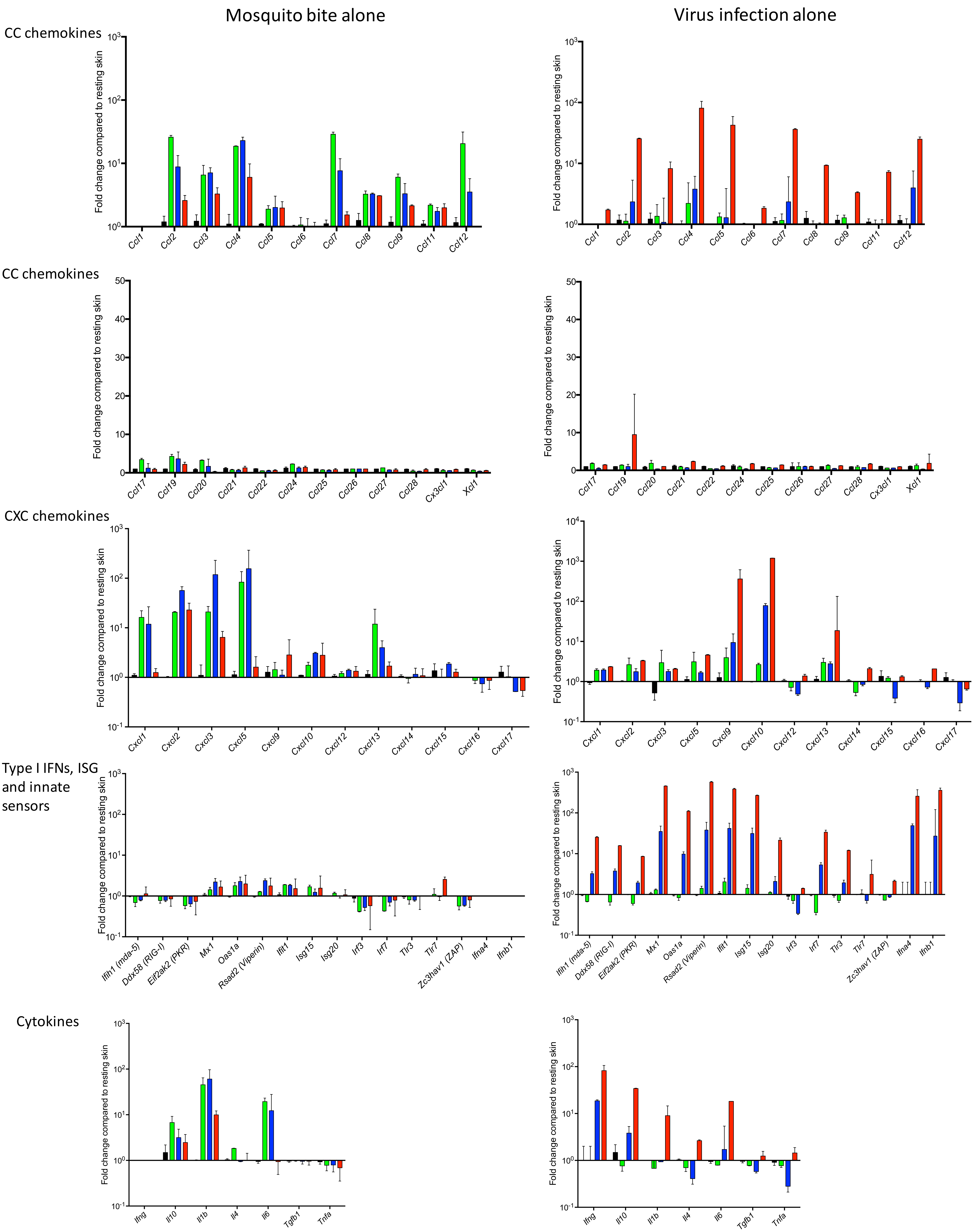

Figure S3. Mosquito bite-experienced mice exhibit similar gene expression changes at 6h following a new mosquito bite, compared to bite-naïve mice bitten for the first time (refers to figure 2).

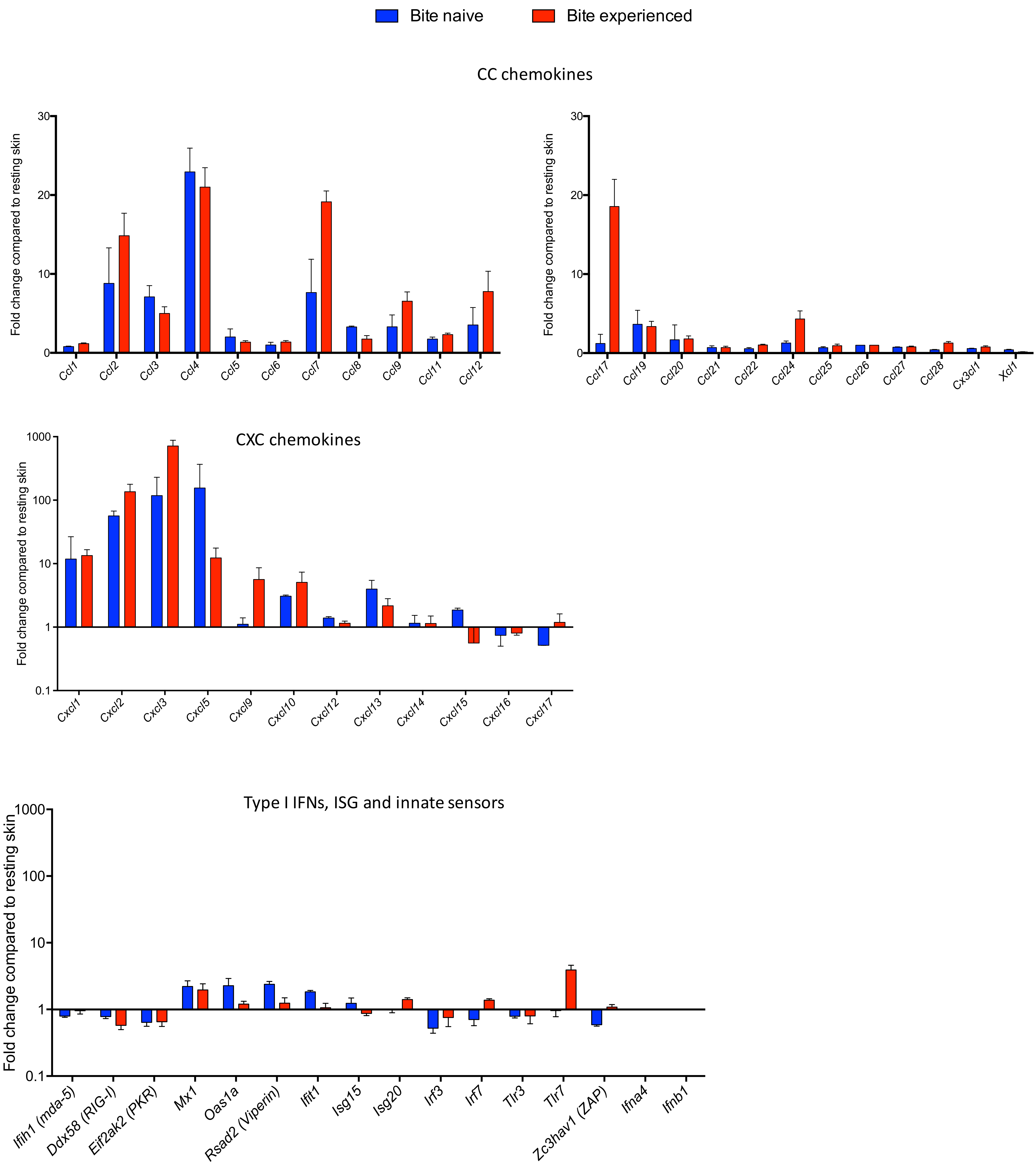

Figure S4. Cutaneous innate immune responses to mosquito bites and virus infection (refers to Figure 2)

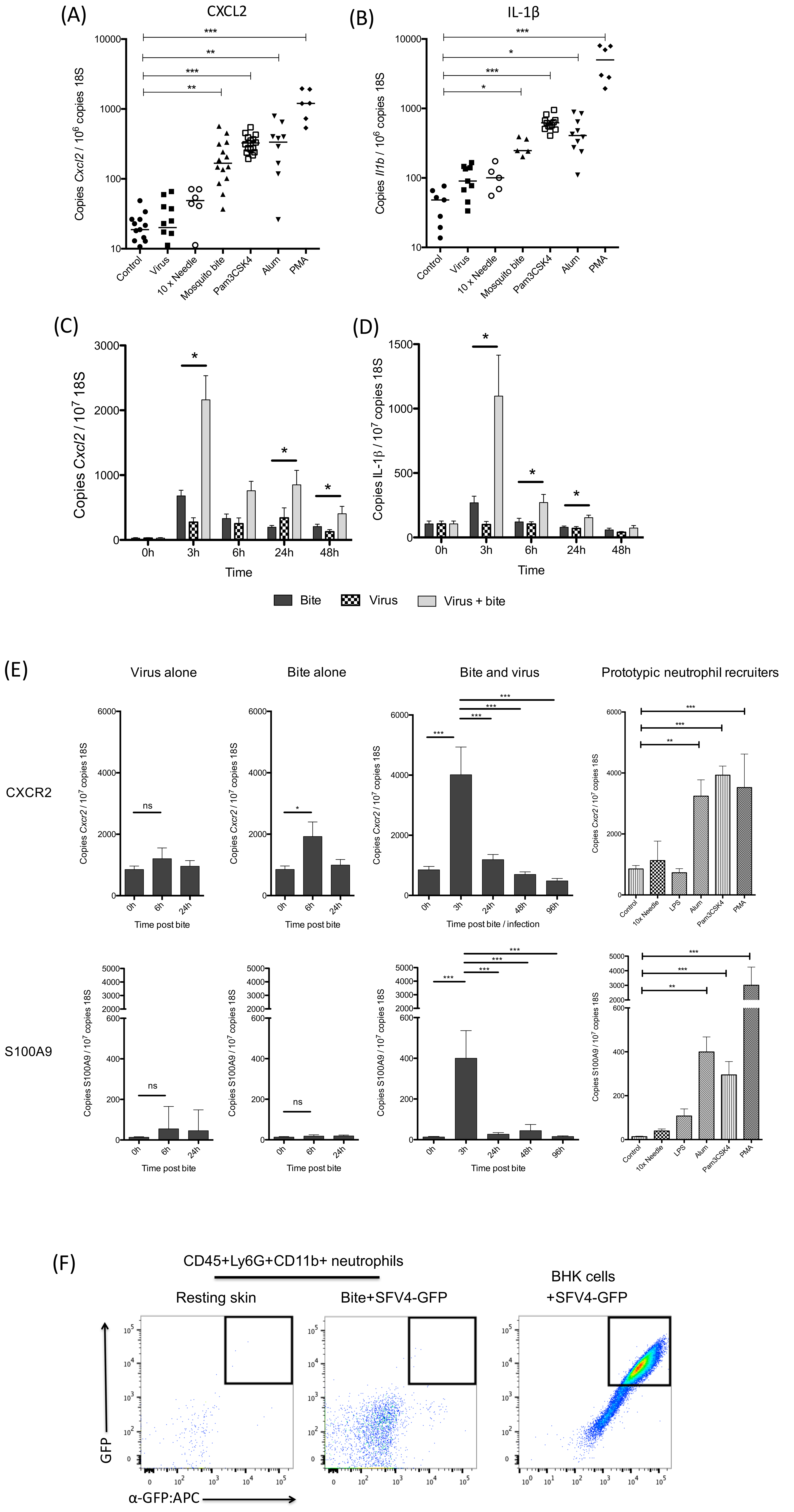

Figure S5. Neutrophils were depleted *in vivo* using the Ly6G antibody IA8 (refers to figure 4).

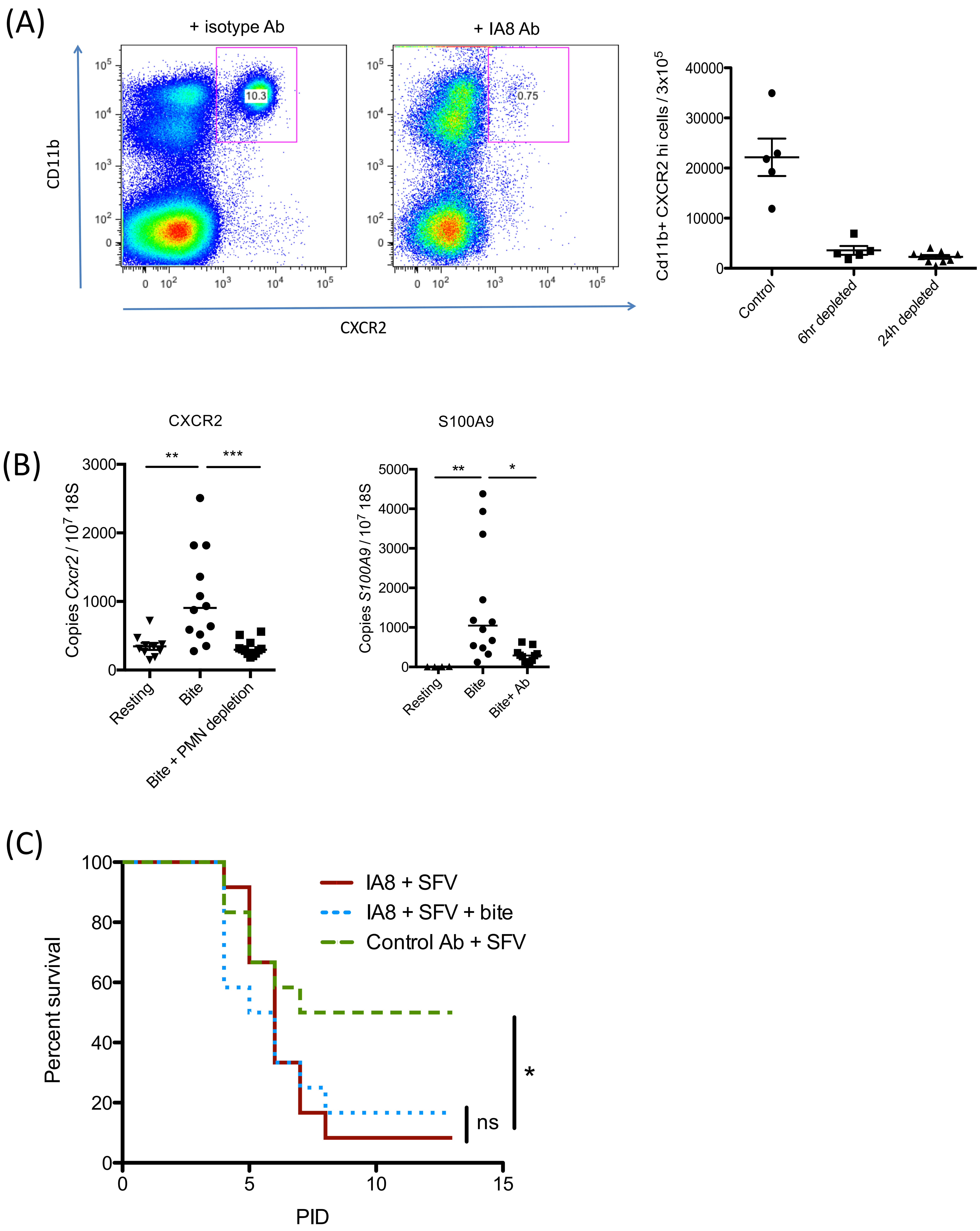

Figure S6. Pro-inflammatory agents enhance infection despite a pronounced type I IFN response (refers to figure 5).

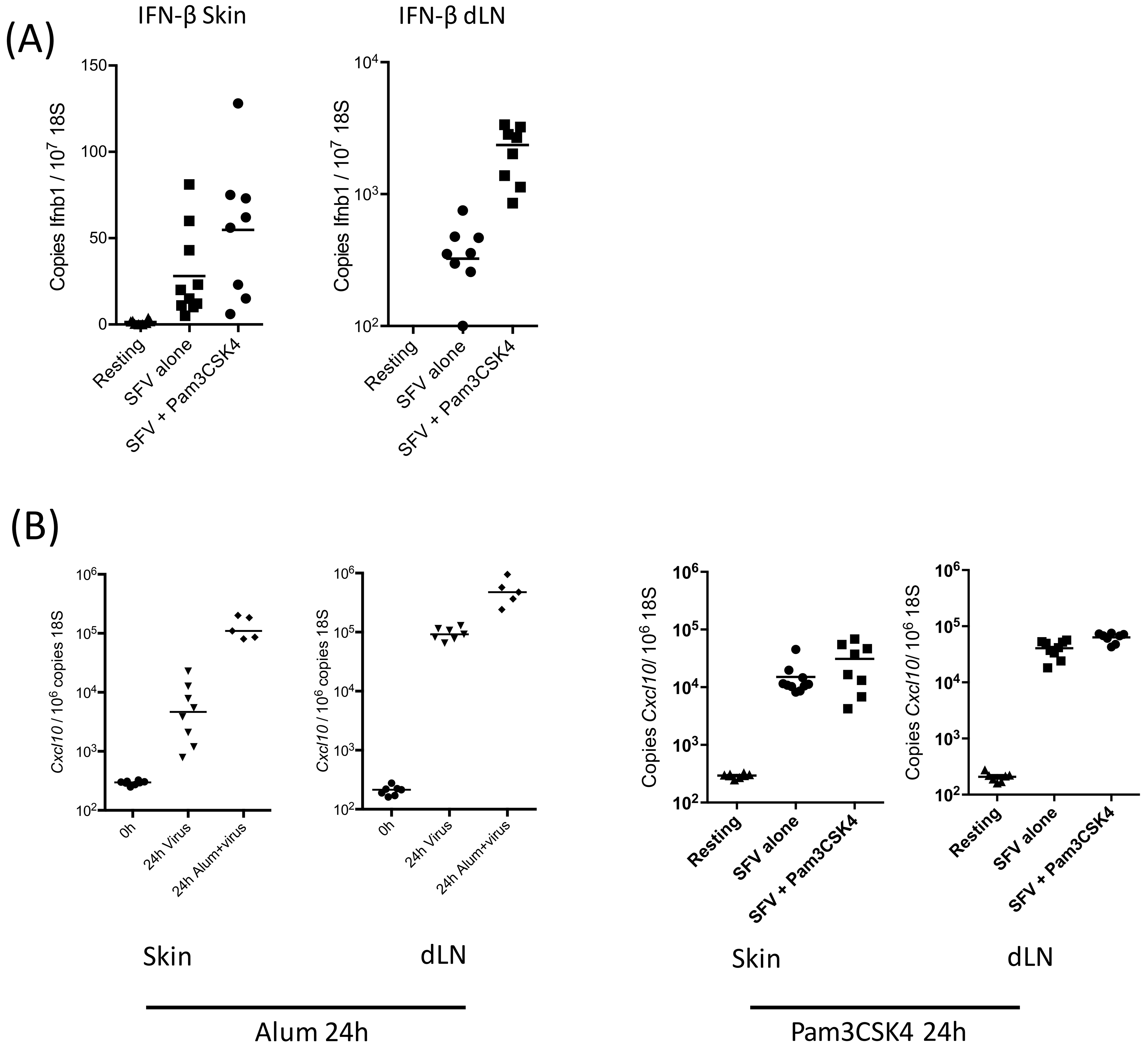

Figure S7. Leukocyte populations in the skin of mosquito bitten SFV-infected mice (refers to figure 6)

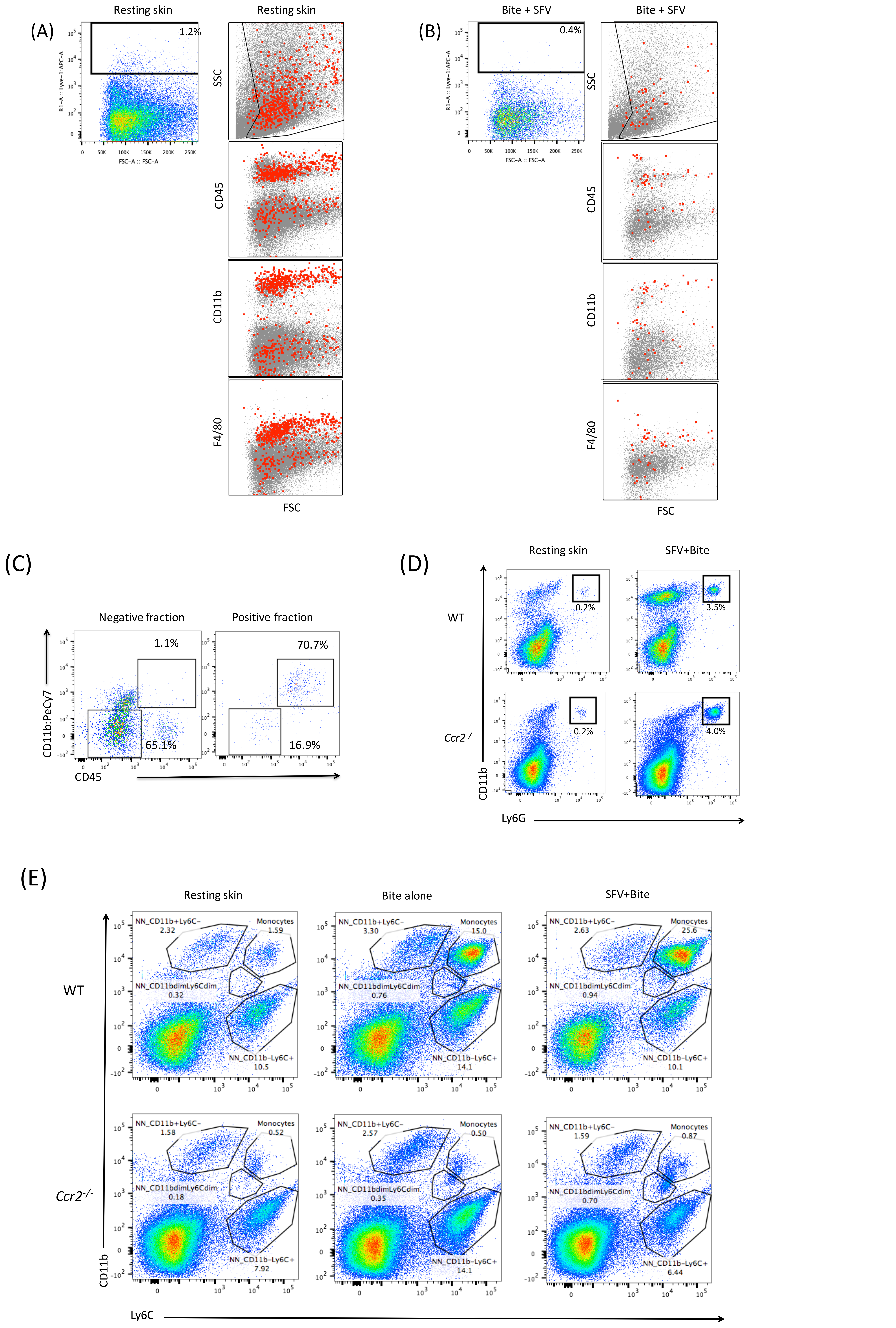

Supplementary information.

Supplementary figure legends.

Figure S1

In the absence of mosquito bites SFV4 rapidly disseminates from skin inoculation sites to establish a peak viremia by 24 hours and activates the induction of type I IFNs in the draining popliteal lymph node (refers to Figure 1).

(A) Mice were infected with SFV4(3H)-*RLuc* into the upper skin of the left foot. Following infection, RLuc activity was determined by intravital imaging (IVIS) (n=4). Injection of luciferase reagent in the tail vein resulted in a localized background signal and has been cropped from the image or masked using a black box.

(B-D) SFV4 replicates at local cutaneous inoculation sites and quickly disseminates to infect draining lymph nodes, followed by occasional spread to non-draining lymph nodes and the brain. Mice were infected with 10^3 PFU of SFV4 into the upper skin of the left foot and viral RNAs levels determined by qPCR. (B) The skin inoculation site, draining popliteal LN, non-draining contralateral popliteal LN and brain were dissected using fine biopsy tools and SFV E1 RNA and 18S quantified by qPCR. Those tissues positive for Renilla luciferase activity (Figure 1A) were also positive for SFV E1 RNA. (C) Serum was collected, cell-free RNA extracted and copies of the SFV RNA determined by qPCR. (D) SFV E1 RNAs were quantified for skin inoculation site and underlying tissue of the foot at 6h post infection, demonstrating that the majority of the virus inoculum had infected cutaneous tissue.

(E) Mice were infected with either 250 or 2500 PFU of virulent SFV6 s.c into skin of the foot. Weight of infected mice were measured twice a day until either the end of the experiment or until they reached clinically defined end points (denoted by a red square).

(F-H) Type I IFN responses in the infected draining popliteal LN were proportional to viral RNA levels and were delayed in mice subjected to mosquito bites. (F) SFV E1 RNAs correlated with IFN-β transcripts in the draining popliteal LN in a time dependent manner. Spearman's correlation was used to generate P and R^2 values. Gene transcripts were normalised to Tata Bind protein (TBP). (G) Mice were infected with SFV4 +/- mosquito bite and transcripts quantified by QPCR in draining popliteal LNs (H) Mice were similarly infected with SFV4 either in the presence (light grey) or absence of a mosquito bite (dark grey) and transcripts for type I IFNs and prototypic ISGs in the draining popliteal LN assayed by Taqman low density array at 24h post infection. Transcript numbers were normalized using 18S.

Figure S2

Fold change of innate immune transcripts in skin following a mosquito bite or SFV4 infection alone (refers to Figure 2).

Mice were subjected to mosquito bites alone or virus infection alone and transcript fold change quantified by Taqman low-density array. Fold changes was calculated by comparison to resting skin and grouped based on functional classes of genes; CC chemokines, CXC chemokines, cytokines and type I IFNs, ISGs and innate immune sensors. Bars show median value +/- interquartile range.

Figure S3

Mosquito bite-experienced mice exhibit similar gene expression changes 6h following a new mosquito bite, compared to bite-naïve mice bitten for the first time (refers to figure 2).

Mice were either left unbitten or bitten with mosquitoes, once a week for 4 weekd. Both roups of mice were then exposed to biting mosquitoes and gene transcript fold change determined by Taqman low-density array at 6 hours post bite. Bars represent median fold

change of bite-naïve mice (blue) and bite-experienced mice (red) compared to resting unbitten mice. Bars show median value +/- interquartile range.

Figure S4

Cutaneous innate immune responses to bites and virus infection (refers to figure 2).

(A,B) Copy numbers of transcripts for CXCL2 (A) and IL-1β (B) were determined by qPCR in mosquito bitten skin and in skin stimulated with known inducers of cutaneous neutrophil influx at 6h.

(C,D) CXCL2 (n=6) and IL-1β transcripts levels were determined by qPCR in skin following either mosquito bite alone, virus infection alone, or the combination of both.

(E) To better determine the kinetics of neutrophil entry and tissue residency we employed a highly sensitive qPCR-based strategy to quantify the expression of neutrophil markers, CXCR2 and S100A9. This analysis showed a robust, but highly transient increase in both CXCR2 and S100A9 expression, peaking at 3h post bite/infection, and which was comparable to the increases seen in skin following application of Alum, Pam3CSK4 or TPA at 6h. Bars show mean +/- SD. Statistical testing was undertaken using 1-way ANOVA and Tukey's multiple comparison post tests.

(F) To determine if virus could infect neutrophils recruited to the bite site, mice were bitten then infected with $5x10^5$ PFU of SFV4(Xho)-EGFP. Skin cells positive for $CD45^{hi}$, $CD11b^{hi}$ and $Ly6G^{hi}$ expression were gated by FACS and their positivity for SFV-GFP determined by measuring both GFP signal and also the signal generated by an anti-GFP:APC antibody. The intensity of GFP signal and α-GFP:APC staining was compared to that of infected BHK fibroblasts and suggested that neutrophils were not infected with SFV.

Figure S5

Neutrophils were depleted in vivo using the Ly6G antibody IA8 (refers to figure 4).

(A,B) The IA8 antibody was effective at depleting neutrophils from the systemic circulation and reduced the abundance of neutrophil specific markers in mosquito bitten skin. Neutrophils were depleted in vivo using the anti-Ly6G IA8 antibody and the number of circuiting and skin resident neutrophils compared to numbers in mice treated with a non-depleting isotype control antibody. (A) Circulating neutrophils ($CD11b^{hi}$ $CXCR2^{hi}$) in the plasma were quantified. (B) PMN infiltration into mosquito bitten skin of IA8-treated mice was analyzed by measuring the increase of neutrophil specific gene transcripts CXCR2 and S1A009 by QPCR at 3h post bite. Treatment with IA8 reduced the number of CXCR2 transcripts. Data points represent the values generated by individual mice.

(C) Depletion of neutrophils reduces survival to SFV6 infection irrespective of the presence of mosquito bites. Mice were depleted of neutrophils using IA8 and then infected with virulent SFV6, either in absence (red line) or presence of a mosquito bite (blue line). Non-bitten mice that had treated with the non PMN-depleting control 2A3 antibody, were also infected for comparison (green line), n=15.

Figure S6

Pro-inflammatory agents enhance infection despite a pronounced type I IFN response (refers to figure 5).

(A,B) Pam3CSK4 or Alum, when injected s.c. into the skin at the time of virus infection, does not suppress induction of anti-viral type I IFN or ISG induction by virus at 24hpi. Copy numbers of gene transcripts for IFN-β (A) and the prototypic ISG, CXCL10 (B), were determined by qPCR.

Figure S7

Leukocyte populations in the skin of mosquito bitten SFV-infected mice (refers to figure 7)

(A,B) The majority of lyve1+ cells analyzed by FACS were dermal macrophages. Cutaneous cells were analyzed by FACS following digestion of resting skin (A) and virus infected skin at 24h (B). Back-gating of all Lyve1^{hi} cells (red dots) reveals that the majority are CD45+CD11b+F4/80+ macrophages, and that these are depleted at 24 hpi with SFV.

(C) Success of our CD11b+ sorting of cutaneous cells. Mosquito bitten, virus infected skin was digested to release a single-cell solution, cells labelled with magnetic beads to CD11b and sorted on columns. The resulting negative and positive fractions were analyzed for FACS to characterize their purity.

(D, E) Leukocyte influx into skin of CCR2 null mice. (D) Neutrophil influx in the skin of CCR2 null mice was compared to that of wild type mice. Following SFV4 infection at mosquito bite sites, neutrophil influx at 3 hpi was similar in both wild type and CCR2 deficient mice. (E) Live cutaneous cells were gated to remove Ly6G^{hi} neutrophils and then analyzed for CD11b and Ly6C expression. Monocyte population was defined as CD11b^{hi}Ly6C^{hi}. Bites and SFV infection resulted in significant influx of monocytes in WT but not CCR2 null mice at 18hpi.

Extended Experimental Procedures

Cell culture, viruses and mice.

Ae. aegypti-derived AAG2 and *Ae. albopictus*-derived C6/36 mosquito cells were grown at 28°C in L-15 medium with 10% fetal calf serum, 10% tryptose phosphate broth, 100 units/ml penicillin and 0.1 mg/ml streptomycin. BHK-21 cells were grown in Glasgow minimum essential medium (GMEM) with 5% fetal calf serum, 10% tryptose phosphate broth, 100 units/ml penicillin and 0.1 mg/ml streptomycin at 37°C in a 5% CO_2 atmosphere. Two SFV strains were used: SFV4 is an avirulent strain that rarely triggers clinical disease, while highly SFV6 is highly virulent and causes lethal encephalitis within a week (Ferguson et al., 2015; Michlmayr et al., 2014). SFV4 was used to study viral dissemination and the local immune response, while SFV6 was used to study morbidity. Details of reporter viruses can be obtained from the authors. The pCMV-SFV4 backbone for production of SFV4 has been previously described (Ulper et al., 2008). The EGFP marker gene was inserted into the C-terminal region of nsP3 via a XhoI site naturally occurring in the genomic sequence (leading to expression of nsP3 fused to EGFP), while *Renilla* luciferase (RLuc) was inserted between duplicated nsP2 cleavage sites at the nsP3/4 junction as a cleavable reporter, using strategies previously described (Rodriguez-Andres et al., 2012). Plasmids containing the genomic sequence of wild type SFV and recombinant clones containing genetic markers (strains SFV4 and SFV6) were electroporated into BHK cells to generate infectious virus. All viruses were then passaged once in either *Aedes* AAG2 or C6/36 cells, purified from supernatant, and resuspended in Tris-NaCl-EDTA buffer as described, and titrated in BHK-21 cells by plaque assay (see below). Viruses were diluted in PBSA (PBS with 0.75% bovine serum albumin) to $1x10^7$ plaque forming units (PFU)/ml for injection into mice. Working stocks of wild-type BUNV were grown in BHK-21 cells at the lower temperature of 33°C, to decrease the frequency of defective viral particles, centrifuged to remove cell debris and virus titers were determined by plaque assays on BHK-21 cells (Bridgen et al., 2001).

Titration of virus stocks and quantification of viraemia *in vivo* were performed using plaque assays. Virus stocks, plasma or serum (as indicated in the Figure legends) were serially diluted in 0.75% PBSA and used to infect 70-80% confluent BHK-21 cells for 1 hour, and then overlaid with a 1:1 mixture of 1.2% Avicel RC-591 NF (kindly provided by FMC BioPolymer) in dH_2O and 2xMEM with 4% fetal calf serum, 200 units/ml penicillin and 0.2 mg/ml streptomycin. After 2 days incubation at 37°C/5% CO_2, cells were fixed using 10%

paraformaldehyde and stained with 0.1% toluidine blue. Viral titer was calculated based on number of plaques (Rodriguez-Andres et al., 2012).

7-week-old C57bl/6 and SCID mice were purchased from Charles River. CCR2 deficient mice were originally obtained from The Jackson Laboratory (stock number 004999) and bred in-house. *Il1r1*$^{-/-}$ mice were obtained from The Jackson Laboraory (B6.129S7-/l1r1^{tm1Imx}/J, stock number 003245). All mice were maintained under specific pathogen-free conditions at the Central Research Facility, University of Glasgow. All mice were housed in conventional (C57bl/6, *Ccr2*$^{-/-}$ deficient mice) or filter-topped cages (SCID and *Il1r1*$^{-/-}$ mice) and maintained in accordance with local and UK Home Office regulations.

Mosquito biting of mice and virus infection

Before mosquito biting, mice were anesthetized with an i.p. injection of KETASET (ketamine hydrochloride). To ensure mosquitoes bit a defined/restricted area of skin, the upper side of the foot was placed onto a mosquito cage containing 100 female *Ae. aegypti* mosquitoes (locally bred colony) and secured into place using tape. Up to 4 mice were similarly prepared per mosquito cage. The remainder of the mouse body surfaces, including toes and lower leg, were protected from probing mosquitoes by a textile and aluminium foil barrier. Mice were carefully monitored during mosquito biting, and a maximum of 5 mosquitos were allowed to engorge from the exposed area. Immediately after completion of mosquito biting (<5 minutes), the bitten skin was injected with a defined dose of virus in a small 1 μl volume. Allowing more than one mosquito to probe/bite the available skin surface ensured that most of the exposed skin was subjected to probing/bites. Thus, by clearly defining the number of mosquito bites to a restricted area of skin and by injecting a known titre of virus inoculum into this defined cutaneous site, it was possible to guarantee that virus was injected into either; i) mosquito bitten, or ii) resting unaffected skin. This approach enabled the effect of bites on concurrent virus infection to be quantifiably compared to virus infection alone in the absence of a bite. This comparison is not possible if using infected mosquitoes to infect mice; the inoculum supplied by biting mosquitoes was too variable and unpredictable to allow effective comparisons to needle inoculated virus in the absence of bites.

For virus infection, both in the absence or presence of mosquito bites, mice were anesthetized (KETASET injection (i.p.), or isoflurane by inhalation). 1 μl of virus in PBSA was injected into the skin of the upper side of the foot using either; 10^3 or 10^4 PFU SFV4, $2.5x10^2$ PFU SFV6 or $2.5x10^4$ PFU BUNV. Injections were undertaken using a Hamilton Syringe and custom-made point 4 style 33 gauge needles (Hamilton, Switzerland).

Mosquito saliva

Mosquitoes were starved for one day prior to salivation. Using a microscope, legs and wings of female mosquitoes were carefully dissected after which the proboscis was placed in a 10 µl tip containing 1 µl non-drying immersion oil (Cargille). Subsequently, mosquitoes were placed at 28°C and allowed to salivate for >1 hour. Successful salivation was confirmed by microscopy; saliva was visible as bubbles in the oil. The tips were briefly centrifuged to collect and combine saliva droplets. The saliva was further pooled by centrifugation of the oil-saliva mixture for 15 minutes, 1000xg at 4°C, after which excess oil was removed with the aid of a dissecting scope. Saliva was stored at -80°C until use. To investigate the influence of saliva on viral replication, 1ul 0.75% PBSA containing 10^4 PFU SFV4 with or without the saliva of 5 mosquitoes was injected in resting or bitten skin as described above.

Measurement of RLuc activity and tissue fluid accumulation in vivo

To monitor viral replication *in vivo*, mice were infected with SFV4 encoding RLuc as a non-structural protein. Virus encoded RLuc activity was assessed using RediJect Coelenterazine h (Perkin Elmer) and an Intravital Imaging System (IVIS Spectrum; Caliper Life Sciences) as per manufacturers instructions. Briefly, mice were anesthetized using isoflurane-inhalation and injected intravenously, in the tail vein, with 15 µg of Coelenterazine h before RLuc activity measurement for up to ten minutes post injection. Injection of Coelenterazine h resulted in substantial background signal at the site of intravenous injection in the tail. Accordingly, this injection site artifact was removed from images to prevent confusion with virus-derived signal elsewhere in the body.

To determine the extent of fluid accumulation and vascular leakage at mosquito bite sites, mice were i.p. injected with Evans blue dye before infection/bite. Level of fluid accumulation at infection/bite site at 3 hours post challenge was determined by colorimetric measurement (620 nm) of tissue-free dye concentration after soaking samples in formamide for 24 hours.

Depletion of neutrophils

To deplete neutrophils, mice were injected with antibodies that bind to Ly6G as per manufacturer's instructions (BioXcell). Briefly, mice were injected i.p. with 200µl of IA8 antibody or control antibody (2A3) at 4 days and 1 day before infection, which specifically and effectively depletes Ly6G$^+$ cells (Jamieson et al., 2012). Successful neutrophil depletion

was confirmed by flow cytometry analysis of peripheral blood using neutrophil markers CD11b^{hi} and CXCR2^{hi} and qPCR analysis of CXCR2 transcripts in skin (Figure S5).

RNA extraction and gene expression analysis

RNA was extracted using PureLink Plus columns and DNA digested on column as per manufacturer's instruction (Life technologies). Briefly, tissue samples were homogenised in TRIzol (Life Technologies) using a TissueLyser LT with 7mm metal beads (Qiagen), followed by purification using PureLink columns with DNase digestion (Life Technologies). Up to 2 μg RNA was converted to cDNA using the High Capacity RNA-to-cDNA kit (Life technologies). Gene expression analysis was undertaken using custom designed SYBR-green based qPCR assays using PerfeCTa (Quanta). Because of the expression strategy of alphaviruses and the nature of their positive-sense RNA genomes, the qPCR assay for SFV E1 measures the sum value of both genome and subgenomic RNA used for E1 gene expression. A selection of representative samples was additionally analysed using Taqman Low Density Arrays (TLDA). For TLDA analysis, the cDNA generated from the equivalent of 1μg of total RNA was loaded into a custom TLDA plate and samples assayed as per manufacturer's instructions (Applied Biosystems). For serums samples cell-free virus RNA was extracted and copies of the SFV RNA determined by qPCR. Because there is no housekeeping gene to reference, a standard volume (100ul) of serum was used, and 1/60 of the resulting cDNA loaded into each qPCR. All SYBR green and Taqman assays were undertaken on a 7900HT Real time machine (Applied Biosystems). Primer sequences are available upon request.

ELISAs were undertaken using Duoset kits (R and D systems). Briefly, tissues samples were immediately lysed in T-PER lysis solution in the presence of protease inhibitors (Roche) by shaking at 50Hz with a 7mm steel bead (Qiagen) for 10 minutes in the TissueLyser LT. Supernatants were clarified by centrifugation at 16,000g for 15 minutes at 4°C. Supernatants were diluted 1:2 with ELISA diluent and analysed using DuoSet as per manufacturers instructions (R&D systems).

Survival curves

After infection with either $2.5x10^{2}$ PFU SFV6 or 10^{5} PFU SFV L10, mice were monitored several times a day for development of clinical signs. Mice were culled when they reached clinically defined end-points of disease, as previously described (Fazakerley, 2002; Michlmayr et al., 2014).

Flow cytometery, magnetic cell separation, immunohistochemistry and histology

For flow cytometry, bitten skin of the foot or back was enzymatically digested to release cells using 1 mg/ml collagenase D (Roche), 0.5 mg/ml dispase (Roche) and 0.1 mg/ml DNaseI (Invitrogen) in Hanks' balanced salt solution (Sigma) at 37°C, 1300 RPM. Cells were stained using a subset of the following antibodies: CD45 (30-F11), CXCR2 (TG11), Ly6G (IA8), APC or PerCP streptavidin, GFP:APC (FM264G) (all Biolegend), CD11b (M1/70), F4/80 (BM8), Ly6G (RB6-8C5), Ly6C (HK1.4), Lyve-1 (ALY7) and pro-IL-1β (NJTEN3) or matching IgG1 K isotype control (P3.6.2.8.1) (all eBioscience). Furthermore, cells were stained with Fixable Viability Due eFluor780 (eBioscience) and fixed in 4% methanol-free paraformaldehyde (Thermo Scientific) or Cytofix/Cytoperm (BD) before analysis using a MACSQuant (Milteny). Analysis was performed using Flowjo version 8.8.7 or 10.0.7 (TreeStar). Cells were first gated to exclude doublets and dead cells, and then gated for CD45 or CD11b to remove stromal cells.

For magnetic cell separation, virus infected skin was enzymatically digested as described above. Cells were sorted based on CD11b expression as per manufacturers instructions (CD11b MicroBeads, clone M1/70, Miltenyi). Briefly, cells were labelled using 10 µl CD11b microbeads after which the positive and negative fraction were separated on MS columns. The positive and negative fraction were subsequently cultured in RPMI media 1640 (Invitrogen) with 10% fetal calf serum, 100 units/ml penicillin, 0.1 mg/ml streptomycin, 0.05 mg/ml gentamycin, 50 nM β-Mercaptoethanol and 2.5 mM HEPES. Virus release was measured in cell-free supernatant at various periods of culturing using plaque assays.

For immunohistochemistry, skin of the back was shaven before bite/infection. Skin was fixed for 24 hours in 4% methanol-free paraformaldehyde (Thermo Scientific) before freezing in OCT embedding medium (Tissue Tech) on dry ice. Sections of 8-10 µm were cut on a Shandon Cryotome (Thermo Scientific) and mounted onto Colorfrost Plus microscope slides (Thermo Scientific). After washing with PBS, a hydrophobic barrier was applied using an ImmEdge pen (Vector Laboratories) around the sections. Subsequently, sections were blocked in Tris-Saline-Tween (TBS)/5% fish gelatin (Sigma-Aldrich) for one hour, washed with TBS, and incubated overnight at 4°C with a primary antibody against Lyve-1 (affinity purified polyclonal goat IgG, R&D systems) or inflammatory macrophages (ER-HR3 conjugated to APC, BioLegend) in TBS/2% fish gelatin. Lyve-1 treated slides were washed three times in TBS before addition of the secondary chicken anti-goat IgG Alexa Fluor 647 conjugated antibody (Life Technologies) for 1 hour at 4°C. Sections were washed twice in TBS before mounting in Vectashield mounting medium with DAPI (Vector laboratories).

For histology, skin was fixed overnight in neutral buffered formalin (Leica) before progressive dehydration through increasing concentrations of ethanol to xylene (tissue processor Shandon Citadel 1000, Thermo Scientific). Skin samples were embedded in paraffin wax and 10 μm sections were cut and mounted onto Superfrost slides (Fisher Scientific). Haematoxylin and eosin staining was performed according to standard protocol. Sections were rehydrated in water via decreasing alcohol concentrations before a 7-minute stain in Haematoxylin Z (Cell path). Sections were rinsed in water, 1% acid alcohol, water, and immerged in Scott's Tap Water Substitute for 2 minutes. Sections were washed in water before a 4-minute stain in Putts Eosin (Cell Path), and washed in running water, 70% ethanol, 100% ethanol (2x), xylene (3x) before mounting using dibutyl phthalate xylene and visualization on a light microscope.

Statistical analysis

Data were analyzed using Prism Version 5 software. *In vivo* derived data from virus-infected mice was not normally distributed and was accordingly analyzed using the non-parametric based tests Mann-Whitney, Kolmogorov-Smirnov, or Kruskal-Wallis test with Dunn's multiple comparison. Unless otherwise stated all column plots have statistical significance indicated; *$p<0.05$, **$p<0.01$, ***$p<0.001$, ****$p<0.0001$, ns=not significant. Unless otherwise stated all column plots show the median value +/- interquartile range. Wherever possible, preliminary experiments were performed to determine requirements for sample size, taking into account the available resources and ethical use of animals. Animals (gender and age matched) were assigned randomly to experimental groups. For plaque assays, samples were coded and analyzed blind by a separate investigator. TLDA gene expression data was subjected hierarchal clustering in GeneSpring (Agilent) to generate heat maps. All survival curves were analyzed using the logrank (Mantel Cox) test. Correlation was calculated using Pearson. For qPCR data, each dot plotted represents the median of 4 technical replicates of one biological replicate. All results shown are representative of either two or three experiments. Importantly, biological replicates were excluded from analysis if s.c. or i.d. injection of virus inadvertently punctured a blood vessel.
